# Diploid chromosome-level reference genome and population genomic analyses provide insights into Gypenoside biosynthesis and demographic evolution of *Gynostemma pentaphyllum* (Cucurbitaceae)

**DOI:** 10.1093/hr/uhac231

**Published:** 2022-10-19

**Authors:** Xiao Zhang, Yuhe Zhao, Yixuan Kou, Xiaodan Chen, Jia Yang, Hao Zhang, Zhe Zhao, Yuemei Zhao, Guifang Zhao, Zhonghu Li

**Affiliations:** Key Laboratory of Resource Biology and Biotechnology in Western China (Ministry of Education), College of Life Sciences, Northwest University, Xi’an, Shaanxi, 710069, China; Key Laboratory of Resource Biology and Biotechnology in Western China (Ministry of Education), College of Life Sciences, Northwest University, Xi’an, Shaanxi, 710069, China; Laboratory of Subtropical Biodiversity, Jiangxi Agricultural University, Nanchang, 330045, China; Key Laboratory of Resource Biology and Biotechnology in Western China (Ministry of Education), College of Life Sciences, Northwest University, Xi’an, Shaanxi, 710069, China; College of Life Sciences, Shanxi Normal University, Taiyuan, Shanxi, 030012, China; Key Laboratory of Resource Biology and Biotechnology in Western China (Ministry of Education), College of Life Sciences, Northwest University, Xi’an, Shaanxi, 710069, China; Key Laboratory of Resource Biology and Biotechnology in Western China (Ministry of Education), College of Life Sciences, Northwest University, Xi’an, Shaanxi, 710069, China; College of Life Sciences, Sun Yat-sen University, Guangzhou, Guangdong, 510275, China; Key Laboratory of Resource Biology and Biotechnology in Western China (Ministry of Education), College of Life Sciences, Northwest University, Xi’an, Shaanxi, 710069, China; School of Biological Sciences, Guizhou Education University, Guiyang, Guizhou, 550018, China; Key Laboratory of Resource Biology and Biotechnology in Western China (Ministry of Education), College of Life Sciences, Northwest University, Xi’an, Shaanxi, 710069, China; Key Laboratory of Resource Biology and Biotechnology in Western China (Ministry of Education), College of Life Sciences, Northwest University, Xi’an, Shaanxi, 710069, China

## Abstract

*Gynostemma pentaphyllum* (Thunb.) Makino is a perennial creeping herbaceous plant in the family Cucurbitaceae, which has great medicinal value and commercial potential, but urgent conservation efforts are needed due to the gradual decreases and fragmented distribution of its wild populations. Here, we report the high-quality diploid chromosome-level genome of *G. pentaphyllum* obtained using a combination of next-generation sequencing short reads, Nanopore long reads, and Hi-C sequencing technologies. The genome is anchored to 11 pseudo-chromosomes with a total size of 608.95 Mb and 26 588 predicted genes. Comparative genomic analyses indicate that *G. pentaphyllum* is estimated to have diverged from *Momordica charantia* 60.7 million years ago, with no recent whole-genome duplication event. Genomic population analyses based on genotyping-by-sequencing and ecological niche analyses indicated low genetic diversity but a strong population structure within the species, which could classify 32 *G. pentaphyllum* populations into three geographical groups shaped jointly by geographic and climate factors. Furthermore, comparative transcriptome analyses showed that the genes encoding enzyme involved in gypenoside biosynthesis had higher expression levels in the leaves and tendrils. Overall, the findings obtained in this study provide an effective molecular basis for further studies of demographic genetics, ecological adaption, and systematic evolution in Cucurbitaceae species, as well as contributing to molecular breeding, and the biosynthesis and biotransformation of gypenoside.

## Introduction

*Gynostemma pentaphyllum* (Thunb.) Makino belongs to the family Cucurbitaceae and it is a perennial creeping herb with palmately compound leaves, dioecious flowers, and globose berries. This species is the most widely distributed plant in *Gynostemma* Bl., where it is mainly distributed in moist and warm mountainous forests in sub-tropical East and Southeast Asia, with a wide elevation range from 300 m to 3200 m [1]. In China, the wild plant resources of *G. pentaphyllum* are highly abundant in the Yangtze River Basin and its southern areas. Polyploidization is common and complex in *G. pentaphyllum* natural populations [2] and the mechanism responsible for the formation of its mixed-ploidy populations is still unclear. As a traditional Chinese medicinal plant, *G. pentaphyllum* is generally used due to its anti-oxidation, anti-cancer, and anti-inflammatory properties, as well as functioning in reducing blood fat and improving immunity because it contains many beneficial substances, such as saponins, flavonoids, andamino acids [3–11]. The plant tastes sweet and aromatic, and it can be taken either as tea or in alcohol [4]. Therefore, in recent years, *G. pentaphyllum* has attracted extensive attention from scientists in order to develop its great medicinal value and commercial potential.

**Figure 1:**
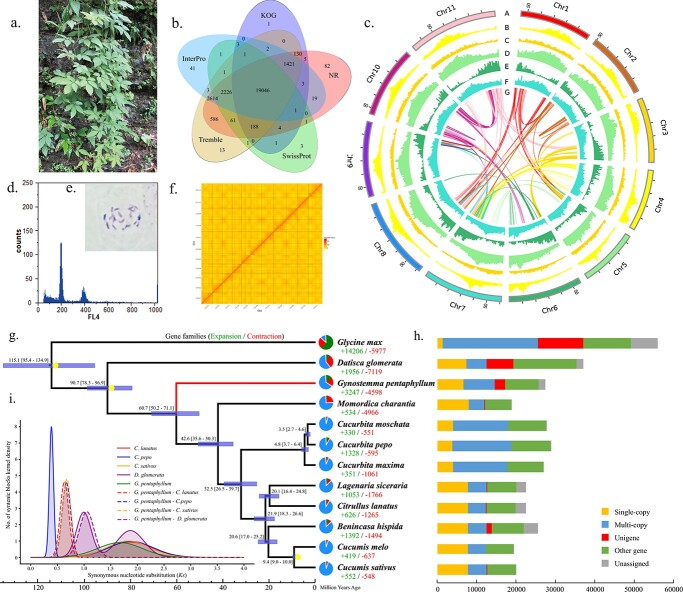
Plant morphology, karyotypes, assembled genome information, and evolutionary analyses of *G. pentaphyllum*. (a) Wild plant and habitat of *G. pentaphyllum*. (b) Venn diagram for the genome annotated based on five databases. (c) Circos graph showing the genome characteristics from the outer circle to the inner ring (A–G): (A) circular representation of the 11 pseudochromosomes featured in 10-Mb intervals across the genome; (B–D) distributions of (B) Ty3-gypsy long terminal repeat retrotransposons (LTR-RTs), (C) Ty1-copia LTR-RTs, and (D) all transposable elements; (E) gene density, (F) GC distribution, and (G) intraspecific collinearity. (d) Flow cytometry analysis. (e) Anatomical sections of *G. pentaphyllum* chromosomes. (f) Hi–C chromatin interaction heatmap for 11 pseudochromosomes in the *G. pentaphyllum* genome. (g) Inferred phylogenetic tree, divergence time, and gene family expansions and contractions. The numbers at the node positions represent the divergence time of each species in millions of years. The numbers in parentheses indicate the confidence interval for the divergence time, which can be used to estimate the divergence time of target species and other species. The yellow points are the calibration times used to correct the time of species divergence. The green, red, and blue sections in the pie graph indicate the expanded, contracted, and other gene families, including extinctions and no change gene families, respectively. The red and green numbers represent the exact precise quantities of expanded and contracted gene families, respectively. (h) Clusters of orthologous and paralogous gene families in *G. pentaphyllum* and 11 other sequenced plant genomes. (i) Whole-genome duplication (WGD) analysis of *G. pentaphyllum* and genomes of related species.

The genus *Gynostemma* is currently the only plant taxon containing ginsenosides other than the genus *Panax* L. Compared with *Panax* plants, *Gynostemma* has a high saponin content (about five times that of ginsenosides [12]), rich species resources, strong adaptability, short growth cycle, and it is easy to cultivate [7, 13, 14]. Since the isolation of the first two gypenosides in 1981, more than 200 gypenosides have been isolated and identified, and about 25% have similar structures to ginsenosides [15]. Thus, *Gynostemma* species are considered important resources for extracting ginsenosides. Previous studies suggested that gypenosides and ginsenosides are both damarane-type tetracyclic triterpenoid saponins [15], and that gypenosides are secondary metabolites in the synthesis pathway for triterpenoids [13]. However, further studies are still necessary, especially of the modification stage after triterpenoid saponin skeleton formation, which is an important stage that often leads to the generation of various gypenosides. Therefore, analyzing the biosynthetic pathway for gypenosides will help to further regulate the biosynthesis of gypenosides, and lay the foundations for obtaining gypenosides and their biotransformation into ginsenosides.

**Table 1:** Statistics and assembly BUSCO evaluation of the *G. pentaphyllum* genome

**Feature**	**Statistic**
Estimate of genome size	608.95 Mb
GC content	32.95%
N50 length (contig)	5.05 Mb
Number of contigs (> = 2 kb)	158
N50 length (scaffold)	56.38 Mb
Number of scaffolds (> = 2 kb)	18
Tandem repeats	45 479 485 bp (7.47%)
RepeatMasker	70 556 049 bp (11.59%)
RepeatProteinMask	57 563 766 bp (9.45%)
De novo	350 543 161 bp (57.57%)
Total	366 739 319 bp (60.23%)
Number of genes	27 418
Average mRNA length	3397 bp
Average CDS length	2350 bp
Average exon per gene	10
Average exon length	233 bp
Average intron length	115 bp
Complete BUSCOs	1310 (95.3%)
Complete and single-copy BUSCOs	1284 (93.4%)
Complete and duplicated BUSCOs	26 (1.9%)
Fragmented BUSCOs	9 (0.7%)
Missing BUSCOs	56 (4%)
Total BUSCO groups searched	1375 (100%)

However, *G. pentaphyllum* has now been listed as a Grade II Key Protected Wild Plant Species by the Chinese government due to the excessive harvesting of its wild resources, which had led to gradual decreases and the fragmented distribution of *G. pentaphyllum* wild populations [16]. Therefore, extensive investigations and studies of the population genetics of this species appear to be urgently required, which will contribute to the formulation of a strategy for conserving the population size but also genetic variations. Few previous molecular genetic studies of *G. pentaphyllum* have been conducted and most were based on molecular markers, such as Inter-simple Sequence Repeat (ISSR), Random Amplified Polymorphic DNA (RAPD), and Simple Sequence Repeats (SSR) [17–19], as well as plastid and nuclear DNA fragments sequences obtained by Sanger sequencing [20].

Whole genome sequences can provide important resources for studying the origin and evolution of plant species, as well as genetic variations, traits for crop improvement, and biosynthesis pathways [21–26]. Moreover, there are many potential advantages of using genotyping-by-sequencing (GBS) for resolving the evolutionary history and genetic structure of plant populations, and genomic selection [27–29].

Here, we report a high-quality diploid chromosome-level *G. pentaphyllum* genome obtained using next-generation sequencing (NGS), third-generation Oxford Nanopore Technologies, and Hi-C technologies. We also screened the changes in the expression levels of a series of candidate genes related to the biosynthesis pathway for gypenosides by comparative transcriptome sequencing analysis. Furthermore, *G. pentaphyllum* population genomics were investigated using genome-wide single nucleotide polymorphisms (SNPs) obtained through GBS combined with ecological niche comparison analyses to elucidate the evolution and demographic history of natural *G. pentaphyllum* populations in sub-tropical China. The results obtained in this study provide an important reference genome for further population evolution studies, as well as for the exploitation, utilization, and conservation of *G. pentaphyllum* species resources. Our findings also enrich the genomic sequences available in Cucurbitaceae to facilitate evolutionary studies of this important plant family. In addition, our findings contribute to a better understanding of the dynamic history of plant populations in East Asia.

## Results

### Genome Ploidy evaluation

According to flow cytometry and analysis of anatomical sections of root tips, a diploid *G. pentaphyllum* plant individual was identified (2n = 2x = 22; Figures 1d  and 1e). Based on NGS short reads, the characteristics of the genome were evaluated from a total of 101.74 Gb of clean data. Results obtained by k-er frequency analysis estimated the genome size of *G. pentaphyllum* as about 591–592 Mb, with a heterozygosis rate of 1.49–1.55% and 66.4–72.16% repeat sequences (Supplementary Data Figure S1, Supplementary Data Table S1), thereby indicating that the *G. pentaphyllum* genome is highly heterozygous and repetitive.

### Genome sequencing and assembly

After filtering the raw data obtained by Nanopore sequencing, 164.84 Gb of clean reads were used to refine the assembled genome representing a coverage depth of 275×. The Nanopore data were corrected and *ab initio* assembled into preliminary contigs with a contig N50 of 5.05 Mb. After combining with 132.07 Gb of Hi-C sequencing data and 101.74 Gb of short reads, the contigs were clustered into 11 pseudo-chromosomes (Figure 1f, Supplementary Data Table S2, and Supplementary Data Table S3), where 608.88 Mb was anchored, accounting for 99.99% of the assembled contigs. Finally, a genome of 608.95 Mb was obtained with a mean GC rate of 32.95% and a scaffold N50 of 56.38 Mb (Supplementary Data Tables S2 and S3). To assess the integrity and accuracy, 99.22% of the short reads were mapped to the genome (Supplementary Data Table S4). We then evaluated the completeness of this genome using Benchmarking Universal Single-Copy Orthologs (BUSCO) [30] and the Embryophyta odb10 database, which demonstrated that the genome was 95.3% complete and 93.4% of the single-copy orthologs were intact (Table 1), thereby indicating the high quality of the assembled genome. The characteristics of the 11 pseudo-chromosomes in *G. pentaphyllum* are shown in Figure 1c and Supplementary Data Table S3.

### Genome annotation

We annotated 366.74 Mb repeats (60.23% of the genome) in the *G. pentaphyllum* genome, and 361.07 Mb (59.30% of the genome) of these repeats were identified as transposable elements (Table 1 and Table 2). The most abundant repetitive sequences were long terminal repeat (LTR) retrotransposons, which accounted for 48.28% of the genome, followed by DNA (9.15%), long interspersed nuclear elements (1.82%), and short interspersed nuclear elements (0.05%; Table 2). In total, 27 418 protein-coding genes (CDSs) were predicted in the *G. pentaphyllum* genome. The average CDS length and exon number per gene were 2350 bp and 10, respectively (Supplementary Data Table S5). BUSCO evaluation showed that 91.5% of the Embryophyta odb10 gene set was completely covered by the genome annotations (Supplementary Data Table S6). In particular, 96.97% (26 588 of 27 418) of the predicted genes were functionally annotated in eight public databases (Supplementary Data Table S7), where 17 664 genes were shared in five databases (Figure 1b). Moreover, we identified non-coding RNAs including 110 miRNAs, 651 tRNAs, 1161 rRNAs, and 459 snRNAs (Supplementary Data Table S8).

**Table 2:** Genomic footprint of transposable elements in the genome of *G. pentaphyllum*

	**Repbase TEs**		**TE protiens**		**De novo**		**Combined TEs**	
Type	Length (Bp)	% in genome	Length (Bp)	% in genome	Length (Bp)	% in genome	Length (Bp)	% in genome
DNA	12 740 163	2.09	4 422 274	0.73	49 555 340	8.14	55 690 546	9.15
LINE	3 445 353	0.57	2 186 354	0.36	8 535 942	1.40	11 105 355	1.82
SINE	40 999	0.01	0	0.00	255 233	0.04	296 232	0.05
LTR	54 777 642	9.00	50 956 807	8.37	288 093 839	47.31	293 979 833	48.28
Total	71 004 157	11.66	57 565 435	9.45	346 440 354	56.89	361 071 966	59.30

### Comparative and evolutionary genomic analyses

Comparative genomic analyses were performed by comparing the *G. pentaphyllum* genome with the genomes of 11 other plant species comprising nine Cucurbitaceae species, one Datiscaceae species, and one Fabaceae species (Supplementary Data Table S9). In total, 187 702 gene families (orthogroups) comprising 333 354 genes were determined across the 12 species. In addition, we identified 2713 *G. pentaphyllum*-specific genes in these gene families (Figure 1h and Supplementary Data Table S10). Orthogroup gene statistics were calculated for each species, and the *G. pentaphyllum* genome contained fewer (6664) single-copy genes than most of the species compared, except for three *Cucurbita* species and *Glycine max* (Figure 1h, Supplementary Data Table S11).

Analysis of gene family expansion and contraction found that 3247 gene families expanded and 4598 gene families contracted, which accounted for 13.92% and 19.72% of all gene families, respectively (Figure 1g). Furthermore, 2242 genes in all of the significantly expanded gene families (*p* < 0.05) were enriched in 85 Kyoto Encyclopedia of Genes and Genomes (KEGG) pathways, such as “Plant–pathogen interaction,” “Tryptophan metabolism,” and “Isoflavonoid biosynthesis” (Supplementary Data Figure S2a and Supplementary Data Table S12). In the significantly contracted gene families (*p* < 0.05), 16 genes were enriched in seven KEGG pathways comprising “Benzoxazinoid biosynthesis,” “Phagosome,” “Phenylpropanoid biosynthesis,” “Tryptophan metabolism,” “Endocytosis,” “Biosynthesis of secondary metabolites,” and “Metabolic pathways” (Supplementary Data Figure S2b and Supplementary Data Table S13).

We constructed a phylogenetic tree based on the 12 species using 6108 genes from 509 single-copy gene families. The tree showed that *G. pentaphyllum* diverged earlier than other cucurbits in the Cucurbitaceae family, where the divergence time from *Momordica charantia* was 60.7 million years ago (Mya), with a confidence interval of 50.2–71.1 Mya (Figure 1g). The *Ks* distribution of orthologs suggested that a recent whole-genome duplication (WGD) event was absent in *G. pentaphyllum* as in other species in the Cucurbitaceae family, whereas *Cucurbita pepo* and *Datisca glomerata* underwent a recent WGD event (Figure 1i). Speciation event times were obtained using the calculated divergence time for *G. pentaphyllum* – *D. glomerata* at 90.7 Mya. The *Ks* value of *G. pentaphyllum* – *D. glomerata* was 1.05, which allowed us to calculate the age of an ancient WGD (*Ks* = 1.674) at 144.58 Mya according to the computational formula: T = *Ks*/2r. In addition, complex genome collinearity was observed among *G. pentaphyllum* and *C. lanatus*, *C. pepo*, and *C. sativus* (Supplementary Data Figure S3), thereby indicating that the genomes of these species had undergone considerable chromosomal rearrangement.

### Transcriptome sequencing and analysis of Gypenoside biosynthetic pathway genes

**Figure 2:**
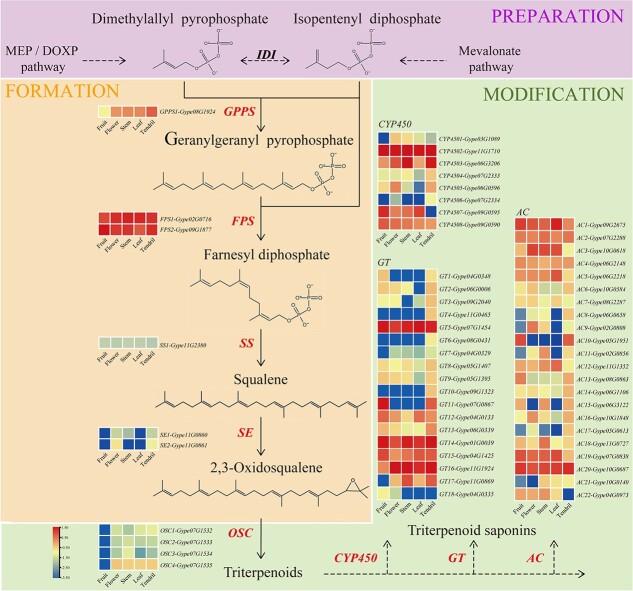
Comparative transcriptome analysis of genes involved in the gypenoside biosynthetic pathway. The expression value of each identified candidate gene is colored in log_10_(FPKM) in five tissues: fruit, flower, stem, leaf, and tendril. Low to high expression is indicated by the change in color from blue to red.

In total, 98.86 Gb of clean transcriptome data was obtained from 16 samples, and the average Q20 was 96.70% (Supplementary Data Table S14). Assembly and annotation were performed by mapping clean reads to the *G. pentaphyllum* genome. We screened out 788 genes involved in the gypenoside biosynthetic pathway. Comparative transcriptome analyses of five tissues (after averaging the replicate samples; Supplementary Data Figure S4) were conducted, and 58 differentially expressed genes (DEGs) were selected to assess the gene expression profiles (Figure 2 and Supplementary Data Table S15). The results identified six genes involved in the formation stage, including one *geranylgeranyl diphosphate synthase* (*GPPS*) gene, two *farnesyl diphosphate synthase* (*FPS*) genes, two *squalene synthase* (*SS*) genes, and one *squalene epoxidase* (*SE*) gene, where the *GPPS* and *FPS* genes were significant DEGs. In the modification stage that causes diversification, the cyclization reaction of 2,3-oxidosqualene catalyzed by 2,3-oxidosqualene cyclase (OSC) generates different types of triterpenoid skeletons in the first key modification step in gypenoside biosynthesis. Different types of triterpenoid saponin products are obtained after some chemical modifications with triterpenoid skeletons comprising oxidation, acylation, and glycosylation reactions. During these processes, cytochrome P450 (CYP450), glucosyltransferase (GT), and acyltransferase (AC) are the main modifying enzymes. Based on our results, we identified four *OSC* genes, and eight CYP450, 18 GT, and 22 AC genes were shown to be significant DEGs. In general, most of the genes were highly expressed in leaves and tendrils (Figure 2).

### Population genomic analyses

**Figure 3:**
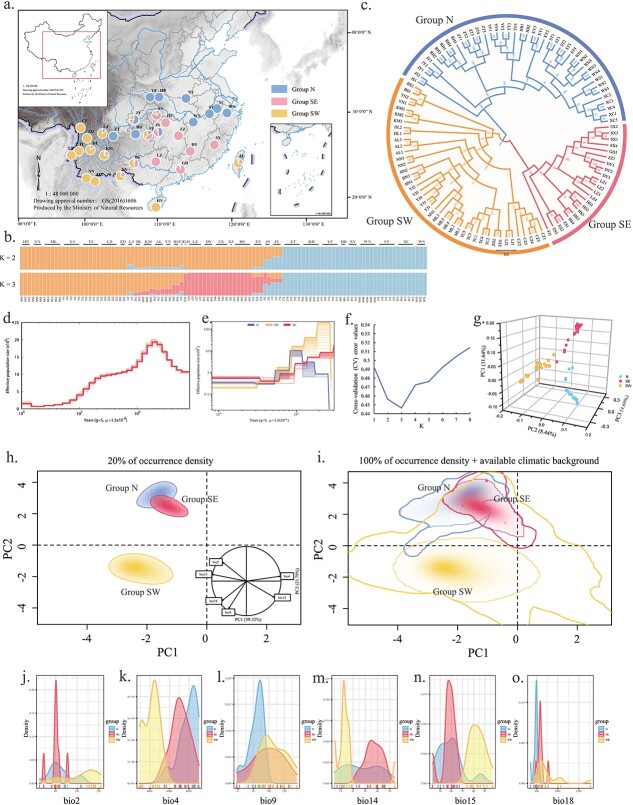
Genetic structure, demographic history, and global climatic space constructed over all background areas and realized niches of *G. pentaphyllum.* (a) Spatial genetic structure of *G. pentaphyllum* based on 32 populations within the natural species range in China. The pie chart colors indicate the probability of sample assignment based on SNPs analyzed using ADMIXTURE at the most likely K = 3. Standard maps produced by the Ministry of Natural Resources were used as the base maps with drawing approval numbers GS(2016)1554 and GS(2016)1606. (b) Results of ADMIXTURE analyses for 107 *G. pentaphyllum* individuals at K = 2 and K = 3. (c) Non-rooted maximum-likelihood phylogenetic tree based on SNPs in 107 *G. pentaphyllum* individuals. Color coding of the branches reflects the structure of genetic groups at K = 3. (d) Past effective population size history of *G. pentaphyllum* assessed by PSMC. (e) Demographic changes on recent timescales established for *G. pentaphyllum* by using SMC++. (f) Plot of cross-validation errors for ADMIXTURE runs with K values ranging from 1 to 8. (g) Three-dimensional principal component analysis (PCA) results for *G. pentaphyllum.* (h) The 20% occurrence density includes the contribution and direction of each variable to the first two components of PCA-env. (i) An occurrence density of 100% is denoted by the thin line and 100% of the available climatic background by the thick line. (j–o) Kernel density plots for six environmental parameters for *G. pentaphyllum* showing niche differences among the three groups.

To understand the genetic diversity, population structure, and demographic history, we performed population genomics analyses using GBS data for *G. pentaphyllum*. In total, 49.27 Gb of clean reads were obtained, with average Q30 and GC contents of 88.96% and 36.66%, respectively (Supplementary Data Table S16). We used the *G. pentaphyllum* genome as a reference to identify 31 805 biallelic SNP markers for further analysis after filtration from 3 978 979 raw SNPs. First, we assessed the genetic diversity of each individual. *G. pentaphyllum* had a low level of genetic diversity among populations. The observed heterozygosity (*Ho*) values ranged from 0.091 to 0.221, with an average value of 0.149, and the expected heterozygosity (*He*) ranged from 0.193 to 0.196, with an average value of 0.194 (Supplementary Data Table S17). Population structure inference was then performed based on 107 *G. pentaphyllum* individuals, which divided the 32 populations into three groups (Figures 3a and 3c) as the cross validation (cv) value was lowest at K = 3 (Figure 3f). In particular, the ADMIXTURE results showed that when K = 2, the 32 populations could be divided into two groups according to their geographic locations in the north and south (Group N and Group S, respectively), and when K = 3, Group N was stable but Group S was further divided into two groups designated as Group SE and Group SW, which geographically occupied the southeastern and southwestern regions, respectively (Figures 3a, 4b). Most populations were purely compositional but some were partially mixed (mosaic) in the Ta-pa Mountains–Wushan Mountains region and southeastern edge of Yunnan–Kweichow Plateau (Figure 3a). It should be noted that the AL population located on Taiwan island did not cluster with the adjacent main continent but instead it clustered together with Group SW. Analysis of molecular variance (AMOVA) detected significant genetic differentiation among the three groups (FST = 0.42, *p* < 0.001), but only 5.44% of the genetic variation was partitioned among the groups (Table 3).

We constructed a non-rooted branching maximum-likelihood (ML) tree to determine the phylogenetic relationships among 107 individuals and all individuals clustered into three main clades with high bootstrap values. Principal component analysis (PCA) also divided these individuals into three groups, where the three major PCs explained 24.77% (11.64%, 8.44%, and 4.69%, respectively) of the total variance (Figure 3g).

The effective population size of *G. pentaphyllum* determined based on pairwise sequentially Markovian coalescent (PSMC) analysis increased initially to ~2 × 10^5^ individuals but then started to decline around 1.1 Mya (Figure 3d). The results obtained by using the PSMC approach were quite limited in terms of the sample size and predictions for recent years, so we also determined the recent demographic history of *G. pentaphyllum* populations with SNP data using SMC++. For recent timescales, the global trend agreed with the PSMC results. However, further more refined tests found different changes in the effective population sizes of the three groups. In particular, Group SW had undergone continuous decline whereas Group SE expanded around 12–13 thousand years ago (ka). It was interesting that the change in the effective population size of Group N followed a pattern of expand–contract–expand, where the changes occurred at approximately 100 ka and 12 ka (Figure 3e). Analysis of the effective population sizes of the three groups at 10 ka showed that Group SE (~5500 individuals) was larger than Group N (~3500 individuals) and Group SW (~2000 individuals) (Figure 3e).

### Niche comparison analyses

The potential present distribution range predicted with MAXENT was consistent with the current geographic distribution of *G. pentaphyllum*, with area under the receiver operating characteristic curve values over 0.95 (Supplementary Data Figure S5). Compared with the present, the distribution range in the last interglacial (LIG) period was much wider, whereas that in the last glacial maximum (LGM) period was significantly restricted, and the overall historical dynamic trend comprised initial shrinkage and then expansion. However, the main distribution center areas were quite different, where they focused in eastern and southeastern China in the LIG period, before retreating to the Qinling and Ta-Pa Mountains in central China in the LGM period, and then expanding to the present distribution pattern mainly in the west and southwest (Supplementary Data Figure S5). Separate consideration of the three groups clearly indicated that the N and SE groups underwent dynamic changes consistent with most taxa, but Group SW was characterized by an *in-situ* expansion–contraction process (Supplementary Data Figure S5).

**Table 3:** Analysis of molecular variance (AMOVA) based on GBS data of *G. pentaphyllum*

**Source of variation**	**d.f.**	**Sum of squares**	**Variance components**	**Percentage of variation**	**Fixation Indices**
**2 Groups**					
Among groups	1	639.47	7.15128 Va	5.44	FSC: 0.39012^**^
Among populations within groups	30	7070.902	48.45045 Vb	36.89	FST: 0.42333^**^
Within populations	75	5680.75	75.74333 Vc	57.67	FCT: 0.05445^**^
Total	106	13391.121	131.34506		
**3 Groups**					
Among groups	2	1068.761	8.16692 Va	6.26	FSC: 0.38077^**^
Among populations within groups	29	6641.61	46.57533 Vb	35.69	FST: 0.41953^**^
Within populations	75	5680.75	75.74333 Vc	58.05	FCT: 0.06259^**^
Total	106	13391.121	130.48559		

Kernel density plots were generated to depict the frequency distributions of each group for six selected environmental variables, which showed that the three groups occupied different environments (Figure 3j–o). According to PCA-env analysis, the first two axes explained 83.11% of the total variation in the climatic conditions across the *G. pentaphyllum* population distributions for the three groups. The first component (PC1) and second component (PC2) explained 59.32% and 23.79% of the total variance, respectively (Figure 3h). PC1 was mainly explained by Bio 14 (temperature seasonality (STD*100)) and PC2 was explained by Bio 15 (precipitation seasonality (CV)) (Supplementary Data Figure S6). Multiple niche plots for the 20% occurrence density and metrics of niche dynamics obtained by comparing pairs showed that Group N and Group SE shared their climate niche to a great extent, whereas Group SW had a relatively independent niche (Figure 3h–o and Supplementary Data Table S18).

## Discussion

Cucurbitaceae is the fourth largest economically important edible botanical family in the world [31]. Many genomic studies have investigated gourds [22, 26, 32], but the early divergence of taxa in this family is still not fully understood. According to phylogenetic studies of a wide range of species, *G. pentaphyllum* is among the oldest taxa in the Cucurbitaceae family [31, 33] and its ploidy composition is complex [19]. Therefore, sequencing the diploid whole genome of *G. pentaphyllum* can help to obtain a deeper understanding the origin and evolutionary processes for the Cucurbitaceae family, as well as providing a reference for further studies of the mechanism associated with the development of mixed polyploidy *G. pentaphyllum* populations.

In this study, we successfully sequenced and assembled a chromosome-level high-quality diploid *G. pentaphyllum* genome and anchored the sequences to 11 pseudo-chromosomes, which corresponded to the karyotype (2n = 2x = 22). Compared with a previous genome assembly for this species, the contig N50, scaffold N50, and BUSCO values were all significantly improved (Table 1), and the final genome size (608.95 Mb) was larger than that of the previous assembly (582 Mb [34]). In the Cucurbitaceae family, the genome size of *G. pentaphyllum* is similar to that of *Sechium edule* (606.42 Mb [35]) and smaller than that of *Benincasa hispida* (859 Mb [22]), but much larger than most gourd genomes, such as those of *C. lanatus* (367.91 Mb [26]), *Lagenaria siceraria* (313.4 Mb [36]), and *Cucurbita maxima* (271.4 Mb [37]) (Supplementary Data Table S9). In total, 26 588 (96.97% of the predicted genes) CDSs were annotated with functions in *G. pentaphyllum*. The number of predicted genes in *G. pentaphyllum* is less than those in most gourd genomes [32] excluding watermelon [38], snake gourd [39], and bottle gourd [36].

Based on comparisons with 11 related plant species, our gene family results identified 2713 *G. pentaphyllum*-specific genes (Figure 1h, Supplementary Data Table S10), where 3247 gene families expanded and 4598 gene families contracted (Figure 1g). The expansion and contraction of gene families are regarded as key drivers of adaptive evolution and stress tolerance in various species [40–42]. Further analyses could focus on the associated molecular evolutionary mechanisms and functional analyses of these genes families.

WGD events are of great significance for species diversification, morphological diversity, and the acquisition of new functions during evolution [42, 43]. Previous studies have demonstrated that Cucurbitaceae plants underwent four WGD events, where an early large-scale cucurbit-common tetraploidization (CCT) occurred shortly after the ancient WGTγ event (approximately 130–150 Mya) shared by all core eudicots, and three recent WGDs within some genera in the tribe Cucurbitaeae, tribe Sicyoeae, and tribe Gomphogyneae^23^ [33, 35, 39, 44, 45],. However, we did not detect the CCT event and no recent WGD events were found in *G. pentaphyllum*, which are consistent with previous studies based on whole genome data [34, 39]. These differences might have been caused by the use of different data set types (such as transcriptome or genome data) and analytical approaches. In addition, the quite low rates of preserved CCT collinear genes in cucurbit genomes might have led to the failure to detect the CCT event [44]. The peak *Ks* distribution (at 144.58 Mya) evaluated for *G. pentaphyllum* overlapped with the time of the ancient WGTγ event (Figure 1i). Therefore, we suggest that the complex ploidy within *G. pentaphyllum* may have been the result of adaptation to different ecological niches by this widely distributed species, which will be another key focus of our future research. The phylogenetic tree and estimated divergence time indicated that *G. pentaphyllum* diverged earlier than other cucurbits in the Cucurbitaceae family, where the divergence time from *M. charantia* was around 60.7 Mya. This result supports a previous conclusion that the genus *Gynostemma* originated in the Early Tertiary Period [46].

The evolution of a species is largely related to its genetic diversity. According to our results, the genetic diversity of *G. pentaphyllum* was low and similar to that determined in previous genetics research using microsatellite molecular markers [19]. In addition, *G. pentaphyllum* is a dioecious perennial herbaceous plant that mainly reproduces asexually in a suitable environment. Long-term clonal growth will inevitably lead to decreased genetic diversity within a population and increased genetic differences between populations. Moreover, low diversity may be related to the population dynamics history of the species.

Current genetic structure patterns are produced by evolutionary and demographic processes at different temporal scales [47]. Plant palaeoecology reconstruction provides fundamental guidance for testable phylogeographic hypotheses, but it cannot indicate the detailed population history [48]. Ecological niche analysis can be used to predict the distributions of species under the impact of climate change and overcome the limitation due to inadequate fossil records in East Asia [49]. Combining ecological niche analysis with molecular data can enhance our understanding of population dynamics in the past and future [50]. In the present study, PCA and phylogenetic analysis of individuals identified three genetic groups, which were highly consistent with those obtained by ADMIXTURE clustering analysis (Figures 3c and 3g). According to our results, most of the populations exhibited very large geographical differences, and purely compositional differences might have been the result of founder or bottleneck effects [19]. However, the three groups were partially mixed (mosaic phenomenon) in the Ta-pa Mountains–Wushan Mountains region (ES, JS, FJ, and JY populations) and the southeastern edge of Yunnan–Kweichow Plateau (BS, YN, and KM populations; Figure 3a), thereby indicating that genetic exchanges had occurred between these populations throughout their evolutionary history. We propose that these areas might have been glacial refuges or recent differentiation centers for *G. pentaphyllum* during the LGM period [46].

In its early demographic history, the effective population size of *G. pentaphyllum* increased initially and then started to decline around 1.1 Mya (Figure 3d). The recent changes in the three groups differed to some extents (10–100 ka, Figure 3e). According to MAXENT distribution simulations, *G. pentaphyllum* populations underwent shrinkage initially before then expanding after the LIG period (130 ka, Supplementary Data Figure S5). At the start of the LGM period (21 ka), the Earth’s climate was extremely cold and vast areas at high latitudes were covered by continental glaciers [51–54]^.^ Thus, *G. pentaphyllum* populations retreated to the southeastern edge of the Qinghai–Tibet Plateau, Yunnan–Kweichow Plateau, and Qinling–Ta-pa Mountains areas. After the ice age, the temperature gradually warmed and herbaceous plants that preferred the humid environment gradually flourished. The *G. pentaphyllum* populations expanded from two large refuges and migrated along both sides of the rivers. The northern population in the Qinling–Ta-pa Mountains spread along the Yangtze River to the middle and lower reaches of the Yangtze River plain, whereas the southern population on the Qinghai–Tibet Plateau and Yunnan–Kweichow Plateau moved southward along the Pearl River to south China, thereby forming the current distribution pattern. In addition, our results indicated that the eastern edge of the Yunnan–Kweichow Plateau and the Ta-pa–Wushan Mountains area, which are located at the boundary of the second and third steps of topography in China [55], played very important roles in the formation of the genetic structure of *G. pentaphyllum* in subtropical China in a similar manner to that of the genetic structure of *Quercus fabri* in the same area [56]. The kernel density based on six selected environmental variables and E-space analysis indicated that Group N and Group SE partly shared their climate niche, whereas no niche overlap was found for Group SW (Figure 3h–o). Ecological differentiation provides preconditions for the differentiation of lineages following initial spatial isolation and also leads to adaptation to their corresponding environmental conditions to some degree [57]. Thus, the differences in the climatic conditions and ecological niches for the three groups over time eventually led to heterotopic differentiation of the three groups. Overall, our results indicate that geographic and climate factors shaped the genetic structure of *G. pentaphyllum*.

Gypenoside is the main active substance in *G. pentaphyllum*, and it has remarkable medicinal and nutraceutical properties [15]. Based on recent studies of the gypenoside biosynthesis pathway [12] [34], we explored the expression levels in different tissues of specific genes encoding enzymes that regulate each step in the gypenoside biosynthesis pathway. Most of the genes had higher expression level in the leaves and tendrils, as also suggested in a previous study [12] and these findings were consistent with the detection of the highest gypenoside content in the leaves of *G. pentaphyllum* [58]. Therefore, analyzing genes in the gypenoside biosynthesis pathway could facilitate further regulation of gypenoside biosynthesis, and provide the foundations for obtaining gypenoside and its biotransformation into ginsenosides.

In conclusion, in this study, we obtained a high-quality diploid chromosome-level whole genome sequence of *G. pentaphyllum* and compared it with the genomes of related taxa, thereby enriching the genome information available regarding the early differentiation of species in Cucurbitaceae. Our findings provide a basis for further phylogenetic studies of Cucurbitaceae, particularly understanding the mechanism associated with the formation of complex mixed polyploidy in *G. pentaphyllum*. Moreover, genome-level population genetics were investigated using a large number of populations within the distribution areas, which further improved our understanding of the population genetic diversity in *G. pentaphyllum*. By comparing the ecological niches among groups, we determined the population dynamics history of *G. pentaphyllum* and the mechanism associated with the formation of its genetic structure, thereby providing a reference for the rational utilization and development of *G. pentaphyllum* and species diversity protection. In addition, the identification of potential genes in the gypenoside synthesis pathway provides a strong theoretical basis for improving the gypenoside content through molecular breeding in the future.

## Materials and methods

### Plant materials

A female individual *G. pentaphyllum* was collected from Qingcheng Mountain (30°56′N, 103°34′E) in Sichuan Province, China, and cultured at the Evolutionary Botany Laboratory of Northwest University. Root tips were immersed in FAA fixative solution and used for chromosomal evaluation. Young buds aged 7 days were frozen at −80°C after processing in liquid nitrogen for Hi-C sequencing. Fresh leaves aged 15 days were used for flow cytometry analysis. Healthy leaves were placed in silica gel to dry rapidly and used for total DNA extraction.

Sixteen fresh samples of five tissues (four young leaves, four stems, three tendrils, four flowers, and one fruit) were also collected from the Qingcheng Mountain population and stored at −80°C.

Healthy leaves from 107 wild individuals in 32 populations were collected from the main distribution area of *G. pentaphyllum* in China (Figure 3a and Supplementary Data Table S13), and dried rapidly in silica gel. A handheld GPS (GarmineTrex Handheld GPS; Garmin) was used to determine the latitude and longitude of each site. Voucher specimens for the samples were deposited at Northwest University (Xi’an, Shaanxi, China).

### Genome Ploidy evaluation

Due to the prevalence of polyploidy within *G. pentaphyllum* species, the chromosomal ploidy of samples should be determined first. Chromosome karyotyping was conducted using anatomical sections and flow cytometry to identify authentic diploid *G. pentaphyllum* plants. Root tips were soaked in Kano fixing fluid for 24 h, before suspending in 1.0 mol/L HCl for 15 min, dyeing with modified carbolic–basic fuchsin liquid, tableting in the conventional manner, and observing by optical microscopy at 100× to count the chromosome numbers. Flow cytometry was conducted using a Partec CyFlow Space system by Jindi Future Biotechnology (Beijing, China).

### DNA isolation

Total DNA was extracted from dry leaves using the modified CTAB method and stored at 4°C. DNA samples for genome sequencing were sent to BGI-Shenzhen (Shenzhen, China) for NGS, Nanopore library construction, and sequencing, and the genomic DNA samples for GBS were sent to Novogene (Beijing, China).

### Genome sequencing

The short-read (PE 150 bp) NGS data were sequenced on the DNBSEQ™ platform. The total DNA was randomly fragmented and then sequenced after quality control. The raw reads were filtered using SOAPnuke v1.6.5 [59] to remove low quality reads, adapter pollution, and PCR duplication. The Nanopore (20 kb) library was constructed for long-read sequencing on the PromethION Beta v19.05.1 platform (Oxford Nanopore Technologies, Oxford, UK). The raw reads were quality filtered with thresholds comprising: minimum read length > 5000 bp and Q value of read >7. The high quality reads (clean reads) were then used to assemble the genome.

### Genome size estimation

The genome size of *G. pentaphyllum* was assessed by k-mer frequency analysis using NGS short reads. After filtering using SOAPnuke v1.6.5 [59], the optimal k-mer size was analyzed by KmerGenie [60]. The k-mer counts were then analyzed using Jellyfish [61] and GenomeScope [62] was employed to fit of the frequency spectrum, before estimating the genome size, heterozygosity, and repeat sequences.

### Genome assembly

Genome assembly was conducted as described previously [63] using Necat v0.01 [64], Racon v1.3.3 [65], Medaka v0.7.1, Pilon v1.23 [66], and purge haplotigs [67]. The Burrows–Wheeler Aligner (BWA) v0.7.8 [68] and Samtools v1.9 [69] were used to align filtered short fragments to the assembled genome sequence, before applying BamDeal v0.22 to obtain statistics regarding the GC distribution and depth with a window of 10 kb.

### Hi-C sequencing

The Hi-C library was prepared using chromatin from the nuclei of young buds, which were fixed with formaldehyde and extracted. High-throughput sequencing was performed for each qualified library. Raw data were also filtered using SOAPnuke v1.6.5 [59]. Valid interaction pairs were identified and extracted from the clean Hi-C data, and then aligned to the genome sketch using Juicer [70]. Based on the alignment results, clustering, sorting, and orientation were performed to assist with genome anchoring into chromosomes using 3D-dna [71] software. The assembled chromosome-level genome was split into bins of 100 kb to construct an interaction heatmap for validation and manual correction. The completeness of the assembled genome was assessed based on the Embryophyta odb10 database using BUSCO v4.0.6 [30] with the default parameters. To assess the genomic integrity and accuracy, the short reads were also mapped back onto the assembled genome using BWA [68].

### Genome annotation

We employed *de novo* and homology-based approaches to annotate the repeat sequences. RepeatMasker v. 4.0.7 [72] and RepeatProteinMask were used to align the transposable elements against the RepBase library (RepBase 21.01 [73]). In the *de novo* method, RepeatScout [74], Piler [75], RepeatModeler-2.1, and LTR_FINDER v. 1.07 [76] were used to construct a *de novo* repeat sequence library, and the repeat sequences were identified using RepeatMasker against this library. In addition, tandem repeats were isolated using Tandem Repeats Finder v. 4.09 [77] and LTR elements were investigated with LTR_FINDER v. 1.07 [76]. Finally, the overlapping repeat sequences obtained by using the two methods were merged together.

*Ab initio*, homolog, and transcriptomic evidence based strategies were used to predict the gene structure using Maker v. 2.31.8 [78] (details in Supplementary Data Note S1). Final gene sets were counted and the numbers of genes supported by the three prediction methods were retained. Finally, the completeness of the gene set was assessed based on comparisons with the plant single-copy ortholog gene database (Embryophyta_odb10) using BUSCO [30].

The gene sets obtained from gene structure annotation were searched against eight known protein databases (NCBI non-redundant protein (NR/NT), Gene Ontology (GO), KEGG, Clusters of Orthologous Groups (COG), SwissProt, InterPro, and TrEMBL) using BLAST v2.2.31 [79] with an e-value less than 1e-5.

### Comparative genomic and evolutionary analyses

Comparative genomic and evolutionary analyses were performed among *G. pentaphyllum* and 11 related species (Supplementary Data Table S9). All-vs-all BLAST analysis was conducted based on protein sequences from all 12 species using BLASTP v2.2.26 with an e-value of 1e-5, and OrthoMCL v2.0.9 [80] was then used to perform gene family clustering. In total, 6108 orthologous genes were collected from 509 single-copy gene families and aligned with Mafft [81]. MrBayes v3.1.2 [82] and PhyML [83] were used to construct the phylogenetic tree based on the second bases in these gene codons (phase 1 site), and TreeBeST was employed for root determination. MCMCTREE in the PAML v4.5 [84] package was used to estimate the molecular clock (substitution rate) and the divergence times among species by using *G. max* as an outgroup. Fossil calibration points from Timetree [85] were used to inform the species divergence times.

Genome collinear blocks were identified using MCScanX [86] with the default parameters, where each contained at least 20 and five collinear gene pairs when comparing different chromosomes in the genome and among species, respectively. Collinearity plots were generated using the JCVI program. To identify possible WGD events, we compared the genome of *G. pentaphyllum* with those of *C. lanatu* [87], *C. pepo* [88], *C. sativus* [89], and *D. glomerata* [90], and calculated synonymous nucleotide substitutions (*Ks* values) between syntenic genes. The *Ks* values between syntenic homologous gene pairs were calculated using WGDI v0.5.1 [91] and the timings of WGD events were calculated with the following formula: divergence date T = *Ks*/2r [92].

In addition, we assessed the contraction and expansion of gene families. First, species-specific gene families were filtered based on previous results for gene families. Second, CAFÉ [93] was used to estimate gene family contraction and expansion in species based on gene birth and apoptotic models, and combined with the divergence times. Finally, genes that significantly expanded or contracted (*p* ≤ 0.05) were selected for KEGG pathway enrichment.

### Transcriptome sequencing and data analyses

Total RNA was extracted using the ethanol precipitation protocol and CTAB-PBIOZOL reagent according to the instructions provided with the manual, and quantified with a Nano Drop and Agilent 2100 Bioanalyzer (Thermo Fisher Scientific, MA, USA) by BGI-Shenzhen (Shenzhen, China). Stranded RNA libraries were constructed for each sample and sequenced on the MGISEQ-2000 platform to generate paired end 150 bp reads (PE150). After sequencing, the data were filtered using SOAPnuke.

Clean reads were then mapped onto the *G. pentaphyllum* genome using HISAT2 [94], and the data obtained were assembled using Stringtie v2.1.4 [95]. The expression value of each gene measured as the fragments per kilobase of transcript per million mapped fragments (FPKM) [96] was calculated using the R package ballgown. The correlations were analyzed among 16 sample replicates (four young leaves, four stems, three tendrils, four flowers, and one fruit) using the R package Heatmap2. All of the assembled unigenes were annotated by aligning to the *G. pentaphyllum* genome. Genes involved in the gypenoside biosynthesis pathway were screened out and differential expression values were calculated using the limma package. Genes with adjusted |log2FC| > 1 and *p*-value <0.05 were defined as DEGs. TBtools [97] was employed to create an expression level heatmap for different tissues based on log_10_(FPKM) data.

### GBS sequencing and SNP calling

The GBS library was constructed using a one-enzyme system with the restriction enzyme *MseI* for cutting and adjusted with the enzyme *EcoR1*, before sequencing on the HiSeq X10 platform using a 150 bp paired end protocol to obtain a large number of raw reads. The clean data were obtained after strict filtration by removing sequences with barcodes, adapters, low quality bases (≤ 5 bp), and unmeasured base N (10% of single reads) from the raw reads. The two technical replicates for each sample were merged before variant calling as described by Wong et al. [98].

According to the sequence similarity, the remaining high quality reads were mapped onto the *G. pentaphyllum* genome using BWA [68] and the alignment results were sorted with Samtools [69]. SNP calling from multiple samples was performed using Genome Analysis Toolkit (GATK) v3.4 [99]. We obtained SNPs by using a simplified approach that only retained biallelic SNPs, as described previously for *Medicago sativa* [24]. Loci with low polymorphism (MAF < 0.05) and that exhibited significant deviation from Hardy–Weinberg equilibrium (*p* < 0.01) were filtered from the data set using Vcftools [100] and Plink v1.7 [101]. Finally, successfully genotyped loci in at least 95% of cases and present in all individuals were retained for further population genetics analyses.

### Population genomics analyses

The population genetic diversity and structure were investigated with the method described in Supplementary Data Note S2. An individual-based ML phylogenetic tree was built using RAxML v8.0.5 [102]. We used the GTRGAMMA model with ascertainment bias correction and a rapid bootstrap procedure with 1000 replicates. PCA was performed with GCTA v1.26 [103] and the first three eigenvectors were plotted. AMOVA was conducted using Arlequin v3.5 [104] based on the identified SNPs and the data sets determined with the ADMIXTURE analysis results. Moreover, we used the PSMC [106] method with whole genome data to infer the history of effective population size changes in *G. pentaphyllum* on older timescales. The SMC++ [105] method tailored to handle large data sets to determine demographic changes on recent timescales was also applied to reconstruct the recent demographic history using GBS data. We employed a universal per-generation mutation rate of 1.5e−8 mutations per base pair for all dicots [ 107–109]. In addition, according to our field investigations over many years, a constant generation time of 5 years was used for demographic analysis.

### Niche comparison analyses based on environmental space (E-space) and geographical space (G-space)

In order to capture the different ecological spaces occupied by the three groups, we performed comparative analyses in both the environmental (E) and geographical (G) spaces, which complemented each other in niche comparison analyses [110, 111]. The historical and present geographical distribution ranges of each *G. pentaphyllum* group were recovered from population sampling sites using MAXENT v.3.3.3k [112]. Models were developed based on their present geographic distributions and current environmental factors, and then projected onto the LIG (130 ka) and the LGM (21 ka) periods. Six climatic variables identified in a previous study [19] were selected as data predictors (Supplementary Data Note S3), and bioclimatic variables were downloaded from the WorldClim website. The LGM data used in this study were under the Community Climate System Model [113]. In addition, kernel density plots for each group according to the six environmental variables were generated using the R package ggplot2.

To evaluate the degree of niche overlap, occurrence and climate data were translated into environmental axes (PCA-env) by PCA using R scripts [114, 115]. The ecological backgrounds of the three groups (Group N, Group SW, and Group SE defined by the ADMIXTURE program) were selected from a minimum convex polygon with a buffer size of 0.3 degrees [116]. To construct the available environmental space for the two principal axes, the values of the six climatic factors were extracted and the densities of population occurrences were calculated using a kernel smoothing method [111] Finally, we obtained multiple range PCA-env plots to represent all available climates and occupied conditions for occurrence densities of 20% and 100%. Moreover, niche overlaps between groups were characterized by niche stability, niche unfilling, and niche expansion [114, 115].

## Supplementary Material

Web_Material_uhac231

## Data Availability

The whole genome sequence data including NGS short reads, Nanopore long reads, Hi-C interaction reads, transcriptome data, and genome file have been deposited in the NCBI database under BioProject: PRJNA810200.

## URLs

SOAPnuke v1.6.5 (https://github.com/BGI-flexlab/SOAPnuke)

Medaka v0.7.1 (https://github.com/nanoporetech/medaka)

Samtools v1.9 (https://github.com/samtools)

BamDeal v0.22 (https://github.com/BGI-shenzhen/BamDeal)

RepeatMasker (http://repeatmasker.org)

RepeatProteinMask (http://www.repeatmasker.org/cgi-bin/RepeatProteinMaskRequest)

RepeatScout v 1.0.6 (http://bix.ucsd.edu/repeatscout/)

RepeatModeler-2.1 (http://www.repeatmasker.org/RepeatModeler/)

KEGG (http://www.genome.jp/kegg/)

GO (http://www.geneontology.org/)

SwissProt (http://www.uniprot.org/)

InterPro (https://www.ebi.ac.uk/interpro/)

TrEMBL (http://www.uniprot.org/)

## References

[1] Chen S , Lu A, Charles J. Flora of China Vol. 19. Beijing: Missouri Botanical Garden Press; 2011.

[2] Gao XF , Chen SK, Gu Z et al. A chromosomal study on the genus *Gynostemma* (Cucurbitaceae). *Acta Bot Yunnanica*. 1995;17:312–6.

[3] Li Y , Lin W, Huang J et al. Anti-cancer effects of *Gynostemma pentaphyllum* (thunb.) makino (jiaogulan). *Chin Med*. 2016;11:43. 27708693 10.1186/s13020-016-0114-9 PMC5037898

[4] Yin F , Hu L, Pan R. Novel dammarane-type glycosides from G*ynostemma pentaphyllum*. *Chemical & Pharmaceutical Bulletin*. 2004;52:1440–4. 15577241 10.1248/cpb.52.1440

[5] Tsai YC , Lin CL, Chen BH. Preparative chromatography of flavonoids and saponins in *Gynostemma pentaphyllum* and their antiproliferation effect on hepatoma cell. *Phytomedicine*. 2010;18:2–10. 21036575 10.1016/j.phymed.2010.09.004

[6] Xie Z , Liu W, Huang H et al. Chemical composition of five commercial *Gynostemma pentaphyllum* samples and their radical scavenging, antiproliferative, and anti-inflammatory properties. *J Agric Food Chem*. 2010;58:11243–9. 20939605 10.1021/jf1026372

[7] Razmovski-Naumovski V , Huang THW, Tran VH et al. Chemistry and pharmacology of *Gynostemma pentaphyllum*. *Phytochem Rev*. 2005;4:197–219.

[8] Lin CC , Huang PC, Lin JM. Antioxidant and hepatoprotective effects of *Anoectochilus formosanus* and *Gynostemma pentaphyllum*. *Am J Chin Med*. 2012;28:87–96. 10.1142/S0192415X00000118 10794120

[9] Wang M , Wang F, Wang Y et al. Metabonomics study of the therapeutic mechanism of *Gynostemma pentaphyllum* and atorvastatin for hyperlipidemia in rats. *PLoS One*. 2013;8:e78731. 24223845 10.1371/journal.pone.0078731 PMC3815346

[10] Gao D , Zhao M, Qi X et al. Hypoglycemic effect of *Gynostemma pentaphyllum* saponins by enhancing the Nrf2 signaling pathway in STZ-inducing diabetic rats. *Arch Pharm Res*. 2016;39:221–30. 25066072 10.1007/s12272-014-0441-2

[11] Faradianna LE , Gu HF, Nazaimoon W et al. Evaluation of antidiabetic fffects of the traditional medicinal plant *Gynostemma pentaphyllum* and the possible mechanisms of insulin release. *Evid Based Complement Alternat Med*. 2015;2015:120572. 26199630 10.1155/2015/120572 PMC4493304

[12] Chen Q , Ma C, Qian J et al. Transcriptome sequencing of *Gynostemma pentaphyllum* to identify genes and enzymes involved in triterpenoid biosynthesis. *Int J Genomics*. 2016;2016:1–10. 10.1155/2016/7840914 PMC5206855 28097124

[13] Choi KT . Botanical characteristics, pharmacological effects and medicinal components of korean Panax ginseng C. a Meyer. *Acta Pharmacol Sin*. 2008;29:1109–18. 18718180 10.1111/j.1745-7254.2008.00869.x

[14] Liang T , Zou L, Sun S et al. Hybrid sequencing of the *Gynostemma pentaphyllum* transcriptome provides new insights into gypenoside biosynthesis. *BMC Genomics*. 2019;20:632. 31382891 10.1186/s12864-019-6000-y PMC6683540

[15] Fan DD , Kuang YH, Xiang SX et al. Research progress in chemical constituents and pharmacological activities of *Gynostemma pentaphyllum*. *Chinese Pharmaceutical Journal*. 2017;52:342–52.

[16] Li ZH , Liu ZL, Zhao P et al. A review on studies of systematic evolution of *Gynostemma* Bl. *Acta Botan Boreali-Occiden Sin*. 2012;32:2133–8.

[17] Wang C , Zhang H, Qian ZQ et al. Genetic differentiation in endangered *Gynostemma pentaphyllum* (Thunb.) Makino based on ISSR polymorphism and its implications for conservation. *Biochem Syst Ecol*. 2008;36:699–705.

[18] Pang M , Zou FP, Xiao YP. Construction of DNA finger print for *Gynostemma pentaphyllum* (Thunb) Makino based on RAPD analysis. *Journal of Shaanxi Normal University (Natural Science Edition)*. 2006;3:025.

[19] Zhang X , Su HL, Yang J et al. Population genetic structure, migration, and polyploidy origin of a medicinal species *Gynostemma pentaphyllum* (cucurbitaceae). *Ecology and Evolution*. 2019;9:11145–70. 31641462 10.1002/ece3.5618 PMC6802062

[20] Jiang LY , Qian ZQ, Guo ZG et al. Polyploid origins in *Gynostemma pentaphyllum* (Cucurbitaceae) inferred from multiple gene sequences. *Mol Phylogenet Evol*. 2009;52:183–91. 19292995 10.1016/j.ympev.2009.03.004

[21] Zhang LS , Chen F, Zhang X et al. The water lily genome and the early evolution of flowering plants. *Nature*. 2020;577:1–6. 10.1038/s41586-019-1852-5 PMC7015852 31853069

[22] Xie D , Xu Y, Wang J et al. The wax gourd genomes offer insights into the genetic diversity and ancestral cucurbit karyotype. *Nat Commun*. 2019;10:5158. 31727887 10.1038/s41467-019-13185-3 PMC6856369

[23] Wei Q , Wang J, Wang W et al. A high-quality chromosome-level genome assembly reveals genetics for important traits in eggplant. *HorticRes*. 2020;7:153. 10.1038/s41438-020-00391-0 PMC7506008 33024567

[24] Shen C , du H, Chen Z et al. The chromosome-level genome sequence of the autotetraploid alfalfa and resequencing of core germplasms provide genomic resources for alfalfa research. *Mol Plant*. 2020;13:1250–61. 32673760 10.1016/j.molp.2020.07.003

[25] Cheng J , Wang X, Liu X et al. Chromosome-level genome of himalayan yew provides insights into the origin and evolution of the paclitaxel biosynthetic pathway. *Mol Plant*. 2021;14:1199–209. 33951484 10.1016/j.molp.2021.04.015

[26] Rennera SS , Wu S, Pérez-Escobarc OA et al. A chromosome-level genome of a Kordofan melon illuminates the origin of domesticated watermelons. *Proc Natl Acad Sci U S A*. 2021;118:1–9. 10.1073/pnas.2101486118 PMC8201767 34031154

[27] Kumar S , Kirk C, Deng C et al. Genotyping - by - sequencing of pear (Pyrus spp.). Accessions unravel novel patterns of genetic diversity and selection footprints. *Hortic Res*. 2017;4:17015. 28451438 10.1038/hortres.2017.15 PMC5389204

[28] Munyengwa N , Guen VL, Bille HN et al. Optimizing imputation of marker data from genotyping - by - sequencing (GBS). For genomic selection in non - model species: rubber tree (*Hevea brasiliensis*) as a case study. *Genomics*. 2021;113:655–68. 33508443 10.1016/j.ygeno.2021.01.012

[29] Nankar AN , Pratt RC. Genotyping by sequencing reveals genetic relatedness of southwestern u. s. Blue maize landraces. *Int J Mol Sci*. 2021;22:3436. 33810494 10.3390/ijms22073436 PMC8037273

[30] Simao FA , Waterhouse RM, Ioannidis P et al. BUSCO: assessing genome assembly and annotation completeness with single-copy orthologs. *Bioinformatics*. 2015;31:3210–2. 26059717 10.1093/bioinformatics/btv351

[31] Zhang X , Zhou T, Yang J et al. Comparative analyses of chloroplast genomes of Cucurbitaceae species: lights into selective pressures and phylogenetic relationships. *Molecules*. 2018;23:2165. 30154353 10.3390/molecules23092165 PMC6225112

[32] Ma LL , Wang Q, Zheng Y et al. Cucurbitaceae genome evolution, gene function and molecular breeding. *Hortic Res*. 2022;9:uhab057. 35043161 10.1093/hr/uhab057 PMC8969062

[33] Guo J , Xu W, Hu Y et al. Phylotranscriptomics in Cucurbitaceae reveal multiple whole - genome duplications and key morphological and molecular innovations. *Mol Plant*. 2020;13:1117–33. 32445889 10.1016/j.molp.2020.05.011

[34] Huang D , Ming R, Xu S et al. Chromosome-level genome assembly of *Gynostemma pentaphyllum* provides insights into gypenoside biosynthesis. *DNA Res*. 2021;28:5. 10.1093/dnares/dsab018 PMC8476931 34499150

[35] Fu AZ , Wang Q, Mu J et al. Combined genomic, transcriptomic, and metabolomic analyses provide insights into chayote (S*echium edule*) evolution and fruit development. *Hortic Res*. 2021;8:15. 33517348 10.1038/s41438-021-00487-1 PMC7847470

[36] Wu S , Shamimuzzaman M, Sun H et al. The bottle gourd genome provides insights into Cucurbitaceae evolution and facilitates mapping of a *papaya ring-spot virus* resistance locus. *Plant J*. 2017;92:963–75. 28940759 10.1111/tpj.13722

[37] Sun HE , Wu S, Zhang G et al. Karyotype stability and unbiased fractionation in the paleo-allotetraploid *Cucurbita* genomes. *Mol Plant*. 2018;10:1293–306. 10.1016/j.molp.2017.09.003 28917590

[38] Guo S , Zhao S, Sun H et al. Resequencing of 414 cultivated and wild watermelon accessions identifies selection for fruit quality traits. *Nat Genet*. 2019;51:1616–23. 31676863 10.1038/s41588-019-0518-4

[39] Ma LL , Wang Q, Mu J et al. The genome and transcriptome analysis of snake gourd provide insights into its evolution and fruit development and ripening. *Hortic Res*. 2020;7:199. 33328440 10.1038/s41438-020-00423-9 PMC7704671

[40] Guo YL . Gene family evolution in green plants with emphasis on the origination and evolution of *Arabidopsis thaliana* genes. *Plant J*. 2013;73:941–51. 23216999 10.1111/tpj.12089

[41] Chen JY , Xie FF, Cui YZ et al. A chromosome-scale genome sequence of pitaya (Hylocereus undatus). Provides novel insights into the genome evolution and regulation of betalain biosynthesis. *Hortic Res*. 2021;8:164–9. 34230458 10.1038/s41438-021-00612-0 PMC8260669

[42] Zhou XJ , Li JT, Wang HL et al. The chromosome-scale genome assembly, annotation and evolution of *rhododendron henanense* subsp. *lingbaoense*. *Mol Ecol Resour*. 2021;00:1–14. 10.1111/1755-0998.13529 34652864

[43] Hohmann N , Wolf EM, Lysak MA et al. A time-calibrated road map of brassicaceae species radiation and evolutionary history. *Plant Cell*. 2015;27:2770–84. 26410304 10.1105/tpc.15.00482 PMC4682323

[44] Wang JP , Sun P, Li Y et al. An overlooked paleotetraploidization in Cucurbitaceae. *Mol Biol Evol*. 2018;35:16–26. 29029269 10.1093/molbev/msx242 PMC5850751

[45] Barrera-Redondo J , Lira R, Eguiarte LE. Gourds and tendrils of Cucurbitaceae: how their shape diversity, molecular and morphological novelties evolved via whole-genome duplications. *Mol Plant*. 2020;13:1108–10. 32622159 10.1016/j.molp.2020.06.012

[46] Chen SK . A classificatory system and geographical distribution of the genus *Gynostemma* BL. (Cucurbitaceae). *Acta Phytotaxonomica Sinica*. 1995;33:403–10.

[47] Morris AB , Ickert-Bond SM, Brunson DB et al. Phylogeographical structure and temporal complexity in American sweetgum (*Liquidambar styraciflua.* Altingiaceae). *Mol Ecol*. 2008;17:3889–900. 18662227 10.1111/j.1365-294X.2008.03875.x

[48] Gavin DG , Fitzpatrick MC, Gugger PF et al. Climate refugia: joint inference from fossil records, species distribution models and phylogeography. *New Phytol*. 2015;204:37–54. 10.1111/nph.12929 25039238

[49] Wang YH , Jiang WM, Comes HP et al. Molecular phylogeography and ecological niche modelling of a widespread herbaceous climber, *tetrastigma hemsleyanum* (Vitaceae): insights into plio-pleistocene range dynamics of evergreen forest in subtropical China. New Phytol. 2015;206:852–67. 25639152 10.1111/nph.13261

[50] Mellick R , Lowe AJ, Allen CB et al. Palaeodistribution modelling and genetic evidence highlight differential post - glacial range shifts of a rain forest conifer distributed across a latitudinal gradient. *J Biogeogr*. 2012;39:2292–302.

[51] Shi YF , Li JJ, Li BY et al. Uplift of the Qinghai-xizang (tibetan) plateau and east asia environmental change during late Cenozoic. *Acta Geograph Sin*. 1999;54:10–20.

[52] COHMAP Project Members Climatic changes of the last 18000 years: observations and model simulation. *Science*. 1988;241:1043–52. 17747487 10.1126/science.241.4869.1043

[53] Peltier WR . Ice age palaeotopography. *Science*. 1994;265:195–201. 17750657 10.1126/science.265.5169.195

[54] Jiang D , Wang H, Lang X. Palaeoclimate modeling for the lgm and the possible influence of the continental ice sheet over the Qinghai-xizang plateau. *Quaternary Sciences*. 2002;22:323–31.

[55] Ye Q , Glantz MH. The 1998 Yangtze floods: the use of short-term forecasts in the context of seasonal to interannual water resource management. *Mitig Adapt Strateg Glob Chang*. 2005;10:159–82.

[56] Chen XD , Yang J, Guo YF et al. Spatial genetic structure and demographic history of the dominant Forest oak *Quercus fabri* Hance in subtropical China. *Front Plant Sci*. 2021;11:1–14. 10.3389/fpls.2020.583284 PMC7889815 33613578

[57] Liu J , Möller M, Provan J et al. Geological and ecological factors drive cryptic speciation of yews in a biodiversity hotspot. *New Phytol*. 2013;199:1093–108. 23718262 10.1111/nph.12336

[58] Zhang Z , Li PJ, Yang Y. The summarize of *Gynostemma pentaphyllum* (thumb.) Makino. *Food Res Dev*. 2011;32:193–6.

[59] Chen YX , Chen Y, Shi C et al. SOAPnuke: a MapReduce acceleration - supported software for integrated quality control and preprocessing of high - throughput sequencing data. *Gigascience*. 2018;7:1–6. 10.1093/gigascience/gix120 PMC5788068 29220494

[60] Chikhi R , Medvedev P. Informed and automated k-mer size selection for genome assembly. *Bioinformatics*. 2013;30:31–7. 23732276 10.1093/bioinformatics/btt310

[61] Marçais G , Kingsford C. A fast, lock-free approach for efficient parallel counting of occurrences of k-mers. *Bioinformatics*. 2011;27:764–70. 21217122 10.1093/bioinformatics/btr011 PMC3051319

[62] Vurture GW , Sedlazeck FJ, Nattestad M et al. Genomescope: fast reference-free genome profiling from short reads. *Bioinformatics*. 2017;33:2202–4. 28369201 10.1093/bioinformatics/btx153 PMC5870704

[63] Chen Y , Nie F, Xie SQ et al. Fast and accurate assembly of Nanopore reads via progressive error correction and adaptive read selection. *bioRxiv*. 2020.

[64] Vaser R , Sović I, Nagarajan N et al. Fast and accurate de novo genome assembly from long uncorrected reads. *Genome Res*. 2017;27:737–46. 28100585 10.1101/gr.214270.116 PMC5411768

[65] Walker BJ , Abeel T, Shea T et al. Pilon: an integrated tool for comprehensive microbial variant detection and genome assembly improvement. *PLoS One*. 2014;9:e112963. 25409509 10.1371/journal.pone.0112963 PMC4237348

[66] Roach MJ , Schmidt SA, Borneman AR. Purge Haplotigs: allelic contig reassignment for third-gen diploid genome assemblies. *BMC Bioinformatics*. 2018;19:460. 30497373 10.1186/s12859-018-2485-7 PMC6267036

[67] Li H . Aligning sequence reads, clone sequences and assembly contigs with BWA - MEM. 2013; arXiv: 1303. 3997.

[68] Li H , Handsaker B, Wysoker A et al. The sequence alignment/map format and samtools. *Bioinformatics*. 2009;25:2078–9. 19505943 10.1093/bioinformatics/btp352 PMC2723002

[69] Durand NC , Shamim MS, Machol I et al. Juicer provides a one-click system for Analyzing loop-resolution hi-C experiments. *Cell Systems*. 2016;3:95–8. 27467249 10.1016/j.cels.2016.07.002 PMC5846465

[70] Dudchenko O , Batra SS, Omer AD et al. De novo assembly of the *Aedes aegypti* genome using hi-C yields chromosome-length scaffolds. *Science*. 2017;356:92–5. 28336562 10.1126/science.aal3327 PMC5635820

[71] Smit AFA , Hubley R. RepeatModeler open - 1. 0. 2008; available at www.repeatmasker.org.

[72] Jurka J , Kapitonov VV, Pavlicek A et al. Repbase update, a database of eukaryotic repetitive elements. *Cytogenetic and Genome Research*. 2005;110:462–7. 16093699 10.1159/000084979

[73] Price AL , Jones NC, Pevzner PA. De novo identification of repeat families in large genomes. Bioinformatics. 2005;21:i351–8. 15961478 10.1093/bioinformatics/bti1018

[74] Edgar RC , Myers EW. PILER: identification and classification of genomic repeats. *Bioinformatics*. 2005;21:i152–8. 15961452 10.1093/bioinformatics/bti1003

[75] Xu Z , Wang H. LTR_FINDER: an efficient tool for the prediction of full - length LTR retrotransposons. *Nucleic Acids Res*. 2007;36:W265–8. 10.1093/nar/gkm286 PMC1933203 17485477

[76] Benson G . Tandem repeats finder: a program to analyze DNA sequences. *Nucleic Acids Res*. 1999;27:573–80. 9862982 10.1093/nar/27.2.573 PMC148217

[77] Zhao X , Hao W. LTR_FINDER: an efficient tool for the prediction of full-length LTR retrotransposons. *Nucleic Acids Res*. 2007;35:W265–8. 17485477 10.1093/nar/gkm286 PMC1933203

[78] Cantarel BL , Korf I, Robb SMC et al. MAKER: an easy - to - use annotation pipeline designed for emerging model organism genomes. *Genome Res*. 2008;18:188–96. 18025269 10.1101/gr.6743907 PMC2134774

[79] Altschul S , Gish W, Miller W et al. Basic local alignment search tool. *J Mol Biol*. 1990;215:403–10. 2231712 10.1016/S0022-2836(05)80360-2

[80] Emms DM , Kelly S. Orthofinder: solving fundamental biases in whole genome comparisons dramatically improves orthogroup inference accuracy. *Genome Biol*. 2015;16:157. 26243257 10.1186/s13059-015-0721-2 PMC4531804

[81] Katoh K , Misawa K, Kuma K et al. Mafft: a novel method for rapid multiple sequence alignment based on fast fourier transform. *Nucleic Acids Res*. 2002;30:3059–66. 12136088 10.1093/nar/gkf436 PMC135756

[82] John P , Huelsenbeck F, Ronquist. Mrbayes: bayesian inference of phylogenetic trees. *Bioinformatics*. 2001;17:754–5. 11524383 10.1093/bioinformatics/17.8.754

[83] Guindon S , Delsuc F, Dufayard JF et al. Estimating maximum likelihood phylogenies with phyml. *Methods Mol Biol*. 2009;537:113–37. 19378142 10.1007/978-1-59745-251-9_6

[84] Yang Z . Paml: a program package for phylogenetic analysis by maximum likelihood. *Computer Applications in the Biosciences Cabios*. 1997;13:555–6. 9367129 10.1093/bioinformatics/13.5.555

[85] Sudhir K , Glen S, Michael S et al. Timetree: a resource for timelines, timetrees, and divergence times. *Molecular Biology & Evolution*. 2017;7:1812. 10.1093/molbev/msx116 28387841

[86] Wang Y , Tang H, DeBarry JD et al. Mcscanx: a toolkit for detection and evolutionary analysis of gene synteny and collinearity. *Nucleic Acids Res*. 2012;40:e49–9. 22217600 10.1093/nar/gkr1293 PMC3326336

[87] Wu S , Wang X, Reddy U et al. Genome of 'charleston gray', the principal american watermelon cultivar, and genetic characterization of 1365 accessions in the u. s. National plant germplasm system watermelon collection. *Plant Biotechnol J*. 2019;17:2246–58. 31022325 10.1111/pbi.13136 PMC6835170

[88] Montero-Pau J , José B, Bombarely A et al. De-novo assembly of zucchini genome reveals a whole genome duplication associated with the origin of the cucurbita genus. *Plant Biotechnol J*. 2018;16:1161–71. 29112324 10.1111/pbi.12860 PMC5978595

[89] Wóycicki R , Witkowicz J, Gawroński P et al. The genome sequence of the north-european cucumber (*Cucumis sativus* l.) unravels evolutionary adaptation mechanisms in plants. *PLoS One*. 2011;6:e22728. 21829493 10.1371/journal.pone.0022728 PMC3145757

[90] Griesmann M , Chang Y, Liu X et al. Phylogenomics reveals multiple losses of nitrogen-fixing root nodule symbiosis. *Science*. 2018;361:1743. 10.1126/science.aat1743 29794220

[91] Sun, P., Jiao, B., Yang, Y., Shan, L., & Liu, J. (2021). WGDI: A User - Friendly Toolkit for Evolutionary Analyses of Whole-Genome Duplications and Ancestral Karyotypes. *bioRxiv. 2021*. 10.1016/j.molp.2022.10.018 36307977

[92] Badouin H , Gouzy J, Grassa CJ et al. The sunflower genome provides insights into oil metabolism, flowering and Asterid evolution. *Nature*. 2017;546:148–52. 28538728 10.1038/nature22380

[93] Bie TD , Cristianini N, Demuth JP et al. Cafe: a computational tool for the study of gene family evolution. *Bioinformatics*. 2006;22:1269–71. 16543274 10.1093/bioinformatics/btl097

[94] Kim D , Langmead B, Salzberg S. HISAT: a fast spliced aligner with low memory requirements. *Nat Methods*. 2015;12:357–60. 25751142 10.1038/nmeth.3317 PMC4655817

[95] Pertea M , Pertea GM, Antonescu CM et al. StringTie enables improved reconstruction of a transcriptome from RNA - seq reads. *Nat Biotechnol*. 2015;33:290–5. 25690850 10.1038/nbt.3122 PMC4643835

[96] Trapnell C , Williams BA, Pertea G et al. Transcript assembly and quantification by RNA-seq reveals unannotated transcripts and isoform switching during cell differentiation. *Nat Biotechnol*. 2010;28:511–5. 20436464 10.1038/nbt.1621 PMC3146043

[97] Chen C , Chen H, Zhang Y et al. Tbtools: an integrative toolkit developed for interactive analyses of big biological data. *Mol Plant*. 2020;13:1194–202. 32585190 10.1016/j.molp.2020.06.009

[98] Wong MM , Gujaria-Verma N, Ramsay L et al. Classification and characterization of species within the genus *lens* using genotyping - by - sequencing (GBS). *PLoS One*. 2015;10:e0122025. 25815480 10.1371/journal.pone.0122025 PMC4376907

[99] McKenna A , Hanna M, Banks E et al. The genome analysis toolkit: a mapreduce framework for analyzing next - generation dna sequencing data. *Genome Res*. 2010;20:1297–303. 20644199 10.1101/gr.107524.110 PMC2928508

[100] Danecek P , Auton A, Abecasis G et al. The variant call format and VCFtools. *Bioinformatics*. 2011;27:2156–8. 21653522 10.1093/bioinformatics/btr330 PMC3137218

[101] Purcell S , Neale B, Todd-Brown K et al. PLINK: a tool set for whole-genome association and population-based linkage analyses. *Am J Hum Genet*. 2007;81:559–75. 17701901 10.1086/519795 PMC1950838

[102] Stamatakis A . Raxml-vi-hpc: maximum likelihood-based phylogenetic analyses with thousands of taxa and mixed models. *Bioinformatics*. 2006;22:2688–90. 16928733 10.1093/bioinformatics/btl446

[103] Yang J , Lee SH, Goddard ME et al. GCTA: a tool for genome-wide complex trait analysis. *Am J Hum Genet*. 2011;88:76–82. 21167468 10.1016/j.ajhg.2010.11.011 PMC3014363

[104] Excoffier L , Lischer HEL. Arlequin suite ver 3.5: a new series of programs to perform population genetics analyses under Linux and windows. *Mol Ecol Resour*. 2010;10:564–7. 21565059 10.1111/j.1755-0998.2010.02847.x

[105] Terhorst J , Kamm JA, Song YS. Robust and scalable inference of population history from hundreds of unphased whole genomes. *Nat Genet*. 2017;49:303–9. 28024154 10.1038/ng.3748 PMC5470542

[106] Li H , Durbin R. Inference of human population history from individual whole-genome sequences. *Nature*. 2011;475:493–6. 21753753 10.1038/nature10231 PMC3154645

[107] Koch MA , Bernhard H, Thomas MO. Comparative evolutionary analysis of chalcone synthase and alcohol dehydrogenase loci in *Arabidopsis*, *Arabis*, and related genera (Brassicaceae). *Molecular Biology & Evolution*. 2000;10:1483. 10.1093/oxfordjournals.molbev.a026248 11018155

[108] Zhang L , Yan HF, Wu W et al. Comparative transcriptome analysis and marker development of two closely related primrose species (*Primula poissonii* and *Primula wilsonii*). *BMC Genomics*. 2013;14:329–9. 23672467 10.1186/1471-2164-14-329 PMC3658987

[109] Rehman HM , Nawaz MA, Shah ZH et al. In-depth genomic and transcriptomic analysis of five k+ transporter gene families in soybean confirm their differential expression for nodulation. *Front Plant Sci*. 2017;8:1–14. 28588592 10.3389/fpls.2017.00804 PMC5440519

[110] Petitpierre B , Kueffer C, Broennimann O et al. Climatic niche shifts are rare among terrestrial plant invaders. *Science*. 2012;335:1344–8. 22422981 10.1126/science.1215933

[111] Chen XD , Zhang X, Zhang H et al. Genetic differentiation and demographic history of three Cerris oak species in China based on nuclear microsatellite makers. *Forests*. 2021;12:1164.

[112] Phillips SJ , Anderson RP, Schapire RE. Maximum entropy modeling of species geographic distributions. *Ecol Model*. 2006;190:231–59.

[113] Feng L , Zheng QJ, Qian ZQ et al. Genetic structure and evolutionary history of three Alpine Sclerophyllous oaks in east Himalaya-Hengduan Mountains and adjacent regions. *Front Plant Sci*. 2016;7:1688. 27891142 10.3389/fpls.2016.01688 PMC5104984

[114] Broennimann O , Fitzpatrick MC, Pearman PB et al. Measuring ecological niche overlap from occurrence and spatial environmental data. *Glob Ecol Biogeogr*. 2012;21:481–97.

[115] Jia Y , Milne RI, Zhu J et al. Evolutionary legacy of a forest plantation tree species (*Pinus armandii*): implications for widespread afforestation. *Evol Appl*. 2020;00:1–17. 10.1111/eva.13064 PMC7691453 33294014

[116] Silva DP , Vilela B, Buzatto BA et al. Contextualized niche shifts upon independent invasions by the dung beetle *Onthophagus taurus*. *Biol Invasions*. 2016;18:3137–48.

