# Supplementary material for: Diploid chromosome-level reference genome and population genomic analyses provide insights into Gypenoside biosynthesis and demographic evolution of Gynostemma pentaphyllum (Cucurbitaceae)
Source: Hortic Res. 2022 Oct 19;10(1):uhac231. doi: 10.1093/hr/uhac231 (PMC9832869; doi:10.1093/hr/uhac231)
Supplement: Web_Material_uhac231 [file web_material_uhac231.zip › Notes.docx]

**Note S1. Details of gene structure prediction.**

For de novo prediction, AUGUSTUS v 3.3.1^1^ and SNAP v2006-07-28 were used to predict the coding regions of genes after masking the repeat sequences. For homolog-based prediction, Blast v2.2.31 and Exonerate v2.2.0^2^ were used to map the protein repertoires of *Cucumis melo, Citrullus lanatus, Cucumis sativus, Benincasa hispida, Momordica charantia, Cucurbita pepo, Lagenaria siceraria, Cucurbita moschata, Cucurbita maxima* to the *G. pentaphyllum* genome with an E-value cutoff of 1e-5 to obtain rough approximate alignments, respectively. Transcriptomic data sequenced from *G. pentaphyllum* RNA samples were mapped to the obtained *G. pentaphyllum* genome sequence using HISAT2 v. 2.1.0 to search candidate open reading frames. Then StringTie v1.3.4d software was run using the candidate result to complete the alignment assembly, generate a comprehensive transcriptome dataset. Finally, Maker v 2.31.8 was also used to integrated a non-overlapping gene sets from the polished and clustered de novo, EST and protein alignments.

**Note S2. Methods of population genetic diversity and structure.**

Population genetic parameters including observed heterozygosity (*Ho*), expected heterozygosity (*He*) and F-index (F) of each population were obtained based on PLINK1.7 software. Population structure was investigated using ADMIXTURE program^3^ using the maximum likelihood-based clustering algorithm. A series of tests were conducted while the value of K was assumed from 1 to 8, and accessions were assigned to a corresponding population based on their maximum membership probabilities. The optimal clustering number was confirmed according to the valley value of cross-validation (CV) errors. Then, the plots were visualized using Distruct v1.1^4^, and the ArcGIS (Esri) program was performed to draw the distribution map.

**Note S3. Details of six climatic variables.**

Bio2, Mean monthly temperature range

Bio4, Temperature seasonality (STD*100)

Bio9, Mean temperature of driest quarter

Bio14, Precipitation of driest month

Bio15, Precipitation seasonality (CV)

Bio18, Precipitation of warmest quarter

**References**

1 Schaefer, H., Heibl, C., & Renner, S. S. (2008). Gourds afloat: a dated phylogeny reveals an asian origin of the gourd family (Cucurbitaceae). and numerous oversea dispersal events. Proceedings of the Royal Society B: Biological Sciences. 276 (1658): 843 - 851.

2 Slater, G. S. C. & Birney, E. (2005). Automated generation of heuristics for biological sequence comparison. BMC Bioinformatics. 6: 1471 - 2105.

3 Alexander, D. H., Novembre, J., & Lange, K., (2009). Fast model - based estimation of ancestry in unrelated individuals. Genome research. 19 (9): 1655 - 1664.

4 Rosenberg, N. A. (2004). Distruct: a program for the graphical display of population structure. Molecular Ecology Notes. 4: 137 - 138.
