# Supplementary material for: Diploid chromosome-level reference genome and population genomic analyses provide insights into Gypenoside biosynthesis and demographic evolution of Gynostemma pentaphyllum (Cucurbitaceae)
Source: Hortic Res. 2022 Oct 19;10(1):uhac231. doi: 10.1093/hr/uhac231 (PMC9832869; doi:10.1093/hr/uhac231)
Supplement: Web_Material_uhac231 [file web_material_uhac231.zip › Table S.R2.docx]

**Table S1 Statistics of the K-mer analyses.**

| **K** | **K-mer Number** | **peak** | **Genome Size (bp)** | Repeat（%） | **Heterozygous Ratio (%)** | **Sequence Depth (X)** |
| --- | --- | --- | --- | --- | --- | --- |
| 17 | 71,434,528,634 | 120 | 592,287,117 | 72.16 | 1.55 | 131 |
| 31 | 63,957,533,280 | 101 | 591,096,096 | 66.4 | 1.49 | 133 |

**Table S2. Statistics of the assembly results.**

|  | **contig** |  | **Super-scaffold** |  |
| --- | --- | --- | --- | --- |
|  | **length(bp)** | **number** | **length(bp)** | **number** |
| max_len | 13,104,745 |  | 68,721,639 |  |
| N10 | 9,702,875 | 6 | 68,721,639 | 1 |
| N20 | 7,800,363 | 13 | 62,363,120 | 2 |
| N30 | 6,371,657 | 22 | 60,991,533 | 3 |
| N40 | 5,588,332 | 32 | 57,792,157 | 4 |
| N50 | 5,045,119 | 43 | 56,377,829 | 5 |
| N60 | 4,397,350 | 56 | 54,713,503 | 7 |
| N70 | 3,993,454 | 70 | 50,498,967 | 8 |
| N80 | 3,082,015 | 88 | 49,856,026 | 9 |
| N90 | 2,549,506 | 109 | 47,314,670 | 10 |
| Total length (number>=2000bp) | 608,916,630 | 158 | 608,986,630 | 18 |
| chromosome scale scaffold length |  |  | 608,952,153 | 11 |
| GC_rate(%) | 32.95 |  | 32.95 |  |
| Hi-C Anchored rate(%) | 608,882,153 |  | 99.99 |  |

**Table S3 Statistics of the chromosome length.**

| **ID** | **lengh** | **N%** | **GC%** | **gap** |
| --- | --- | --- | --- | --- |
| Chr1 | 55,162,749 | 0.01 | 33.05 | 13 |
| Chr2 | 50,498,967 | 0.01 | 32.94 | 13 |
| Chr3 | 49,856,026 | 0.01 | 33.07 | 9 |
| Chr4 | 47,314,670 | 0.01 | 33.33 | 8 |
| Chr5 | 45,159,960 | 0.01 | 32.79 | 11 |
| Chr6 | 56,377,829 | 0.01 | 32.68 | 10 |
| Chr7 | 60,991,533 | 0.01 | 32.80 | 16 |
| Chr8 | 62,363,120 | 0.01 | 32.96 | 15 |
| Chr9 | 57,792,157 | 0.01 | 32.98 | 12 |
| Chr10 | 54,713,503 | 0.01 | 33.07 | 16 |
| Chr11 | 68,721,639 | 0.01 | 32.75 | 17 |
|  |  |  |  |  |
| Quality | Average_len | total_N% | total_GC% | Gap_circle_rate% |
|  | 55,359,287 | 0.01 | 32.95 | 61.11 |

**Table S4. Statistics of NGS sequencing reads mapped back to the assembly G. pentaphyllum genome.**

|  | **Number** | **Ratio (%)** |
| --- | --- | --- |
| Total Reads | 694,962,560 |  |
| Mapped Reads | 689,524,974 | 99.22 |
| Mapped and Paired Reads | 612,149,166 | 90.25 |

**Table S5 Gene model prediction in G. pentaphyllum genome.**

| **Strategies** | **Gene set** | **Number** | **Average mRNA length (bp)** | **Average CDS length (bp)** | **Average exon per gene** | **Average exon length (bp)** | **Average intron length (bp)** |
| --- | --- | --- | --- | --- | --- | --- | --- |
| **De novo** | Augustus | 67,323 | 1,661 | 760 | 4 | 208 | 339 |
|  | SNAP | 96,015 | 886 | 514 | 3 | 194 | 226 |
| **Homolog** | *Cucumis melo* | 70,369 | 13,535 | 1,158 | 5 | 233 | 3,115 |
|  | *Cucurbita pepo* | 104,733 | 12,346 | 1,158 | 5 | 231 | 2,781 |
|  | *Citrullus lanatus* | 49,446 | 13,009 | 1,080 | 4 | 241 | 3,427 |
|  | *Lagenaria siceraria* | 48,143 | 14,756 | 1,025 | 4 | 257 | 4,591 |
|  | *Cucumis sativus* | 73,275 | 14,267 | 1,186 | 5 | 237 | 3,261 |
|  | *Cucurbita moschata* | 103,985 | 12,466 | 1,180 | 5 | 230 | 2,729 |
|  | *Benincasa hispida* | 55,331 | 12,443 | 994 | 4 | 242 | 3,692 |
|  | *Cucurbita maxima* | 100,842 | 11,342 | 1,171 | 5 | 228 | 2,459 |
|  | *Momordica charantia* | 70,568 | 13,242 | 1,173 | 5 | 237 | 3,060 |
| **RNA-Seq** |  | 86,445 | 1,305 | 314 | 2 | 166 | 1,110 |
| **Maker** |  | 27,418 | 3,397 | 2,350 | 10 | 233 | 115 |

**Table S6. Annotated gene sets evaluation in BUSCO.**

| **Type** | **Number** | **Percentage** |
| --- | --- | --- |
| Complete BUSCOs (C) | 1258 | 91.50% |
| Complete and single-copy BUSCOs (S) | 1112 | 80.90% |
| Complete and duplicated BUSCOs (D) | 146 | 10.60% |
| Fragmented BUSCOs (F) | 62 | 4.50% |
| Missing BUSCOs (M) | 55 | 4.00% |
| Total BUSCO groups searched | 1375 |  |

**Table S7. Summary of gene function annotation**

| **Values** | **Number** | **Percentage** |
| --- | --- | --- |
| Total | 27,418 | 100% |
| Nr-Annotated | 26,387 | 96.24% |
| Nt-Annotated | 26,516 | 96.71% |
| Swissprot-Annotated | 21,538 | 78.55% |
| KEGG-Annotated | 21,053 | 76.79% |
| KOG-Annotated | 20,806 | 75.88% |
| TrEMBL-Annotated | 26,293 | 95.90% |
| Interpro-Annotated | 25,382 | 92.57% |
| GO-Annotated | 15,372 | 56.07% |
| Overall | 26,588 | 96.97% |

**Table S8. Statistics of non-coding annotated RNA.**

| **Type** |  | **Copy(w)** | **Average length(bp)** | **Total length(bp)** | **% of genome** |
| --- | --- | --- | --- | --- | --- |
| **miRNA** |  | **110** | **118.29** | **13,012** | **0.002137** |
| **tRNA** |  | **651** | **74.74** | **48,654** | **0.00799** |
| **rRNA** | **rRNA** | **1161** | **130.79** | **151,851** | **0.024938** |
|  | 18S | 62 | 796.58 | 49,388 | 0.008111 |
|  | 28S | 78 | 100.06 | 7,805 | 0.001282 |
|  | 5.8S | 27 | 111.89 | 3,021 | 0.000496 |
|  | 5S | 994 | 92.19 | 91,637 | 0.015049 |
| **snRNA** | **snRNA** | **459** | **114.96** | **52,767** | **0.008666** |
|  | CD-box | 310 | 103.06 | 31,949 | 0.005247 |
|  | HACA-box | 37 | 123.35 | 4,564 | 0.00075 |
|  | splicing | 112 | 145.13 | 16,254 | 0.002669 |

**Table S9. Information of species used in comparative genomic and evolutionary analyses.**

| **Species** | **Family** | **Genome size** （**Mb**） | **Number of chromosomes** | **NCBI ID** |
| --- | --- | --- | --- | --- |
| *Gynostemma pentaphyllum* | Cucurbitaceae | 608.95 | 11 |  |
| *Benincasa hispida* | Cucurbitaceae | 859 | 12 | 18245 |
| *Citrullus lanatus* | Cucurbitaceae | 367.91 | 11 | 10768 |
| *Cucurbita maxima* | Cucurbitaceae | 271.4 | scaffold | 13324 |
| *Cucumis melo* | Cucurbitaceae | 366.17 | 12 | 10697 |
| *Cucurbita moschata* | Cucurbitaceae | 269.9 | scaffold | 18321 |
| *Cucurbita pepo* | Cucurbitaceae | 263 | 20 | 12199 |
| *Cucumis sativus* | Cucurbitaceae | 226.2 | 7 | 1639 |
| *Lagenaria siceraria* | Cucurbitaceae | 313.4 | 11 | 10464 |
| *Momordica charantia* | Cucurbitaceae | 285.61 | scaffold | 12860 |
| *Datisca glomerata* | Datiscaceae | 688.4 | scaffold | 12838 |
| *Glycine max* | Fabaceae | 933.12 | 20 | 5 |

**Table S10. Summary of gene family clustering among 12 species.**

| **Species** | **Genes number** | **Genes in families** | **Unclustered genes** | **Family number** | **Unique families** | **Genes in Unique families** | **Average genes per family** |
| --- | --- | --- | --- | --- | --- | --- | --- |
| *Benincasa hispida* | 25,528 | 21,889 | 3,639 | 16,036 | 413 | 1,391 | 1.36 |
| *Citrullus lanatus* | 22,467 | 19,955 | 2,512 | 15,924 | 45 | 129 | 1.25 |
| *Cucurbita maxima* | 27,137 | 26,874 | 263 | 15,575 | 8 | 21 | 1.73 |
| *Cucurbita moschata* | 27,774 | 27,558 | 216 | 15,671 | 9 | 23 | 1.76 |
| *Cucurbita pepo* | 29,055 | 28,700 | 355 | 15,719 | 33 | 73 | 1.83 |
| *Cucumis melo* | 19,502 | 19,333 | 169 | 15,292 | 26 | 95 | 1.26 |
| *Cucumis sativus* | 19,958 | 19,858 | 100 | 15,304 | 28 | 217 | 1.30 |
| *Datisca glomerata* | 37,042 | 35,375 | 1,667 | 14,757 | 560 | 6,683 | 2.40 |
| *Glycine max* | 56,043 | 49,247 | 6,796 | 17,751 | 3,077 | 11,483 | 2.77 |
| *Gynostemma pentaphyllum* | 27,418 | 25,813 | 1,605 | 15,486 | 565 | 2,713 | 1.67 |
| *Lagenaria siceraria* | 22,472 | 20,177 | 2,295 | 15,543 | 59 | 141 | 1.30 |
| *Momordica charantia* | 18,958 | 18,719 | 239 | 14,644 | 26 | 127 | 1.28 |
| Total | 333,354 |  |  | 187,702 |  |  |  |

**Table S11. Comparation of Orthologues among 12 species.**

| **Species** | **Genes number** | **Single-copy genes** | **Multi-copy genes** | **Unique genes** | **Other genes** | **Unassigned genes** |
| --- | --- | --- | --- | --- | --- | --- |
| *Benincasa hispida* | 21,889 | 7,707 | 4,842 | 1,391 | 7,949 | 3,639 |
| *Citrullus lanatus* | 19,955 | 7,713 | 4,695 | 129 | 7,418 | 2,512 |
| *Cucurbita maxima* | 26,874 | 4,046 | 13,758 | 21 | 9,049 | 263 |
| *Cucurbita moschata* | 27,558 | 3,935 | 14,133 | 23 | 9,467 | 216 |
| *Cucurbita pepo* | 28,700 | 3,850 | 14,891 | 73 | 9,886 | 355 |
| *Cucumis melo* | 19,333 | 7,802 | 4,499 | 95 | 6,937 | 169 |
| *Cucumis sativus* | 19,858 | 7,742 | 4,782 | 217 | 7,117 | 100 |
| *Datisca glomerata* | 35,375 | 7,446 | 5,120 | 6,683 | 16,126 | 1,667 |
| *Glycine max* | 49,247 | 1,354 | 24,264 | 11,483 | 12,146 | 6,796 |
| *Gynostemma pentaphyllum* | 25,813 | 6,664 | 7,862 | 2,713 | 8,574 | 1,605 |
| *Lagenaria siceraria* | 20,177 | 7,738 | 4,771 | 141 | 7,527 | 2,295 |
| *Momordica charantia* | 18,719 | 7,930 | 4,067 | 127 | 6,595 | 239 |

**Table S12. KEGG pathway enrichment analysis of the expanded gene families in G. pentaphyllum.**

| **Pathway** | **DEGs with pathway annotation (2242)** | **All genes with pathway annotation (21053)** | **Pvalue** | **Qvalue** | **Pathway ID** | **Genes** | **KOs** |
| --- | --- | --- | --- | --- | --- | --- | --- |
| Plant-pathogen interaction | 416 (18.55%) | 954 (4.53%) | 4.08E-161 | 3.47E-159 | ko04626 | Gype01G0433.1;Gype02G0804.1;Gype02G0805.1;Gype04G1574.1;Gype05G0460.1;Gype05G0461.1;Gype05G0462.1;Gype05G0463.1;Gype05G0507.1;Gype05G0508.1;Gype05G0512.1;Gype06G0210.1;Gype06G0211.1;Gype07G2520.1;Gype07G2542.1;Gype07G2543.1;Gype07G2544.1;Gype07G2560.1;Gype07G2561.1;Gype07G2622.1;Gype07G2647.1;Gype07G2648.1;Gype07G2649.1;Gype07G2652.1;Gype07G2653.1;Gype07G2654.1;Gype07G2655.1;Gype07G2656.1;Gype07G2657.1;Gype07G2658.1;Gype07G2659.1;Gype07G2661.1;Gype07G2665.1;Gype07G2666.1;Gype07G2669.1;Gype07G2671.1;Gype07G2672.1;Gype07G2673.1;Gype07G2675.1;Gype07G2677.1;Gype07G2678.1;Gype07G2680.1;Gype07G2681.1;Gype07G2683.1;Gype07G2684.1;Gype07G2685.1;Gype07G2686.1;Gype07G2687.1;Gype07G2689.1;Gype07G2690.1;Gype07G2692.1;Gype07G2693.1;Gype07G2695.1;Gype07G2697.1;Gype07G2698.1;Gype07G2699.1;Gype07G2700.1;Gype07G2701.1;Gype07G2703.1;Gype07G2704.1;Gype07G2706.1;Gype07G2707.1;Gype07G2708.1;Gype07G2709.1;Gype07G2710.1;Gype07G2711.1;Gype07G2712.1;Gype07G2713.1;Gype07G2714.1;Gype07G2715.1;Gype07G2716.1;Gype07G2718.1;Gype07G2719.1;Gype07G2720.1;Gype07G2722.1;Gype07G2739.1;Gype07G2740.1;Gype07G2741.1;Gype07G2743.1;Gype07G2744.1;Gype07G2745.1;Gype07G2746.1;Gype07G2747.1;Gype07G2749.1;Gype07G2750.1;Gype07G2753.1;Gype07G2830.1;Gype07G2831.1;Gype07G2835.1;Gype07G2836.1;Gype07G2837.1;Gype07G2839.1;Gype07G2840.1;Gype07G2842.1;Gype07G2843.1;Gype07G2844.1;Gype07G2846.1;Gype07G2847.1;Gype07G2853.1;Gype07G2856.1;Gype07G2859.1;Gype07G2940.1;Gype07G2943.1;Gype07G3021.1;Gype07G3022.1;Gype07G3074.1;Gype07G3075.1;Gype07G3076.1;Gype07G3078.1;Gype07G3082.1;Gype07G3084.1;Gype07G3091.1;Gype07G3094.1;Gype07G3097.1;Gype07G3098.1;Gype07G3102.1;Gype07G3105.1;Gype07G3111.1;Gype07G3114.1;Gype07G3116.1;Gype07G3117.1;Gype07G3118.1;Gype07G3119.1;Gype07G3120.1;Gype08G0092.1;Gype09G1272.1;Gype09G1276.1;Gype09G1277.1;Gype09G1939.1;Gype09G1940.1;Gype01G1106.1;Gype01G1235.1;Gype05G1389.1;Gype05G1529.1;Gype05G1530.1;Gype05G1696.1;Gype05G1732.1;Gype05G1733.1;Gype05G1752.1;Gype05G1753.1;Gype05G1803.1;Gype05G1804.1;Gype05G1806.1;Gype05G1807.1;Gype05G1810.1;Gype05G1811.1;Gype05G1888.1;Gype05G1910.1;Gype05G1981.1;Gype05G2090.1;Gype05G2092.1;Gype05G2093.1;Gype06G2306.1;Gype06G3035.1;Gype08G0078.1;Gype08G0217.1;Gype08G1178.1;Gype08G2486.1;Gype08G2493.1;Gype08G2879.1;Gype09G1409.1;Gype02G0402.1;Gype02G0403.1;Gype02G0404.1;Gype02G0405.1;Gype02G0474.1;Gype02G0475.1;Gype04G0516.1;Gype05G0643.1;Gype05G0648.1;Gype05G0652.1;Gype05G0654.1;Gype05G0657.1;Gype05G0659.1;Gype05G0660.1;Gype05G0661.1;Gype05G0662.1;Gype05G0665.1;Gype05G0669.1;Gype05G0680.1;Gype05G0686.1;Gype05G0689.1;Gype05G0690.1;Gype05G0691.1;Gype05G0692.1;Gype05G0694.1;Gype05G0695.1;Gype05G0696.1;Gype05G0698.1;Gype05G0699.1;Gype05G0700.1;Gype05G0702.1;Gype05G0703.1;Gype05G0706.1;Gype05G0707.1;Gype05G0708.1;Gype05G0712.1;Gype05G0713.1;Gype05G0714.1;Gype05G0717.1;Gype05G0718.1;Gype05G0719.1;Gype05G0721.1;Gype05G0742.1;Gype05G0744.1;Gype05G0746.1;Gype05G0747.1;Gype05G0750.1;Gype05G0751.1;Gype05G0752.1;Gype05G0756.1;Gype05G0758.1;Gype05G0759.1;Gype05G0760.1;Gype05G0786.1;Gype05G0787.1;Gype05G0789.1;Gype05G0790.1;Gype05G0793.1;Gype05G0794.1;Gype05G0795.1;Gype05G0802.1;Gype05G0803.1;Gype05G0807.1;Gype05G0809.1;Gype05G0810.1;Gype05G0811.1;Gype05G0813.1;Gype05G0815.1;Gype05G0820.1;Gype05G0823.1;Gype01G0516.1;Gype01G0581.1;Gype01G0582.1;Gype01G0585.1;Gype09G0200.1;Gype10G1732.1;Gype10G1733.1;Gype10G1737.1;Gype10G1741.1;Gype10G1742.1;Gype10G1745.1;Gype10G1746.1;Gype10G1748.1;Gype10G1749.1;Gype10G1750.1;Gype10G1752.1;Gype10G1758.1;Gype01G0406.1;Gype01G0407.1;Gype04G0451.1;Gype04G0507.1;Gype04G0511.1;Gype04G0515.1;Gype04G0520.1;Gype04G0521.1;Gype04G0523.1;Gype04G0526.1;Gype04G0527.1;Gype04G0531.1;Gype04G0532.1;Gype04G0533.1;Gype04G0536.1;Gype04G0541.1;Gype04G0543.1;Gype04G0544.1;Gype04G0551.1;Gype04G0553.1;Gype04G0554.1;Gype04G0557.1;Gype04G0860.1;Gype04G1390.1;Gype09G1271.1;Gype10G1180.1;Gype10G1181.1;Gype10G0356.1;Gype10G0786.1;Gype10G0792.1;Gype02G0802.1;Gype05G0133.1;Gype05G0135.1;Gype05G0137.1;Gype05G0139.1;Gype05G0141.1;Gype05G0142.1;Gype05G0165.1;Gype05G0166.1;Gype05G0168.1;Gype06G0492.1;Gype06G0494.1;Gype08G0008.1;Gype08G0010.1;Gype09G1986.1;Gype09G1999.1;Gype09G2000.1;Gype09G2051.1;Gype11G0150.1;Gype11G0151.1;Gype11G0152.1;Gype11G2073.1;Gype08G0881.1;Gype02G1421.1;Gype11G0072.1;Gype11G0073.1;Gype11G0074.1;Gype11G0076.1;Gype11G0791.1;Gype11G0792.1;Gype11G0793.1;Gype11G0794.1;Gype11G0795.1;Gype02G1418.1;Gype02G1420.1;Gype02G1424.1;Gype02G1429.1;Gype05G0029.1;Gype09G1886.1;Gype09G1888.1;Gype10G0311.1;Gype04G0187.1;Gype11G0153.1;Gype11G0154.1;Gype11G0155.1;Gype11G0157.1;Gype11G0158.1;Gype11G1063.1;Gype11G1064.1;Gype11G1066.1;Gype11G1067.1;Gype11G1068.1;Gype11G1069.1;Gype11G1072.1;Gype11G1073.1;Gype11G1074.1;Gype11G1075.1;Gype07G2691.1;Gype05G0049.1;Gype05G0050.1;Gype05G0051.1;Gype05G0052.1;Gype05G0053.1;Gype05G0056.1;Gype05G0060.1;Gype05G0061.1;Gype10G1223.1;Gype11G2424.1;Gype11G2609.1;Gype11G2610.1;Gype11G2611.1;Gype11G2612.1;Gype11G2614.1;Gype11G2617.1;Gype11G2620.1;Gype11G2621.1;Gype11G2661.1;Gype11G2744.1;Gype11G2758.1;Gype09G1995.1;Gype05G0509.1;Gype05G0510.1;Gype06G0214.1;Gype07G2651.1;Gype07G2664.1;Gype07G2679.1;Gype07G2705.1;Gype07G3096.1;Gype07G3115.1;Gype04G0508.1;Gype04G0509.1;Gype04G0510.1;Gype04G0513.1;Gype04G0514.1;Gype04G0517.1;Gype04G0518.1;Gype04G0522.1;Gype04G0555.1;Gype04G0556.1;Gype10G1179.1;Gype09G1254.1;Gype09G1255.1;Gype09G1256.1;Gype09G1258.1;Gype09G1259.1;Gype09G1261.1;Gype09G1263.1;Gype09G1266.1;Gype09G1267.1;Gype09G1269.1;Gype09G1275.1;Gype11G2613.1;Gype03G2176.1;Gype03G2179.1;Gype03G2180.1;Gype03G2181.1;Gype03G2218.1;Gype03G2219.1;Gype03G2221.1;Gype03G2224.1;Gype05G0675.1;Gype06G0405.1;Gype06G0407.1;Gype06G0413.1;Gype06G0421.1;Gype06G0422.1;Gype06G0553.1;Gype06G0933.1;Gype07G1928.1;Gype08G2440.1;Gype11G2732.1;Gype07G2660.1;Gype07G2676.1;Gype07G2702.1;Gype07G2748.1;Gype07G3079.1;Gype02G1427.1;Gype02G1431.1 | K13459+K04079+K16224+K13420+K13466+K13465+K13428+K13416 |
| Tryptophan metabolism | 60 (2.68%) | 146 (0.69%) | 1.16E-21 | 4.94E-20 | ko00380 | Gype02G1525.1;Gype02G1527.1;Gype02G1528.1;Gype02G1529.1;Gype02G1530.1;Gype02G1531.1;Gype02G1534.1;Gype02G1535.1;Gype02G1536.1;Gype02G1537.1;Gype02G1538.1;Gype02G1539.1;Gype02G1540.1;Gype02G1543.1;Gype08G0011.1;Gype11G2798.1;Gype08G1056.1;Gype08G1057.1;Gype08G1058.1;Gype08G1060.1;Gype08G1062.1;Gype08G1066.1;Gype08G1068.1;Gype08G1072.1;Gype08G1073.1;Gype08G1075.1;Gype08G1077.1;Gype08G1080.1;Gype08G1084.1;Gype10G0826.1;Gype10G0501.1;Gype11G2189.1;Gype11G2191.1;Gype11G2193.1;Gype11G2196.1;Gype11G2199.1;Gype11G2200.1;Gype11G2320.1;Gype11G2322.1;Gype11G2324.1;Gype11G2325.1;Gype11G2327.1;Gype11G2328.1;Gype07G2427.1;Gype08G0400.1;Gype09G1032.1;Gype09G1971.1;Gype09G2007.1;Gype09G2019.1;Gype09G2052.1;Gype02G1526.1;Gype02G1532.1;Gype02G1533.1;Gype11G2190.1;Gype11G2192.1;Gype11G2194.1;Gype11G2198.1;Gype11G2321.1;Gype11G2323.1;Gype11G2326.1 | K11868+K01593+K01426 |
| Isoflavonoid biosynthesis | 47 (2.1%) | 95 (0.45%) | 2.26E-21 | 6.39E-20 | ko00943 | Gype09G0581.1;Gype09G0582.1;Gype09G0583.1;Gype09G0584.1;Gype09G0585.1;Gype09G0586.1;Gype09G0587.1;Gype09G0588.1;Gype09G0589.1;Gype09G0591.1;Gype09G0592.1;Gype09G0593.1;Gype09G0594.1;Gype09G0595.1;Gype09G0596.1;Gype09G0597.1;Gype09G0598.1;Gype09G2069.1;Gype09G2070.1;Gype09G2071.1;Gype09G2072.1;Gype11G2641.1;Gype11G2646.1;Gype11G2747.1;Gype11G2752.1;Gype11G2753.1;Gype11G2761.1;Gype11G2763.1;Gype11G2764.1;Gype11G2773.1;Gype11G2774.1;Gype11G2775.1;Gype11G2776.1;Gype11G2777.1;Gype11G2778.1;Gype11G2782.1;Gype02G1810.1;Gype05G1946.1;Gype05G1947.1;Gype05G1948.1;Gype05G1949.1;Gype05G1950.1;Gype05G1951.1;Gype09G2068.1;Gype11G2147.1;Gype11G2282.1;Gype11G2647.1 | K13260+K13264 |
| Flavone and flavonol biosynthesis | 41 (1.83%) | 88 (0.42%) | 1.28E-17 | 2.72E-16 | ko00944 | Gype04G0326.1;Gype04G0329.1;Gype04G0341.1;Gype04G0342.1;Gype04G0345.1;Gype05G1394.1;Gype05G1397.1;Gype05G1401.1;Gype05G1403.1;Gype05G1404.1;Gype05G1405.1;Gype05G1406.1;Gype05G1408.1;Gype11G2669.1;Gype11G2670.1;Gype11G2673.1;Gype11G2685.1;Gype11G2686.1;Gype11G2689.1;Gype11G2698.1;Gype11G2699.1;Gype04G0328.1;Gype04G0336.1;Gype04G0348.1;Gype05G1393.1;Gype05G1396.1;Gype09G1952.1;Gype09G2037.1;Gype11G2667.1;Gype11G2672.1;Gype11G2681.1;Gype11G2683.1;Gype11G2691.1;Gype11G2695.1;Gype02G1810.1;Gype05G1946.1;Gype05G1947.1;Gype05G1948.1;Gype05G1949.1;Gype05G1950.1;Gype05G1951.1 | K22771+K13264 |
| Carbon fixation in photosynthetic organisms | 53 (2.36%) | 163 (0.77%) | 3.32E-14 | 5.65E-13 | ko00710 | Gype01G0409.1;Gype01G0442.1;Gype02G0430.1;Gype04G0415.1;Gype04G1340.1;Gype05G0299.1;Gype06G0610.1;Gype07G2382.1;Gype07G2899.1;Gype07G2981.1;Gype08G0609.1;Gype08G0659.1;Gype09G0975.1;Gype10G0164.1;Gype11G2173.1;Gype11G2304.1;Gype11G2391.1;Gype03G1060.1;Gype03G1105.1;Gype04G0639.1;Gype04G0647.1;Gype04G0765.1;Gype04G0770.1;Gype04G0775.1;Gype04G0776.1;Gype02G0008.1;Gype04G0108.1;Gype05G0112.1;Gype05G0832.1;Gype06G0769.1;Gype06G0852.1;Gype08G0990.1;Gype08G2694.1;Gype09G1976.1;Gype09G2063.1;Gype09G2162.1;Gype10G0192.1;Gype10G0194.1;Gype10G0196.1;Gype10G0208.1;Gype10G1069.1;Gype10G1072.1;Gype10G1077.1;Gype01G0629.1;Gype03G1998.1;Gype06G0215.1;Gype06G0701.1;Gype09G2015.1;Gype09G2049.1;Gype11G1500.1;Gype03G2139.1;Gype05G0858.1;Gype09G0441.1 | K01807+K00028+K00134+K00025+K03841 |
| Monoterpenoid biosynthesis | 23 (1.03%) | 38 (0.18%) | 1.20E-13 | 1.70E-12 | ko00902 | Gype01G0468.1;Gype01G0469.1;Gype04G0304.1;Gype04G0630.1;Gype04G0632.1;Gype04G0633.1;Gype05G0369.1;Gype05G0370.1;Gype05G0371.1;Gype05G0383.1;Gype05G0385.1;Gype05G0386.1;Gype07G2591.1;Gype11G3121.1;Gype11G3123.1;Gype11G3124.1;Gype11G3126.1;Gype11G3128.1;Gype11G3130.1;Gype11G3132.1;Gype11G3133.1;Gype11G3134.1;Gype11G3131.1 | K07385+K18108 |
| Glucosinolate biosynthesis | 16 (0.71%) | 20 (0.09%) | 8.31E-13 | 1.01E-11 | ko00966 | Gype07G2259.1;Gype07G2329.1;Gype07G2330.1;Gype07G2331.1;Gype07G2332.1;Gype07G2333.1;Gype07G2334.1;Gype07G2335.1;Gype07G2336.1;Gype07G2337.1;Gype07G2338.1;Gype07G2339.1;Gype07G2340.1;Gype01G0480.1;Gype05G0314.1;Gype08G1569.1 | K12153 |
| Cyanoamino acid metabolism | 60 (2.68%) | 228 (1.08%) | 2.00E-11 | 2.13E-10 | ko00460 | Gype06G1280.1;Gype06G1286.1;Gype06G1296.1;Gype06G1298.1;Gype06G1301.1;Gype07G0919.1;Gype02G1541.1;Gype07G2259.1;Gype07G2329.1;Gype07G2330.1;Gype07G2331.1;Gype07G2332.1;Gype07G2333.1;Gype07G2334.1;Gype07G2335.1;Gype07G2336.1;Gype07G2337.1;Gype07G2338.1;Gype07G2339.1;Gype07G2340.1;Gype01G0369.1;Gype05G1565.1;Gype05G1567.1;Gype05G1568.1;Gype05G2051.1;Gype05G2052.1;Gype05G2053.1;Gype05G2054.1;Gype01G0500.1;Gype07G2271.1;Gype08G0098.1;Gype08G0099.1;Gype08G0101.1;Gype08G0103.1;Gype08G0104.1;Gype08G0105.1;Gype08G0106.1;Gype08G0108.1;Gype08G0109.1;Gype08G0115.1;Gype08G0116.1;Gype08G0117.1;Gype08G0118.1;Gype08G0119.1;Gype08G0120.1;Gype08G0121.1;Gype07G2308.1;Gype01G0480.1;Gype05G0314.1;Gype08G1569.1;Gype08G0623.1;Gype08G0624.1;Gype04G0929.1;Gype07G2265.1;Gype07G2269.1;Gype02G1249.1;Gype06G1291.1;Gype06G1292.1;Gype01G0546.1;Gype06G1305.1 | K18592+K00495+K12153+K05350+K01188 |
| Indole alkaloid biosynthesis | 14 (0.62%) | 18 (0.09%) | 4.70E-11 | 4.44E-10 | ko00901 | Gype08G1056.1;Gype08G1057.1;Gype08G1058.1;Gype08G1060.1;Gype08G1062.1;Gype08G1066.1;Gype08G1068.1;Gype08G1072.1;Gype08G1073.1;Gype08G1075.1;Gype08G1077.1;Gype08G1080.1;Gype08G1084.1;Gype10G0826.1 | K01593 |
| Phenylalanine metabolism | 43 (1.92%) | 165 (0.78%) | 1.86E-08 | 1.58E-07 | ko00360 | Gype08G1056.1;Gype08G1057.1;Gype08G1058.1;Gype08G1060.1;Gype08G1062.1;Gype08G1066.1;Gype08G1068.1;Gype08G1072.1;Gype08G1073.1;Gype08G1075.1;Gype08G1077.1;Gype08G1080.1;Gype08G1084.1;Gype10G0826.1;Gype10G0501.1;Gype11G2189.1;Gype11G2191.1;Gype11G2193.1;Gype11G2196.1;Gype11G2199.1;Gype11G2200.1;Gype11G2320.1;Gype11G2322.1;Gype11G2324.1;Gype11G2325.1;Gype11G2327.1;Gype11G2328.1;Gype07G2427.1;Gype08G0400.1;Gype09G1032.1;Gype09G1971.1;Gype09G2007.1;Gype09G2019.1;Gype09G2052.1;Gype04G0524.1;Gype07G2308.1;Gype11G2190.1;Gype11G2192.1;Gype11G2194.1;Gype11G2198.1;Gype11G2321.1;Gype11G2323.1;Gype11G2326.1 | K01593+K01426+K00811+K01904 |
| Photosynthesis - antenna proteins | 15 (0.67%) | 32 (0.15%) | 2.37E-07 | 1.83E-06 | ko00196 | Gype02G1081.1;Gype02G1085.1;Gype02G1086.1;Gype02G1087.1;Gype02G1089.1;Gype07G1840.1;Gype08G0809.1;Gype08G0836.1;Gype08G2721.1;Gype10G1415.1;Gype10G1428.1;Gype11G1770.1;Gype11G1771.1;Gype11G1772.1;Gype11G1774.1 | K08912 |
| Starch and sucrose metabolism | 84 (3.75%) | 465 (2.21%) | 8.51E-07 | 6.03E-06 | ko00500 | Gype06G1876.1;Gype06G1883.1;Gype06G1884.1;Gype06G1888.1;Gype06G1889.1;Gype06G1890.1;Gype06G1893.1;Gype06G1896.1;Gype06G1898.1;Gype06G1899.1;Gype06G1901.1;Gype06G1903.1;Gype06G1904.1;Gype06G1905.1;Gype06G1906.1;Gype06G1908.1;Gype06G1910.1;Gype06G1914.1;Gype06G1916.1;Gype06G1917.1;Gype06G1918.1;Gype06G1919.1;Gype06G1920.1;Gype06G1922.1;Gype06G1923.1;Gype09G2055.1;Gype09G2056.1;Gype09G2057.1;Gype09G2058.1;Gype02G0381.1;Gype03G0840.1;Gype03G1061.1;Gype03G2504.1;Gype04G1470.1;Gype05G0245.1;Gype06G0917.1;Gype07G2434.1;Gype08G0870.1;Gype01G0523.1;Gype01G0526.1;Gype01G0527.1;Gype01G0587.1;Gype07G2021.1;Gype07G2022.1;Gype01G0369.1;Gype05G1565.1;Gype05G1567.1;Gype05G1568.1;Gype05G2051.1;Gype05G2052.1;Gype05G2053.1;Gype05G2054.1;Gype01G0244.1;Gype04G0076.1;Gype05G0373.1;Gype06G0030.1;Gype06G0324.1;Gype07G1649.1;Gype07G2855.1;Gype08G0009.1;Gype10G1043.1;Gype01G0500.1;Gype07G2271.1;Gype08G0098.1;Gype08G0099.1;Gype08G0101.1;Gype08G0103.1;Gype08G0104.1;Gype08G0105.1;Gype08G0106.1;Gype08G0108.1;Gype08G0109.1;Gype08G0115.1;Gype08G0116.1;Gype08G0117.1;Gype08G0118.1;Gype08G0119.1;Gype08G0120.1;Gype08G0121.1;Gype07G2308.1;Gype04G0929.1;Gype07G2265.1;Gype07G2269.1;Gype01G0546.1 | K01193+K19893+K19891+K19892+K05350+K01188 |
| Arachidonic acid metabolism | 26 (1.16%) | 90 (0.43%) | 1.43E-06 | 9.37E-06 | ko00590 | Gype06G1280.1;Gype06G1286.1;Gype06G1296.1;Gype06G1298.1;Gype06G1301.1;Gype07G0919.1;Gype06G0761.1;Gype06G0764.1;Gype06G0765.1;Gype06G0845.1;Gype06G0848.1;Gype10G1309.1;Gype10G1312.1;Gype10G1313.1;Gype10G1314.1;Gype10G1327.1;Gype10G1328.1;Gype10G1329.1;Gype10G1330.1;Gype10G1332.1;Gype08G0623.1;Gype08G0624.1;Gype02G1249.1;Gype06G1291.1;Gype06G1292.1;Gype06G1305.1 | K18592+K08726 |
| Betalain biosynthesis | 14 (0.62%) | 33 (0.16%) | 2.66E-06 | 1.62E-05 | ko00965 | Gype08G1056.1;Gype08G1057.1;Gype08G1058.1;Gype08G1060.1;Gype08G1062.1;Gype08G1066.1;Gype08G1068.1;Gype08G1072.1;Gype08G1073.1;Gype08G1075.1;Gype08G1077.1;Gype08G1080.1;Gype08G1084.1;Gype10G0826.1 | K01593 |
| Brassinosteroid biosynthesis | 17 (0.76%) | 51 (0.24%) | 1.16E-05 | 6.59E-05 | ko00905 | Gype07G2912.1;Gype07G2913.1;Gype07G2916.1;Gype07G2922.1;Gype07G2930.1;Gype04G0006.1;Gype04G0380.1;Gype04G0383.1;Gype10G1533.1;Gype10G1851.1;Gype10G1853.1;Gype10G1854.1;Gype10G1855.1;Gype10G1856.1;Gype10G1857.1;Gype10G1858.1;Gype10G1860.1 | K15639+K20623 |
| Arginine and proline metabolism | 32 (1.43%) | 142 (0.67%) | 3.07E-05 | 1.63E-04 | ko00330 | Gype10G0501.1;Gype11G2189.1;Gype11G2191.1;Gype11G2193.1;Gype11G2196.1;Gype11G2199.1;Gype11G2200.1;Gype11G2320.1;Gype11G2322.1;Gype11G2324.1;Gype11G2325.1;Gype11G2327.1;Gype11G2328.1;Gype07G2427.1;Gype08G0400.1;Gype09G1032.1;Gype09G1971.1;Gype09G2007.1;Gype09G2019.1;Gype09G2052.1;Gype04G0524.1;Gype11G2190.1;Gype11G2192.1;Gype11G2194.1;Gype11G2198.1;Gype11G2321.1;Gype11G2323.1;Gype11G2326.1;Gype02G1810.1;Gype05G1947.1;Gype05G1948.1;Gype05G1949.1 | K01426+K00811+K14329 |
| Taurine and hypotaurine metabolism | 12 (0.54%) | 32 (0.15%) | 6.05E-05 | 3.03E-04 | ko00430 | Gype06G1280.1;Gype06G1286.1;Gype06G1296.1;Gype06G1298.1;Gype06G1301.1;Gype07G0919.1;Gype08G0623.1;Gype08G0624.1;Gype02G1249.1;Gype06G1291.1;Gype06G1292.1;Gype06G1305.1 | K18592 |
| Diterpenoid biosynthesis | 20 (0.89%) | 76 (0.36%) | 9.80E-05 | 4.63E-04 | ko00904 | Gype07G0742.1;Gype11G2588.1;Gype11G2591.1;Gype11G2749.1;Gype11G2751.1;Gype11G0127.1;Gype11G0128.1;Gype11G0129.1;Gype11G0742.1;Gype11G0743.1;Gype11G0744.1;Gype01G0563.1;Gype01G0564.1;Gype01G0565.1;Gype01G0566.1;Gype01G0567.1;Gype01G0642.1;Gype01G0644.1;Gype01G0655.1;Gype01G0656.1 | K04123+K04124+K04125+K05282 |
| Isoquinoline alkaloid biosynthesis | 15 (0.67%) | 51 (0.24%) | 0.000188263 | 8.42E-04 | ko00950 | Gype08G1056.1;Gype08G1057.1;Gype08G1058.1;Gype08G1060.1;Gype08G1062.1;Gype08G1066.1;Gype08G1068.1;Gype08G1072.1;Gype08G1073.1;Gype08G1075.1;Gype08G1077.1;Gype08G1080.1;Gype08G1084.1;Gype10G0826.1;Gype04G0524.1 | K01593+K00811 |
| Phosphonate and phosphinate metabolism | 9 (0.4%) | 22 (0.1%) | 0.000236363 | 1.00E-03 | ko00440 | Gype08G1531.1;Gype08G1532.1;Gype08G1533.1;Gype08G1534.1;Gype08G1535.1;Gype08G1537.1;Gype08G1539.1;Gype08G1540.1;Gype08G1541.1 | K00993 |
| Biosynthesis of secondary metabolites | 302 (13.47%) | 2383 (11.32%) | 0.000487326 | 1.97E-03 | ko01110 | Gype07G2715.1;Gype05G1805.1;Gype01G0468.1;Gype01G0469.1;Gype04G0304.1;Gype04G0630.1;Gype04G0632.1;Gype04G0633.1;Gype05G0369.1;Gype05G0370.1;Gype05G0371.1;Gype05G0383.1;Gype05G0385.1;Gype05G0386.1;Gype07G2591.1;Gype11G3120.1;Gype11G3121.1;Gype11G3123.1;Gype11G3124.1;Gype11G3126.1;Gype11G3128.1;Gype11G3130.1;Gype11G3132.1;Gype11G3133.1;Gype11G3134.1;Gype01G0409.1;Gype01G0442.1;Gype02G0430.1;Gype04G0415.1;Gype04G1340.1;Gype05G0299.1;Gype06G0610.1;Gype07G2382.1;Gype07G2899.1;Gype07G2981.1;Gype08G0609.1;Gype08G0659.1;Gype09G0975.1;Gype10G0164.1;Gype11G2173.1;Gype11G2304.1;Gype11G2391.1;Gype02G1541.1;Gype11G3131.1;Gype07G2259.1;Gype07G2329.1;Gype07G2330.1;Gype07G2331.1;Gype07G2332.1;Gype07G2333.1;Gype07G2334.1;Gype07G2335.1;Gype07G2336.1;Gype07G2337.1;Gype07G2338.1;Gype07G2339.1;Gype07G2340.1;Gype05G0662.1;Gype08G1056.1;Gype08G1057.1;Gype08G1058.1;Gype08G1060.1;Gype08G1062.1;Gype08G1066.1;Gype08G1068.1;Gype08G1072.1;Gype08G1073.1;Gype08G1075.1;Gype08G1077.1;Gype08G1080.1;Gype08G1084.1;Gype10G0826.1;Gype08G1531.1;Gype08G1532.1;Gype08G1533.1;Gype08G1534.1;Gype08G1535.1;Gype08G1537.1;Gype08G1539.1;Gype08G1540.1;Gype08G1541.1;Gype02G0825.1;Gype02G0826.1;Gype02G0827.1;Gype02G0829.1;Gype02G0831.1;Gype02G0833.1;Gype07G0742.1;Gype11G2588.1;Gype11G2591.1;Gype11G2749.1;Gype11G2751.1;Gype10G0886.1;Gype10G0892.1;Gype10G0894.1;Gype10G0895.1;Gype10G0897.1;Gype10G0898.1;Gype10G0941.1;Gype01G0465.1;Gype01G0472.1;Gype02G0434.1;Gype02G0507.1;Gype06G0658.1;Gype06G2280.1;Gype06G2281.1;Gype06G2283.1;Gype06G2284.1;Gype01G0807.1;Gype01G0809.1;Gype01G0812.1;Gype03G2237.1;Gype05G0216.1;Gype05G0219.1;Gype05G0327.1;Gype05G0399.1;Gype05G0400.1;Gype05G0402.1;Gype05G0409.1;Gype05G0415.1;Gype05G0416.1;Gype05G0424.1;Gype05G0426.1;Gype08G0804.1;Gype08G2835.1;Gype08G2854.1;Gype11G0127.1;Gype11G0128.1;Gype11G0129.1;Gype11G0742.1;Gype11G0743.1;Gype11G0744.1;Gype09G0581.1;Gype09G0582.1;Gype09G0583.1;Gype09G0584.1;Gype09G0585.1;Gype09G0586.1;Gype09G0587.1;Gype09G0588.1;Gype09G0589.1;Gype09G0591.1;Gype09G0592.1;Gype09G0593.1;Gype09G0594.1;Gype09G0595.1;Gype09G0596.1;Gype09G0597.1;Gype09G0598.1;Gype02G1432.1;Gype02G0008.1;Gype04G0108.1;Gype05G0112.1;Gype05G0832.1;Gype06G0769.1;Gype06G0852.1;Gype08G0990.1;Gype08G2694.1;Gype09G1976.1;Gype09G2063.1;Gype09G2162.1;Gype04G0524.1;Gype11G0517.1;Gype11G0518.1;Gype11G0519.1;Gype11G0521.1;Gype11G0522.1;Gype11G0557.1;Gype11G0559.1;Gype11G0562.1;Gype11G0563.1;Gype01G0369.1;Gype05G1565.1;Gype05G1567.1;Gype05G1568.1;Gype05G2051.1;Gype05G2052.1;Gype05G2053.1;Gype05G2054.1;Gype04G0391.1;Gype04G0393.1;Gype04G0423.1;Gype10G0192.1;Gype10G0194.1;Gype10G0196.1;Gype10G0208.1;Gype10G1069.1;Gype10G1072.1;Gype10G1077.1;Gype01G0629.1;Gype03G1998.1;Gype06G0215.1;Gype06G0701.1;Gype09G2015.1;Gype09G2049.1;Gype11G1500.1;Gype03G0041.1;Gype03G0214.1;Gype07G1067.1;Gype09G2069.1;Gype09G2070.1;Gype09G2071.1;Gype09G2072.1;Gype11G2641.1;Gype11G2646.1;Gype11G2747.1;Gype11G2752.1;Gype11G2753.1;Gype11G2761.1;Gype11G2763.1;Gype11G2764.1;Gype11G2773.1;Gype11G2774.1;Gype11G2775.1;Gype11G2776.1;Gype11G2777.1;Gype11G2778.1;Gype11G2782.1;Gype01G0500.1;Gype07G2271.1;Gype08G0098.1;Gype08G0099.1;Gype08G0101.1;Gype08G0103.1;Gype08G0104.1;Gype08G0105.1;Gype08G0106.1;Gype08G0108.1;Gype08G0109.1;Gype08G0115.1;Gype08G0116.1;Gype08G0117.1;Gype08G0118.1;Gype08G0119.1;Gype08G0120.1;Gype08G0121.1;Gype03G2139.1;Gype07G2308.1;Gype01G0480.1;Gype05G0314.1;Gype08G1569.1;Gype10G0512.1;Gype11G1723.1;Gype02G0953.1;Gype02G0954.1;Gype02G0955.1;Gype02G0956.1;Gype02G0957.1;Gype02G0959.1;Gype02G0960.1;Gype02G0963.1;Gype02G0968.1;Gype02G0981.1;Gype02G0983.1;Gype04G0929.1;Gype07G2265.1;Gype07G2269.1;Gype01G0563.1;Gype01G0564.1;Gype01G0565.1;Gype01G0566.1;Gype01G0567.1;Gype01G0642.1;Gype01G0644.1;Gype01G0655.1;Gype01G0656.1;Gype01G0546.1;Gype02G0635.1;Gype02G0637.1;Gype02G0639.1;Gype02G0640.1;Gype02G0641.1;Gype02G0644.1;Gype02G0645.1;Gype02G0646.1;Gype05G0305.1;Gype09G1284.1;Gype10G1851.1;Gype10G1853.1;Gype10G1854.1;Gype10G1855.1;Gype10G1856.1;Gype10G1857.1;Gype10G1858.1;Gype10G1860.1;Gype05G1946.1;Gype10G1597.1;Gype10G1599.1;Gype10G1600.1;Gype10G1601.1;Gype05G0858.1;Gype09G0441.1;Gype09G2068.1;Gype11G2147.1;Gype11G2282.1;Gype11G2647.1;Gype11G0520.1;Gype11G0558.1;Gype11G0560.1;Gype02G0181.1;Gype02G0183.1;Gype02G0187.1 | K00514+K09841+K07385+K18108+K12742+K01807+K00495+K12153+K01593+K00993+K00895+K04123+K06125+K13065+K22395+K04124+K04125+K13260+K22995+K00430+K00134+K00811+K00454+K05350+K08730+K00025+K17912+K01188+K03841+K01904+K09833+K05282+K05359+K20623+K05277+K05278+K13493 |
| Pentose phosphate pathway | 26 (1.16%) | 124 (0.59%) | 0.000542695 | 2.10E-03 | ko00030 | Gype01G0409.1;Gype01G0442.1;Gype02G0430.1;Gype04G0415.1;Gype04G1340.1;Gype05G0299.1;Gype06G0610.1;Gype07G2382.1;Gype07G2899.1;Gype07G2981.1;Gype08G0609.1;Gype08G0659.1;Gype09G0975.1;Gype10G0164.1;Gype11G2173.1;Gype11G2304.1;Gype11G2391.1;Gype02G0825.1;Gype02G0826.1;Gype02G0827.1;Gype02G0829.1;Gype02G0831.1;Gype02G0833.1;Gype03G2139.1;Gype05G0858.1;Gype09G0441.1 | K01807+K00895+K03841 |
| Phenylpropanoid biosynthesis | 72 (3.21%) | 462 (2.19%) | 0.000613692 | 2.27E-03 | ko00940 | Gype01G0465.1;Gype01G0472.1;Gype02G0434.1;Gype02G0507.1;Gype06G0658.1;Gype06G2280.1;Gype06G2281.1;Gype06G2283.1;Gype06G2284.1;Gype01G0807.1;Gype01G0809.1;Gype01G0812.1;Gype03G2237.1;Gype05G0216.1;Gype05G0219.1;Gype05G0327.1;Gype05G0399.1;Gype05G0400.1;Gype05G0402.1;Gype05G0409.1;Gype05G0415.1;Gype05G0416.1;Gype05G0424.1;Gype05G0426.1;Gype08G0804.1;Gype08G2835.1;Gype08G2854.1;Gype02G1432.1;Gype01G0369.1;Gype05G1565.1;Gype05G1567.1;Gype05G1568.1;Gype05G2051.1;Gype05G2052.1;Gype05G2053.1;Gype05G2054.1;Gype01G0500.1;Gype07G2271.1;Gype08G0098.1;Gype08G0099.1;Gype08G0101.1;Gype08G0103.1;Gype08G0104.1;Gype08G0105.1;Gype08G0106.1;Gype08G0108.1;Gype08G0109.1;Gype08G0115.1;Gype08G0116.1;Gype08G0117.1;Gype08G0118.1;Gype08G0119.1;Gype08G0120.1;Gype08G0121.1;Gype07G2308.1;Gype10G0512.1;Gype02G0953.1;Gype02G0954.1;Gype02G0955.1;Gype02G0956.1;Gype02G0957.1;Gype02G0959.1;Gype02G0960.1;Gype02G0963.1;Gype02G0968.1;Gype02G0981.1;Gype02G0983.1;Gype04G0929.1;Gype07G2265.1;Gype07G2269.1;Gype01G0546.1;Gype05G1946.1 | K13065+K22395+K00430+K05350+K01188+K01904 |
| RNA polymerase | 32 (1.43%) | 168 (0.8%) | 0.000797645 | 2.82E-03 | ko03020 | Gype10G0895.1;Gype10G0896.1;Gype10G0897.1;Gype09G1971.1;Gype09G2019.1;Gype05G0135.1;Gype05G0142.1;Gype05G0166.1;Gype06G0494.1;Gype08G0008.1;Gype08G0010.1;Gype09G1986.1;Gype09G2051.1;Gype11G0152.1;Gype11G0156.1;Gype11G0075.1;Gype11G0791.1;Gype11G0795.1;Gype02G1418.1;Gype02G1424.1;Gype09G1888.1;Gype04G0187.1;Gype11G1063.1;Gype11G1064.1;Gype04G0391.1;Gype04G0393.1;Gype04G0395.1;Gype04G0397.1;Gype04G0419.1;Gype04G0421.1;Gype04G0423.1;Gype02G1431.1 | K03006+K03021 |
| Galactose metabolism | 31 (1.38%) | 173 (0.82%) | 0.002615546 | 8.89E-03 | ko00052 | Gype06G1876.1;Gype06G1883.1;Gype06G1884.1;Gype06G1888.1;Gype06G1889.1;Gype06G1890.1;Gype06G1893.1;Gype06G1896.1;Gype06G1898.1;Gype06G1899.1;Gype06G1901.1;Gype06G1903.1;Gype06G1904.1;Gype06G1905.1;Gype06G1906.1;Gype06G1908.1;Gype06G1910.1;Gype06G1914.1;Gype06G1916.1;Gype06G1917.1;Gype06G1918.1;Gype06G1919.1;Gype06G1920.1;Gype06G1922.1;Gype06G1923.1;Gype09G2055.1;Gype09G2056.1;Gype09G2057.1;Gype09G2058.1;Gype08G0106.1;Gype08G0109.1 | K01193+K01229 |
| Pentose and glucuronate interconversions | 49 (2.19%) | 310 (1.47%) | 0.003153738 | 1.03E-02 | ko00040 | Gype04G0363.1;Gype04G0771.1;Gype01G0276.1;Gype01G0760.1;Gype03G2401.1;Gype02G1493.1;Gype02G1495.1;Gype02G1496.1;Gype02G1497.1;Gype02G1498.1;Gype02G1499.1;Gype02G1606.1;Gype02G1607.1;Gype10G1375.1;Gype10G1789.1;Gype01G0309.1;Gype01G1879.1;Gype06G0184.1;Gype06G0448.1;Gype08G0995.1;Gype09G1533.1;Gype10G0829.1;Gype11G2900.1;Gype01G0977.1;Gype03G1435.1;Gype05G0043.1;Gype06G0625.1;Gype08G0432.1;Gype10G0972.1;Gype10G1397.1;Gype03G1515.1;Gype04G0207.1;Gype04G0208.1;Gype04G0209.1;Gype04G0210.1;Gype04G0211.1;Gype04G0212.1;Gype05G0606.1;Gype05G0607.1;Gype07G0280.1;Gype07G0281.1;Gype07G1052.1;Gype08G0428.1;Gype09G2210.1;Gype09G2332.1;Gype11G0164.1;Gype07G0020.1;Gype10G0326.1;Gype11G2840.1 | K01051+K01213 |
| Linoleic acid metabolism | 12 (0.54%) | 54 (0.26%) | 0.01008518 | 3.16E-02 | ko00591 | Gype11G0517.1;Gype11G0518.1;Gype11G0519.1;Gype11G0521.1;Gype11G0522.1;Gype11G0557.1;Gype11G0559.1;Gype11G0562.1;Gype11G0563.1;Gype11G0520.1;Gype11G0558.1;Gype11G0560.1 | K00454 |
| Anthocyanin biosynthesis | 4 (0.18%) | 9 (0.04%) | 0.01040308 | 3.16E-02 | ko00942 | Gype02G1810.1;Gype05G1946.1;Gype05G1947.1;Gype05G1948.1 | K21383 |
| AGE-RAGE signaling pathway in diabetic complications | 18 (0.8%) | 97 (0.46%) | 0.01332245 | 3.90E-02 | ko04933 | Gype01G0537.1;Gype04G0320.1;Gype04G0321.1;Gype04G0322.1;Gype04G0323.1;Gype04G0324.1;Gype04G0325.1;Gype04G0337.1;Gype04G0338.1;Gype04G0339.1;Gype11G2675.1;Gype11G2676.1;Gype11G2677.1;Gype11G2687.1;Gype11G2688.1;Gype11G2701.1;Gype11G2702.1;Gype11G2703.1 | K04371 |
| Tyrosine metabolism | 15 (0.67%) | 82 (0.39%) | 0.02539547 | 7.20E-02 | ko00350 | Gype08G1056.1;Gype08G1057.1;Gype08G1058.1;Gype08G1060.1;Gype08G1062.1;Gype08G1066.1;Gype08G1068.1;Gype08G1072.1;Gype08G1073.1;Gype08G1075.1;Gype08G1077.1;Gype08G1080.1;Gype08G1084.1;Gype10G0826.1;Gype04G0524.1 | K01593+K00811 |
| Carbon metabolism | 62 (2.77%) | 461 (2.19%) | 0.03213665 | 8.81E-02 | ko01200 | Gype01G0409.1;Gype01G0442.1;Gype02G0430.1;Gype04G0415.1;Gype04G1340.1;Gype05G0299.1;Gype06G0610.1;Gype07G2382.1;Gype07G2899.1;Gype07G2981.1;Gype08G0609.1;Gype08G0659.1;Gype09G0975.1;Gype10G0164.1;Gype11G2173.1;Gype11G2304.1;Gype11G2391.1;Gype06G0761.1;Gype06G0764.1;Gype06G0845.1;Gype10G1312.1;Gype10G1313.1;Gype10G1314.1;Gype10G1328.1;Gype10G1329.1;Gype10G1330.1;Gype03G1060.1;Gype03G1105.1;Gype04G0639.1;Gype04G0647.1;Gype04G0765.1;Gype04G0770.1;Gype04G0775.1;Gype04G0776.1;Gype02G0008.1;Gype04G0108.1;Gype05G0112.1;Gype05G0832.1;Gype06G0769.1;Gype06G0852.1;Gype08G0990.1;Gype08G2694.1;Gype09G1976.1;Gype09G2063.1;Gype09G2162.1;Gype10G0192.1;Gype10G0194.1;Gype10G0196.1;Gype10G0208.1;Gype10G1069.1;Gype10G1072.1;Gype10G1077.1;Gype01G0629.1;Gype03G1998.1;Gype06G0215.1;Gype06G0701.1;Gype09G2015.1;Gype09G2049.1;Gype11G1500.1;Gype03G2139.1;Gype05G0858.1;Gype09G0441.1 | K01807+K01079+K00028+K00134+K00025+K03841 |
| Ether lipid metabolism | 13 (0.58%) | 75 (0.36%) | 0.05226214 | 1.39E-01 | ko00565 | Gype08G1531.1;Gype08G1532.1;Gype08G1533.1;Gype08G1534.1;Gype08G1535.1;Gype08G1537.1;Gype08G1539.1;Gype08G1540.1;Gype08G1541.1;Gype09G2110.1;Gype02G0181.1;Gype02G0183.1;Gype02G0187.1 | K00993+K13510+K04628 |
| Sesquiterpenoid and triterpenoid biosynthesis | 12 (0.54%) | 69 (0.33%) | 0.05931745 | 1.53E-01 | ko00909 | Gype01G0162.1;Gype03G0859.1;Gype03G0860.1;Gype08G0686.1;Gype08G0688.1;Gype08G0705.1;Gype08G0743.1;Gype08G0744.1;Gype11G0808.1;Gype03G0861.1;Gype03G0862.1;Gype08G0687.1 | K15803 |
| MAPK signaling pathway - plant | 70 (3.12%) | 567 (2.69%) | 0.1057193 | 2.64E-01 | ko04016 | Gype07G2981.1;Gype08G0659.1;Gype11G2173.1;Gype11G2304.1;Gype01G0516.1;Gype01G0581.1;Gype01G0582.1;Gype01G0585.1;Gype09G0200.1;Gype10G1732.1;Gype10G1733.1;Gype10G1737.1;Gype10G1741.1;Gype10G1742.1;Gype10G1745.1;Gype10G1746.1;Gype10G1748.1;Gype10G1749.1;Gype10G1750.1;Gype10G1752.1;Gype10G1758.1;Gype07G1669.1;Gype07G2911.1;Gype07G2912.1;Gype07G2913.1;Gype07G2915.1;Gype07G2920.1;Gype07G2921.1;Gype07G2923.1;Gype07G2930.1;Gype07G2931.1;Gype08G0463.1;Gype08G0508.1;Gype10G0356.1;Gype10G0786.1;Gype10G0792.1;Gype05G0141.1;Gype09G2000.1;Gype11G0791.1;Gype02G1424.1;Gype09G1886.1;Gype10G0311.1;Gype04G0005.1;Gype04G0006.1;Gype04G0010.1;Gype04G0380.1;Gype04G0383.1;Gype04G0384.1;Gype10G1533.1;Gype04G0325.1;Gype09G1995.1;Gype03G2176.1;Gype03G2179.1;Gype03G2180.1;Gype03G2181.1;Gype03G2218.1;Gype03G2219.1;Gype03G2221.1;Gype03G2224.1;Gype05G0675.1;Gype11G2855.1;Gype11G2856.1;Gype11G2859.1;Gype11G2860.1;Gype11G2861.1;Gype11G2862.1;Gype11G2863.1;Gype11G2864.1;Gype11G2865.1;Gype02G1427.1 | K20535+K16224+K20716+K13420+K20718+K20538+K13416 |
| Biosynthesis of amino acids | 57 (2.54%) | 467 (2.22%) | 0.1523798 | 3.70E-01 | ko01230 | Gype01G0409.1;Gype01G0442.1;Gype02G0430.1;Gype04G0415.1;Gype04G1340.1;Gype05G0299.1;Gype06G0610.1;Gype07G2382.1;Gype07G2899.1;Gype07G2981.1;Gype08G0609.1;Gype08G0659.1;Gype09G0975.1;Gype10G0164.1;Gype11G2173.1;Gype11G2304.1;Gype11G2391.1;Gype06G0761.1;Gype06G0764.1;Gype06G0845.1;Gype10G1312.1;Gype10G1313.1;Gype10G1314.1;Gype10G1328.1;Gype10G1329.1;Gype10G1330.1;Gype02G0008.1;Gype04G0108.1;Gype05G0112.1;Gype05G0832.1;Gype06G0769.1;Gype06G0852.1;Gype08G0990.1;Gype08G2694.1;Gype09G1976.1;Gype09G2063.1;Gype09G2162.1;Gype04G0524.1;Gype01G0629.1;Gype03G1998.1;Gype06G0215.1;Gype06G0701.1;Gype09G2015.1;Gype09G2049.1;Gype11G1500.1;Gype02G0635.1;Gype02G0637.1;Gype02G0639.1;Gype02G0640.1;Gype02G0641.1;Gype02G0644.1;Gype02G0645.1;Gype02G0646.1;Gype05G0305.1;Gype09G1284.1;Gype05G0858.1;Gype09G0441.1 | K01807+K01079+K00134+K00811+K05359 |
| 2-Oxocarboxylic acid metabolism | 17 (0.76%) | 124 (0.59%) | 0.1668637 | 3.94E-01 | ko01210 | Gype07G2259.1;Gype07G2329.1;Gype07G2330.1;Gype07G2331.1;Gype07G2332.1;Gype07G2333.1;Gype07G2334.1;Gype07G2335.1;Gype07G2336.1;Gype07G2337.1;Gype07G2338.1;Gype07G2339.1;Gype07G2340.1;Gype04G0524.1;Gype01G0480.1;Gype05G0314.1;Gype08G1569.1 | K12153+K00811 |
| Stilbenoid, diarylheptanoid and gingerol biosynthesis | 10 (0.45%) | 68 (0.32%) | 0.1834484 | 4.21E-01 | ko00945 | Gype01G0465.1;Gype01G0472.1;Gype02G0434.1;Gype02G0507.1;Gype06G0658.1;Gype06G2280.1;Gype06G2281.1;Gype06G2283.1;Gype06G2284.1;Gype05G1946.1 | K13065 |
| Biosynthesis of secondary metabolites - unclassified | 1 (0.04%) | 3 (0.01%) | 0.2866769 | 6.41E-01 | ko00999 | Gype09G0585.1 | K22995 |
| Cutin, suberine and wax biosynthesis | 7 (0.31%) | 54 (0.26%) | 0.3511517 | 7.65E-01 | ko00073 | Gype06G1584.1;Gype10G0138.1;Gype10G0139.1;Gype10G0140.1;Gype10G0141.1;Gype10G0142.1;Gype10G0143.1 | K17991+K15406 |
| Phenylalanine, tyrosine and tryptophan biosynthesis | 11 (0.49%) | 90 (0.43%) | 0.3618468 | 7.69E-01 | ko00400 | Gype04G0524.1;Gype02G0635.1;Gype02G0637.1;Gype02G0639.1;Gype02G0640.1;Gype02G0641.1;Gype02G0644.1;Gype02G0645.1;Gype02G0646.1;Gype05G0305.1;Gype09G1284.1 | K00811+K05359 |
| Glycolysis / Gluconeogenesis | 25 (1.12%) | 219 (1.04%) | 0.3875641 | 8.03E-01 | ko00010 | Gype02G0825.1;Gype02G0826.1;Gype02G0827.1;Gype02G0829.1;Gype02G0831.1;Gype02G0833.1;Gype02G0008.1;Gype04G0108.1;Gype05G0112.1;Gype05G0832.1;Gype06G0769.1;Gype06G0852.1;Gype08G0990.1;Gype08G2694.1;Gype09G1976.1;Gype09G2063.1;Gype09G2162.1;Gype01G0629.1;Gype03G1998.1;Gype06G0215.1;Gype06G0701.1;Gype09G2015.1;Gype09G2049.1;Gype11G1500.1;Gype03G2139.1 | K00895+K00134+K03841 |
| Mismatch repair | 10 (0.45%) | 91 (0.43%) | 0.5080257 | 1.00E+00 | ko03430 | Gype03G0364.1;Gype05G0321.1;Gype05G0441.1;Gype05G0444.1;Gype05G0450.1;Gype05G0456.1;Gype05G0470.1;Gype05G0476.1;Gype05G0480.1;Gype05G0492.1 | K07456 |
| alpha-Linolenic acid metabolism | 12 (0.54%) | 112 (0.53%) | 0.5363708 | 1.00E+00 | ko00592 | Gype11G0517.1;Gype11G0518.1;Gype11G0519.1;Gype11G0521.1;Gype11G0522.1;Gype11G0557.1;Gype11G0559.1;Gype11G0562.1;Gype11G0563.1;Gype11G0520.1;Gype11G0558.1;Gype11G0560.1 | K00454 |
| Protein processing in endoplasmic reticulum | 48 (2.14%) | 461 (2.19%) | 0.5890333 | 1.00E+00 | ko04141 | Gype01G1106.1;Gype01G1235.1;Gype05G1389.1;Gype05G1529.1;Gype05G1530.1;Gype05G1696.1;Gype05G1732.1;Gype05G1733.1;Gype05G1752.1;Gype05G1753.1;Gype05G1803.1;Gype05G1804.1;Gype05G1806.1;Gype05G1807.1;Gype05G1810.1;Gype05G1811.1;Gype05G1888.1;Gype05G1910.1;Gype05G1981.1;Gype05G2090.1;Gype05G2092.1;Gype05G2093.1;Gype06G2306.1;Gype06G3035.1;Gype08G0078.1;Gype08G0217.1;Gype08G1178.1;Gype08G2486.1;Gype08G2493.1;Gype08G2879.1;Gype09G1409.1;Gype02G0173.1;Gype02G0174.1;Gype02G0175.1;Gype02G0176.1;Gype02G0177.1;Gype02G0178.1;Gype01G1201.1;Gype06G0405.1;Gype06G0407.1;Gype06G0413.1;Gype06G0421.1;Gype06G0422.1;Gype06G0553.1;Gype06G0933.1;Gype07G1928.1;Gype08G2440.1;Gype11G2732.1 | K04079+K13993+K03283 |
| Ubiquinone and other terpenoid-quinone biosynthesis | 10 (0.45%) | 97 (0.46%) | 0.5920684 | 1.00E+00 | ko00130 | Gype10G0886.1;Gype10G0892.1;Gype10G0894.1;Gype10G0895.1;Gype10G0897.1;Gype10G0898.1;Gype10G0941.1;Gype07G2308.1;Gype10G0512.1;Gype11G1723.1 | K06125+K01904+K09833 |
| Pyruvate metabolism | 15 (0.67%) | 148 (0.7%) | 0.6201084 | 1.00E+00 | ko00620 | Gype03G1060.1;Gype03G1105.1;Gype04G0639.1;Gype04G0647.1;Gype04G0765.1;Gype04G0770.1;Gype04G0775.1;Gype04G0776.1;Gype10G0192.1;Gype10G0194.1;Gype10G0196.1;Gype10G0208.1;Gype10G1069.1;Gype10G1072.1;Gype10G1077.1 | K00028+K00025 |
| Fatty acid degradation | 10 (0.45%) | 100 (0.47%) | 0.6314959 | 1.00E+00 | ko00071 | Gype04G1340.1;Gype05G0299.1;Gype07G2981.1;Gype08G0659.1;Gype09G0975.1;Gype11G2173.1;Gype11G2304.1;Gype11G2391.1;Gype05G0220.1;Gype06G0676.1 | K07517 |
| Flavonoid biosynthesis | 14 (0.62%) | 146 (0.69%) | 0.7006954 | 1.00E+00 | ko00941 | Gype01G0465.1;Gype01G0472.1;Gype02G0434.1;Gype02G0507.1;Gype06G0658.1;Gype06G2280.1;Gype06G2281.1;Gype06G2283.1;Gype06G2284.1;Gype05G1946.1;Gype10G1597.1;Gype10G1599.1;Gype10G1600.1;Gype10G1601.1 | K13065+K05277+K05278 |
| Glutathione metabolism | 12 (0.54%) | 128 (0.61%) | 0.7219014 | 1.00E+00 | ko00480 | Gype06G1280.1;Gype06G1286.1;Gype06G1296.1;Gype06G1298.1;Gype06G1301.1;Gype07G0919.1;Gype08G0623.1;Gype08G0624.1;Gype02G1249.1;Gype06G1291.1;Gype06G1292.1;Gype06G1305.1 | K18592 |
| Zeatin biosynthesis | 3 (0.13%) | 35 (0.17%) | 0.7355556 | 1.00E+00 | ko00908 | Gype02G0181.1;Gype02G0183.1;Gype02G0187.1 | K13493 |
| Citrate cycle (TCA cycle) | 7 (0.31%) | 91 (0.43%) | 0.8641842 | 1.00E+00 | ko00020 | Gype10G0192.1;Gype10G0194.1;Gype10G0196.1;Gype10G0208.1;Gype10G1069.1;Gype10G1072.1;Gype10G1077.1 | K00025 |
| Peroxisome | 15 (0.67%) | 180 (0.85%) | 0.8738201 | 1.00E+00 | ko04146 | Gype06G0761.1;Gype06G0764.1;Gype06G0765.1;Gype06G0845.1;Gype06G0848.1;Gype10G1309.1;Gype10G1312.1;Gype10G1313.1;Gype10G1314.1;Gype10G1327.1;Gype10G1328.1;Gype10G1329.1;Gype10G1330.1;Gype10G1332.1;Gype05G0864.1 | K08726+K13343 |
| Glycine, serine and threonine metabolism | 9 (0.4%) | 115 (0.55%) | 0.8752701 | 1.00E+00 | ko00260 | Gype06G0761.1;Gype06G0764.1;Gype06G0845.1;Gype10G1312.1;Gype10G1313.1;Gype10G1314.1;Gype10G1328.1;Gype10G1329.1;Gype10G1330.1 | K01079 |
| mRNA surveillance pathway | 32 (1.43%) | 373 (1.77%) | 0.9215671 | 1.00E+00 | ko03015 | Gype02G0008.1;Gype04G0108.1;Gype05G0112.1;Gype05G0832.1;Gype06G0769.1;Gype06G0852.1;Gype08G0990.1;Gype08G2694.1;Gype09G1976.1;Gype09G2063.1;Gype09G2162.1;Gype04G0491.1;Gype05G0058.1;Gype05G0391.1;Gype07G3139.1;Gype10G0647.1;Gype11G2743.1;Gype02G0893.1;Gype09G2316.1;Gype11G2839.1;Gype01G0629.1;Gype03G1998.1;Gype06G0215.1;Gype09G2049.1;Gype11G1500.1;Gype02G0320.1;Gype02G0321.1;Gype02G0322.1;Gype04G1246.1;Gype05G1090.1;Gype05G1191.1;Gype11G1231.1 | K14400+K12812+K13917 |
| Ribosome biogenesis in eukaryotes | 18 (0.8%) | 231 (1.1%) | 0.9413779 | 1.00E+00 | ko03008 | Gype03G1060.1;Gype03G1105.1;Gype04G0639.1;Gype07G2552.1;Gype07G3134.1;Gype07G2308.1;Gype09G0006.1;Gype09G0024.1;Gype09G0025.1;Gype09G0111.1;Gype09G0112.1;Gype09G0114.1;Gype09G0170.1;Gype09G0172.1;Gype09G0190.1;Gype09G1361.1;Gype09G1982.1;Gype09G2001.1 | K14564+K14572+K03097+K07562+K12619 |
| Fructose and mannose metabolism | 7 (0.31%) | 108 (0.51%) | 0.9497382 | 1.00E+00 | ko00051 | Gype02G0825.1;Gype02G0826.1;Gype02G0827.1;Gype02G0829.1;Gype02G0831.1;Gype02G0833.1;Gype03G2139.1 | K00895+K03841 |
| Glycerophospholipid metabolism | 13 (0.58%) | 182 (0.86%) | 0.9580991 | 1.00E+00 | ko00564 | Gype08G1531.1;Gype08G1532.1;Gype08G1533.1;Gype08G1534.1;Gype08G1535.1;Gype08G1537.1;Gype08G1539.1;Gype08G1540.1;Gype08G1541.1;Gype04G0391.1;Gype04G0393.1;Gype04G0423.1;Gype09G2110.1 | K00993+K08730+K13510 |
| Pyrimidine metabolism | 8 (0.36%) | 126 (0.6%) | 0.9648796 | 1.00E+00 | ko00240 | Gype08G0400.1;Gype06G0330.1;Gype09G1974.1;Gype09G1975.1;Gype09G2062.1;Gype09G2064.1;Gype09G2066.1;Gype10G1835.1 | K00876+K14641 |
| Carotenoid biosynthesis | 6 (0.27%) | 103 (0.49%) | 0.9692048 | 1.00E+00 | ko00906 | Gype07G2715.1;Gype05G1805.1;Gype05G0662.1;Gype03G0041.1;Gype03G0214.1;Gype07G1067.1 | K00514+K09841+K17912 |
| Metabolic pathways | 394 (17.57%) | 4065 (19.31%) | 0.987741 | 1.00E+00 | ko01100 | Gype06G1280.1;Gype06G1286.1;Gype06G1296.1;Gype06G1298.1;Gype06G1301.1;Gype07G0919.1;Gype06G1876.1;Gype06G1883.1;Gype06G1884.1;Gype06G1888.1;Gype06G1889.1;Gype06G1890.1;Gype06G1893.1;Gype06G1896.1;Gype06G1898.1;Gype06G1899.1;Gype06G1901.1;Gype06G1903.1;Gype06G1904.1;Gype06G1905.1;Gype06G1906.1;Gype06G1908.1;Gype06G1910.1;Gype06G1914.1;Gype06G1916.1;Gype06G1917.1;Gype06G1918.1;Gype06G1919.1;Gype06G1920.1;Gype06G1922.1;Gype06G1923.1;Gype09G2055.1;Gype09G2056.1;Gype09G2057.1;Gype09G2058.1;Gype07G2715.1;Gype05G1805.1;Gype08G0563.1;Gype08G0564.1;Gype01G0468.1;Gype01G0469.1;Gype04G0304.1;Gype04G0630.1;Gype04G0632.1;Gype04G0633.1;Gype05G0369.1;Gype05G0370.1;Gype05G0371.1;Gype05G0383.1;Gype05G0385.1;Gype05G0386.1;Gype07G2591.1;Gype11G3121.1;Gype11G3123.1;Gype11G3124.1;Gype11G3126.1;Gype11G3128.1;Gype11G3130.1;Gype11G3132.1;Gype11G3133.1;Gype11G3134.1;Gype01G0409.1;Gype01G0442.1;Gype02G0430.1;Gype04G0415.1;Gype04G1340.1;Gype05G0299.1;Gype06G0610.1;Gype07G2382.1;Gype07G2899.1;Gype07G2981.1;Gype08G0609.1;Gype08G0659.1;Gype09G0975.1;Gype10G0164.1;Gype11G2173.1;Gype11G2304.1;Gype11G2391.1;Gype02G1525.1;Gype02G1527.1;Gype02G1528.1;Gype02G1529.1;Gype02G1530.1;Gype02G1531.1;Gype02G1534.1;Gype02G1535.1;Gype02G1536.1;Gype02G1537.1;Gype02G1538.1;Gype02G1539.1;Gype02G1540.1;Gype02G1541.1;Gype02G1542.1;Gype02G1543.1;Gype08G0011.1;Gype11G2798.1;Gype11G3131.1;Gype04G0363.1;Gype04G0771.1;Gype02G1081.1;Gype02G1085.1;Gype02G1086.1;Gype02G1087.1;Gype02G1089.1;Gype07G1840.1;Gype08G0809.1;Gype08G0836.1;Gype08G2721.1;Gype10G1415.1;Gype10G1428.1;Gype11G1770.1;Gype11G1771.1;Gype11G1772.1;Gype11G1774.1;Gype09G2523.1;Gype09G2526.1;Gype09G2530.1;Gype05G0662.1;Gype08G1056.1;Gype08G1057.1;Gype08G1058.1;Gype08G1060.1;Gype08G1062.1;Gype08G1066.1;Gype08G1068.1;Gype08G1072.1;Gype08G1073.1;Gype08G1075.1;Gype08G1077.1;Gype08G1080.1;Gype08G1084.1;Gype10G0826.1;Gype01G0276.1;Gype01G0760.1;Gype03G2401.1;Gype06G0761.1;Gype06G0764.1;Gype06G0765.1;Gype06G0845.1;Gype06G0848.1;Gype10G1309.1;Gype10G1312.1;Gype10G1313.1;Gype10G1314.1;Gype10G1327.1;Gype10G1328.1;Gype10G1329.1;Gype10G1330.1;Gype10G1332.1;Gype02G1493.1;Gype02G1495.1;Gype02G1496.1;Gype02G1497.1;Gype02G1498.1;Gype02G1499.1;Gype02G1606.1;Gype02G1607.1;Gype10G1375.1;Gype10G1789.1;Gype01G0309.1;Gype01G1879.1;Gype06G0184.1;Gype06G0448.1;Gype08G0995.1;Gype09G1533.1;Gype10G0829.1;Gype11G2900.1;Gype01G0977.1;Gype03G1435.1;Gype05G0043.1;Gype06G0625.1;Gype08G0432.1;Gype10G0972.1;Gype10G1397.1;Gype08G1531.1;Gype08G1532.1;Gype08G1533.1;Gype08G1534.1;Gype08G1535.1;Gype08G1537.1;Gype08G1539.1;Gype08G1540.1;Gype08G1541.1;Gype02G0825.1;Gype02G0826.1;Gype02G0827.1;Gype02G0829.1;Gype02G0831.1;Gype02G0833.1;Gype08G0400.1;Gype07G0742.1;Gype11G2588.1;Gype11G2591.1;Gype11G2749.1;Gype11G2751.1;Gype10G0886.1;Gype10G0892.1;Gype10G0894.1;Gype10G0895.1;Gype10G0897.1;Gype10G0898.1;Gype10G0941.1;Gype03G1060.1;Gype03G1105.1;Gype04G0639.1;Gype04G0647.1;Gype04G0765.1;Gype04G0770.1;Gype04G0775.1;Gype04G0776.1;Gype01G0465.1;Gype01G0472.1;Gype02G0434.1;Gype02G0507.1;Gype06G0658.1;Gype06G2280.1;Gype06G2281.1;Gype06G2283.1;Gype06G2284.1;Gype01G0807.1;Gype01G0809.1;Gype01G0812.1;Gype03G2237.1;Gype05G0216.1;Gype05G0219.1;Gype05G0327.1;Gype05G0399.1;Gype05G0400.1;Gype05G0402.1;Gype05G0409.1;Gype05G0415.1;Gype05G0416.1;Gype05G0424.1;Gype05G0426.1;Gype08G0804.1;Gype08G2835.1;Gype08G2854.1;Gype02G1432.1;Gype02G0008.1;Gype04G0108.1;Gype05G0112.1;Gype05G0832.1;Gype06G0769.1;Gype06G0852.1;Gype08G0990.1;Gype08G2694.1;Gype09G1976.1;Gype09G2063.1;Gype09G2162.1;Gype04G0524.1;Gype11G0517.1;Gype11G0518.1;Gype11G0519.1;Gype11G0521.1;Gype11G0522.1;Gype11G0557.1;Gype11G0559.1;Gype11G0562.1;Gype11G0563.1;Gype01G0369.1;Gype05G1565.1;Gype05G1567.1;Gype05G1568.1;Gype05G2051.1;Gype05G2052.1;Gype05G2053.1;Gype05G2054.1;Gype04G0391.1;Gype04G0393.1;Gype04G0423.1;Gype10G0192.1;Gype10G0194.1;Gype10G0196.1;Gype10G0208.1;Gype10G1069.1;Gype10G1072.1;Gype10G1077.1;Gype01G1610.1;Gype09G2221.1;Gype11G1111.1;Gype03G1515.1;Gype04G0207.1;Gype04G0208.1;Gype04G0209.1;Gype04G0210.1;Gype04G0211.1;Gype04G0212.1;Gype05G0606.1;Gype05G0607.1;Gype07G0280.1;Gype07G0281.1;Gype07G1052.1;Gype08G0428.1;Gype09G2210.1;Gype09G2332.1;Gype11G0164.1;Gype01G0629.1;Gype03G1998.1;Gype06G0215.1;Gype06G0701.1;Gype09G2015.1;Gype09G2049.1;Gype11G1500.1;Gype01G0500.1;Gype07G2271.1;Gype08G0098.1;Gype08G0099.1;Gype08G0101.1;Gype08G0103.1;Gype08G0104.1;Gype08G0105.1;Gype08G0106.1;Gype08G0108.1;Gype08G0109.1;Gype08G0115.1;Gype08G0116.1;Gype08G0117.1;Gype08G0118.1;Gype08G0119.1;Gype08G0120.1;Gype08G0121.1;Gype03G2139.1;Gype07G0020.1;Gype10G0326.1;Gype11G2840.1;Gype07G2308.1;Gype10G0512.1;Gype11G1723.1;Gype02G0953.1;Gype02G0954.1;Gype02G0955.1;Gype02G0956.1;Gype02G0957.1;Gype02G0959.1;Gype02G0960.1;Gype02G0963.1;Gype02G0968.1;Gype02G0981.1;Gype02G0983.1;Gype08G0623.1;Gype08G0624.1;Gype02G1526.1;Gype02G1532.1;Gype02G1533.1;Gype07G1201.1;Gype09G2110.1;Gype04G0929.1;Gype07G2265.1;Gype07G2269.1;Gype02G1249.1;Gype06G1291.1;Gype06G1292.1;Gype01G0563.1;Gype01G0564.1;Gype01G0565.1;Gype01G0566.1;Gype01G0567.1;Gype01G0642.1;Gype01G0644.1;Gype01G0655.1;Gype01G0656.1;Gype01G0546.1;Gype02G0635.1;Gype02G0637.1;Gype02G0639.1;Gype02G0640.1;Gype02G0641.1;Gype02G0644.1;Gype02G0645.1;Gype02G0646.1;Gype05G0305.1;Gype09G1284.1;Gype10G1851.1;Gype10G1853.1;Gype10G1854.1;Gype10G1855.1;Gype10G1856.1;Gype10G1857.1;Gype10G1858.1;Gype10G1860.1;Gype05G1946.1;Gype06G1305.1;Gype10G1597.1;Gype10G1599.1;Gype10G1600.1;Gype10G1601.1;Gype05G0858.1;Gype09G0441.1;Gype11G0520.1;Gype11G0558.1;Gype11G0560.1;Gype02G0181.1;Gype02G0183.1;Gype02G0187.1 | K18592+K01193+K00514+K09841+K10781+K07385+K18108+K01807+K00512+K01051+K08912+K00423+K01593+K08726+K01079+K00993+K00895+K00876+K04123+K06125+K00028+K13065+K22395+K00430+K12448+K00134+K00811+K00454+K05350+K08730+K00025+K13484+K01188+K01229+K03841+K01213+K01904+K09833+K13510+K05282+K05359+K20623+K05277+K05278+K04628 |
| Purine metabolism | 10 (0.45%) | 171 (0.81%) | 0.9899885 | 1.00E+00 | ko00230 | Gype01G1610.1;Gype09G2221.1;Gype11G1111.1;Gype06G0330.1;Gype09G1974.1;Gype09G1975.1;Gype09G2062.1;Gype09G2064.1;Gype09G2066.1;Gype10G1835.1 | K13484+K14641 |
| Amino sugar and nucleotide sugar metabolism | 16 (0.71%) | 249 (1.18%) | 0.9921766 | 1.00E+00 | ko00520 | Gype02G0008.1;Gype04G0108.1;Gype05G0112.1;Gype05G0832.1;Gype06G0769.1;Gype06G0852.1;Gype08G0990.1;Gype09G1976.1;Gype09G2063.1;Gype09G2162.1;Gype01G0629.1;Gype03G1998.1;Gype06G0215.1;Gype06G0701.1;Gype09G2049.1;Gype11G1500.1 | K12448 |
| Terpenoid backbone biosynthesis | 4 (0.18%) | 96 (0.46%) | 0.9936388 | 1.00E+00 | ko00900 | Gype05G0369.1;Gype11G3120.1;Gype11G3121.1;Gype11G3126.1 | K12742 |
| Glyoxylate and dicarboxylate metabolism | 7 (0.31%) | 144 (0.68%) | 0.9957422 | 1.00E+00 | ko00630 | Gype10G0192.1;Gype10G0194.1;Gype10G0196.1;Gype10G0208.1;Gype10G1069.1;Gype10G1072.1;Gype10G1077.1 | K00025 |
| Tropane, piperidine and pyridine alkaloid biosynthesis | 1 (0.04%) | 53 (0.25%) | 0.9974602 | 1.00E+00 | ko00960 | Gype04G0524.1 | K00811 |
| Ascorbate and aldarate metabolism | 3 (0.13%) | 91 (0.43%) | 0.9975531 | 1.00E+00 | ko00053 | Gype09G2523.1;Gype09G2526.1;Gype09G2530.1 | K00423 |
| Fatty acid biosynthesis | 2 (0.09%) | 75 (0.36%) | 0.9978895 | 1.00E+00 | ko00061 | Gype08G0563.1;Gype08G0564.1 | K10781 |
| Proteasome | 3 (0.13%) | 97 (0.46%) | 0.9986041 | 1.00E+00 | ko03050 | Gype07G0922.1;Gype10G1598.1;Gype09G2089.1 | K03062+K02732 |
| Arginine biosynthesis | 1 (0.04%) | 65 (0.31%) | 0.999345 | 1.00E+00 | ko00220 | Gype04G0524.1 | K00811 |
| Sphingolipid metabolism | 3 (0.13%) | 106 (0.5%) | 0.9994058 | 1.00E+00 | ko00600 | Gype02G0181.1;Gype02G0183.1;Gype02G0187.1 | K04628 |
| Cysteine and methionine metabolism | 8 (0.36%) | 191 (0.91%) | 0.9996499 | 1.00E+00 | ko00270 | Gype04G0524.1;Gype10G0192.1;Gype10G0194.1;Gype10G0196.1;Gype10G0208.1;Gype10G1069.1;Gype10G1072.1;Gype10G1077.1 | K00811+K00025 |
| Protein export | 1 (0.04%) | 74 (0.35%) | 0.9997631 | 1.00E+00 | ko03060 | Gype08G0913.1 | K12947 |
| Alanine, aspartate and glutamate metabolism | 1 (0.04%) | 74 (0.35%) | 0.9997631 | 1.00E+00 | ko00250 | Gype04G0524.1 | K00811 |
| ABC transporters | 4 (0.18%) | 140 (0.66%) | 0.9998744 | 1.00E+00 | ko02010 | Gype10G1597.1;Gype10G1598.1;Gype10G1599.1;Gype10G1600.1 | K05658 |
| Homologous recombination | 3 (0.13%) | 140 (0.66%) | 0.9999787 | 1.00E+00 | ko03440 | Gype10G0866.1;Gype09G2271.1;Gype09G2284.1 | K10683 |
| Fatty acid metabolism | 2 (0.09%) | 128 (0.61%) | 0.9999914 | 1.00E+00 | ko01212 | Gype08G0563.1;Gype08G0564.1 | K10781 |
| Circadian rhythm - plant | 4 (0.18%) | 182 (0.86%) | 0.9999977 | 1.00E+00 | ko04712 | Gype09G1971.1;Gype09G2007.1;Gype09G2019.1;Gype07G2308.1 | K12125+K03097 |
| Ubiquitin mediated proteolysis | 9 (0.4%) | 272 (1.29%) | 0.9999984 | 1.00E+00 | ko04120 | Gype05G1575.1;Gype05G1577.1;Gype05G1579.1;Gype05G1580.1;Gype05G1581.1;Gype05G2042.1;Gype05G2043.1;Gype05G2044.1;Gype05G2046.1 | K04506 |
| Plant hormone signal transduction | 38 (1.69%) | 677 (3.22%) | 0.999999 | 1.00E+00 | ko04075 | Gype10G0518.1;Gype10G0521.1;Gype10G0524.1;Gype10G0526.1;Gype10G0529.1;Gype10G0534.1;Gype10G0535.1;Gype10G0536.1;Gype10G0537.1;Gype10G0538.1;Gype10G0539.1;Gype10G0540.1;Gype10G0541.1;Gype10G0542.1;Gype11G2244.1;Gype11G2246.1;Gype11G2358.1;Gype11G2360.1;Gype09G2164.1;Gype10G0517.1;Gype10G0519.1;Gype10G0520.1;Gype10G0522.1;Gype10G0523.1;Gype10G0525.1;Gype10G0527.1;Gype10G0528.1;Gype10G0530.1;Gype10G0532.1;Gype10G0533.1;Gype01G0172.1;Gype01G0173.1;Gype01G0174.1;Gype01G0175.1;Gype01G0176.1;Gype01G0177.1;Gype03G2177.1;Gype03G2181.1 | K14488+K14432+K14491+K13415+K13416 |
| RNA transport | 22 (0.98%) | 485 (2.3%) | 0.9999997 | 1.00E+00 | ko03013 | Gype07G2642.1;Gype08G0921.1;Gype04G0491.1;Gype05G0058.1;Gype05G0391.1;Gype07G3139.1;Gype10G0647.1;Gype11G2743.1;Gype02G0893.1;Gype09G2316.1;Gype11G2839.1;Gype07G2641.1;Gype09G0006.1;Gype09G0024.1;Gype09G0025.1;Gype09G0111.1;Gype09G0112.1;Gype09G0114.1;Gype09G0170.1;Gype09G0172.1;Gype09G0190.1;Gype09G1361.1 | K05749+K14301+K12812+K07562 |
| Spliceosome | 10 (0.45%) | 373 (1.77%) | 1 | 1.00E+00 | ko03040 | Gype04G0491.1;Gype05G0058.1;Gype05G0391.1;Gype07G3139.1;Gype10G0647.1;Gype11G2743.1;Gype02G0893.1;Gype09G2316.1;Gype11G2839.1;Gype01G1201.1 | K12812+K03283 |
| RNA degradation | 7 (0.31%) | 368 (1.75%) | 1 | 1.00E+00 | ko03018 | Gype05G0864.1;Gype06G1961.1;Gype09G2091.1;Gype03G2139.1;Gype09G1705.1;Gype09G1982.1;Gype09G2001.1 | K00962+K12602+K12606+K12619 |
| Ribosome | 5 (0.22%) | 471 (2.24%) | 1 | 1.00E+00 | ko03010 | Gype11G2952.1;Gype03G0041.1;Gype03G0214.1;Gype07G1067.1;Gype02G1117.1 | K02897+K02895+K02912 |
| Endocytosis | 2 (0.09%) | 326 (1.55%) | 1 | 1.00E+00 | ko04144 | Gype10G1081.1;Gype01G1201.1 | K18466+K03283 |
| Aminoacyl-tRNA biosynthesis | 1 (0.04%) | 164 (0.78%) | 1 | 1.00E+00 | ko00970 | Gype09G2683.1 | K01881 |

**Table S13. KEGG pathway enrichment analysis of the contracted gene families in G. pentaphyllum.**

| **Pathway** | **DEGs with pathway annotation (16)** | **All genes with pathway annotation (21053)** | **Pvalue** | **Qvalue** | **Pathway ID** | **Genes** | **KOs** |
| --- | --- | --- | --- | --- | --- | --- | --- |
| Benzoxazinoid biosynthesis | 2 (12.5%) | 8 (0.04%) | 1.51E-05 | 0.000105853 | ko00402 | Gype03G0356.1;Gype06G3041.1 | K13229 |
| Phagosome | 3 (18.75%) | 115 (0.55%) | 8.44E-05 | 0.00029547 | ko04145 | Gype02G1716.1;Gype05G0933.1;Gype05G1195.1 | K07375 |
| Phenylpropanoid biosynthesis | 2 (12.5%) | 462 (2.19%) | 0.04707206 | 0.109834807 | ko00940 | Gype03G0356.1;Gype06G3041.1 | K06892 |
| Tryptophan metabolism | 1 (6.25%) | 146 (0.69%) | 0.1054052 | 0.1844591 | ko00380 | Gype10G0455.1 | K22588 |
| Endocytosis | 1 (6.25%) | 326 (1.55%) | 0.2210307 | 0.30944298 | ko04144 | Gype06G0672.1 | K07937 |
| Biosynthesis of secondary metabolites | 2 (12.5%) | 2,383 (11.32%) | 0.554967 | 0.6474615 | ko01110 | Gype03G0356.1;Gype06G3041.1 | K06892 |
| Metabolic pathways | 1 (6.25%) | 4,065 (19.31%) | 0.9677404 | 0.9677404 | ko01100 | Gype10G0455.1 | K22588 |

**Table S14. Statistics results of transcriptome sequencing clean data of G. pentaphyllum.**

| **No.** | **Sample Name** | **Clean Reads** | **Clean bases (bp)** | **Read length (bp)** | **Q20(%)** | **GC(%)** |
| --- | --- | --- | --- | --- | --- | --- |
| 1 | Fruit | 39,828,320 | 5,974,248,000 | 150 | 93.12 | 44.93 |
| 2 | Leaf1 | 40,462,746 | 6,069,411,900 | 150 | 97.82 | 42.35 |
| 3 | Leaf2 | 41,185,410 | 6,177,811,500 | 150 | 97.90 | 42.39 |
| 4 | Leaf3 | 41,085,964 | 6,162,894,600 | 150 | 97.71 | 41.41 |
| 5 | Leaf4 | 41,300,710 | 6,195,106,500 | 150 | 97.76 | 42.03 |
| 6 | Flower1 | 41,140,318 | 6,171,047,700 | 150 | 97.58 | 40.87 |
| 7 | Flower2 | 40,150,056 | 6,022,508,400 | 150 | 97.71 | 41.19 |
| 8 | Flower3 | 41,186,930 | 6,178,039,500 | 150 | 97.51 | 40.85 |
| 9 | Flower4 | 41,393,240 | 6,208,986,000 | 150 | 97.72 | 39.76 |
| 10 | Tendril1 | 41,521,322 | 6,228,198,300 | 150 | 93.26 | 41.94 |
| 11 | Tendril2 | 46,335,842 | 6,950,376,300 | 150 | 94.89 | 39.51 |
| 12 | Tendril3 | 39,263,480 | 5,889,522,000 | 150 | 93.94 | 39.64 |
| 13 | Stem1 | 41,353,272 | 6,202,990,800 | 150 | 97.45 | 41.34 |
| 14 | Stem2 | 41,188,456 | 6,178,268,400 | 150 | 97.57 | 41.39 |
| 15 | Stem3 | 41,473,746 | 6,221,061,900 | 150 | 97.60 | 40.81 |
| 16 | Stem4 | 40,218,916 | 6,032,837,400 | 150 | 97.58 | 42.06 |
| Average |  | 41,193,046 | 6,178,956,825 | 150 | 96.70 | 41.40 |

**Table S15. The expression values of DEGs involved in gypenoside biosynthesis in G. pentaphyllum.**

| **Genes** | **Fruit** | **Flower** | **Stem** | **Leaf** | **Tendril** |
| --- | --- | --- | --- | --- | --- |
| *GPPS1-Gype08G1924* | 0.109192 | 1.377574 | 1.78468975 | 1.81547025 | 5.303916667 |
| *FPS1-Gype02G0716* | 9.755571 | 19.92877175 | 27.43496025 | 25.11123775 | 18.15757167 |
| *FPS2-Gype09G1877* | 70.459084 | 15.60674225 | 16.9100815 | 6.353917 | 20.514095 |
| *SS1-Gype11G2380* | 0.031964 | 0.02456575 | 0.03163325 | 0.02963125 | 0.022468 |
| *SE1-Gype11G0860* | 0 | 0.02162125 | 0.0079665 | 0 | 0.019970667 |
| *SE2-Gype11G0861* | 0 | 0.17317775 | 0 | 0 | 0.075532 |
| *OSC1-Gype07G1532* | 0 | 0.03549175 | 0.01220225 | 0.04696375 | 0.063684 |
| *OSC2-Gype07G1533* | 0 | 0.017048 | 0.03389 | 0.0176825 | 0.023383333 |
| *OSC3-Gype07G1534* | 0 | 0.0132365 | 0.05907675 | 0.00218375 | 0.009416333 |
| *OSC4-Gype07G1535* | 0 | 0.39005725 | 0.2957775 | 0.29220825 | 0.310287 |
| *CYP4501-Gype03G1089* | 0 | 0.3731715 | 0.228275 | 0.038519 | 0.01014 |
| *CYP4502-Gype11G1710* | 226.999512 | 108.6234608 | 317.6087113 | 76.09181 | 297.0276787 |
| *CYP4503-Gype06G3206* | 1.287047 | 7.36576725 | 140.417101 | 0.88127525 | 45.94140067 |
| *CYP4504-Gype07G2333* | 0.053831 | 0.04879225 | 0.1376435 | 0.008784 | 0.359351 |
| *CYP4505-Gype06G0596* | 0.23236 | 1.94549775 | 0.02905675 | 0.00051375 | 0.814070667 |
| *CYP4506-Gype07G2334* | 0 | 0.0071545 | 0 | 0 | 0.089377333 |
| *CYP4507-Gype09G0595* | 21.93367 | 2.272051 | 3.77243625 | 11.89361175 | 0 |
| *CYP4508-Gype09G0590* | 6.238155 | 3.82494325 | 2.780898 | 1.58931875 | 1.151501333 |
| *GT1-Gype04G0348* | 0.403933 | 0 | 0 | 0 | 0.183092333 |
| *GT2-Gype06G0006* | 0.356762 | 0.242377 | 0.09477675 | 0 | 0.502402 |
| *GT3-Gype09G2040* | 0.09786 | 0.11894725 | 0 | 0.02062425 | 0.433224333 |
| *GT4-Gype11G0465* | 0 | 0 | 0 | 0 | 0.352616667 |
| *GT5-Gype07G1454* | 61.871567 | 13.894798 | 31.3360825 | 135.4086763 | 28.32788767 |
| *GT6-Gype08G0431* | 0 | 0 | 0 | 0 | 0.140571 |
| *GT7-Gype04G0329* | 0 | 0.01613225 | 0.0114105 | 0.00666225 | 0.042151667 |
| *GT8-Gype05G1407* | 0.229347 | 0.108703 | 0.206808 | 0.04070775 | 0.410668 |
| *GT9-Gype05G1395* | 0.391283 | 0.26373875 | 0.2054135 | 0.01052375 | 0.32076 |
| *GT10-Gype09G1323* | 0 | 0 | 0 | 0 | 0.376377 |
| *GT11-Gype07G0867* | 59.56683 | 0 | 0 | 0 | 5.626754333 |
| *GT12-Gype04G0133* | 4.74962 | 1.17650625 | 0.2463995 | 3.6837235 | 1.548037333 |
| *GT13-Gype06G0339* | 0.543069 | 0.240949 | 0.06309375 | 0.07983475 | 1.663230667 |
| *GT14-Gype01G0039* | 63.704586 | 8.21951475 | 35.3910235 | 18.18508175 | 78.133331 |
| *GT15-Gype04G1425* | 9.976031 | 1.4003035 | 2.99191275 | 6.73220175 | 5.964036 |
| *GT16-Gype11G1924* | 1.766201 | 185.9466133 | 145.615633 | 22.67563225 | 65.36321933 |
| *GT17-Gype11G0869* | 0 | 0.515961 | 8.90185475 | 0.5579835 | 6.103945333 |
| *GT18-Gype04G0335* | 0.124971 | 0.0228545 | 0 | 0 | 0 |
| *AC1-Gype09G2675* | 13.267912 | 7.17520025 | 5.05756725 | 31.24924775 | 1.806984333 |
| *AC2-Gype07G2288* | 2.486948 | 2.0870785 | 3.57157625 | 1.91601425 | 2.906591333 |
| *AC3-Gype10G0618* | 0.506456 | 11.724091 | 9.501963 | 8.411455 | 0.086500333 |
| *AC4-Gype06G2148* | 1.698283 | 1.0835545 | 0.64658375 | 1.15802175 | 1.145490667 |
| *AC5-Gype06G2218* | 1.378495 | 1.60476825 | 1.592097 | 7.16649725 | 0.224528 |
| *AC6-Gype10G0584* | 0.169305 | 0.40761925 | 0.0252055 | 0.02718325 | 0.424139333 |
| *AC7-Gype08G2287* | 0.231607 | 0.28511225 | 0.070072 | 0.2908665 | 0.069121 |
| *AC8-Gype06G0658* | 0.001 | 0.0557425 | 0.08845225 | 0 | 0.367854 |
| *AC9-Gype02G0808* | 0.001 | 2.7146005 | 0.2649895 | 0 | 0.070105 |
| *AC10-Gype05G1951* | 9.000292 | 0 | 0.000075 | 0.0000505 | 5.556231333 |
| *AC11-Gype02G0856* | 0.001 | 0.14511775 | 1.4571265 | 0 | 0.235013333 |
| *AC12-Gype11G1352* | 0.808809 | 0.5577285 | 1.519726 | 4.0670445 | 0.133637333 |
| *AC13-Gype08G0863* | 2.341494 | 0.02211875 | 0.0306695 | 0.0377405 | 0.732489333 |
| *AC14-Gype06G1106* | 0.161949 | 0.21666275 | 0.08884175 | 0.0394365 | 0.574915333 |
| *AC15-Gype06G3122* | 0.683012 | 0 | 1.20618325 | 0 | 0.701557 |
| *AC16-Gype10G1848* | 0.096037 | 1.507535 | 0.21312 | 0.020186 | 0.666775667 |
| *AC17-Gype05G0613* | 0.001 | 0.0556015 | 0.00075 | 0 | 0.391897 |
| *AC18-Gype11G0727* | 0.537692 | 0.21381225 | 3.25739725 | 0.1133865 | 0.270406 |
| *AC19-Gype07G0838* | 7.857669 | 4.405988 | 5.38731875 | 9.35137625 | 0.455610333 |
| *AC20-Gype10G0687* | 14.677992 | 7.820767 | 10.02601575 | 14.286659 | 18.78938967 |
| *AC21-Gype10G0140* | 0.001 | 0 | 0.326397 | 0.17461175 | 0.134707 |
| *AC22-Gype04G0973* | 0.895111 | 0.74403075 | 1.302265 | 0.22477625 | 0 |

**Table S16. Statistics of GBS sequencing reads and results of genetic diversity of G. pentaphyllum.**

| **No.** | **Sample** | **Raw reads (bp)** | **Clean reads (bp)** | **Effective Rate(%)** | **Error Rate(%)** | **Q20(%)** | **Q30(%)** | **GC Content (%)** | **O(HOM)** | **E(HOM)** | **N(NM)** | **F** | **Ho** | **He** |
| --- | --- | --- | --- | --- | --- | --- | --- | --- | --- | --- | --- | --- | --- | --- |
| 1 | **WS1** | 460,856,736 | 460,831,392 | 99.99 | 0.03 | 96.32 | 90.57 | 37.24 | 21,396 | 19,580 | 24,296 | 0.3846 | 0.1194 | 0.1941 |
| 2 | **WS2** | 370,979,136 | 370,963,296 | 100.00 | 0.03 | 95.77 | 89.17 | 37.36 | 21,060 | 19,210 | 23,841 | 0.3999 | 0.1166 | 0.1942 |
| 3 | **WS3** | 413,873,568 | 413,850,816 | 99.99 | 0.03 | 96.46 | 91.00 | 36.86 | 21,323 | 19,320 | 24,004 | 0.4273 | 0.1117 | 0.1951 |
| 4 | **XC1** | 453,557,952 | 453,557,952 | 100.00 | 0.03 | 95.99 | 89.99 | 37.00 | 20,706 | 19,710 | 24,447 | 0.2106 | 0.1530 | 0.1938 |
| 5 | **XC2** | 478,130,112 | 478,130,112 | 100.00 | 0.03 | 96.29 | 90.77 | 37.02 | 20,874 | 19,860 | 24,623 | 0.2124 | 0.1523 | 0.1934 |
| 6 | **XC3** | 440,577,504 | 440,559,936 | 100.00 | 0.03 | 95.78 | 89.42 | 35.84 | 20,327 | 19,190 | 23,800 | 0.2466 | 0.1459 | 0.1937 |
| 7 | **XC4** | 425,003,904 | 424,979,424 | 99.99 | 0.03 | 96.18 | 90.23 | 36.52 | 20,790 | 19,630 | 24,359 | 0.2449 | 0.1465 | 0.1941 |
| 8 | **XC5** | 434,241,792 | 434,184,192 | 99.99 | 0.05 | 94.13 | 85.00 | 36.84 | 20,567 | 19,320 | 23,971 | 0.2674 | 0.1420 | 0.1940 |
| 9 | **ST1** | 480,956,256 | 480,876,192 | 99.98 | 0.04 | 95.01 | 87.75 | 37.16 | 21,380 | 19,770 | 24,538 | 0.3383 | 0.1287 | 0.1943 |
| 10 | **ST2** | 527,362,848 | 527,362,848 | 100.00 | 0.03 | 96.18 | 90.55 | 35.67 | 21,505 | 19,830 | 24,597 | 0.3508 | 0.1257 | 0.1938 |
| 11 | **ST3** | 470,344,896 | 470,344,896 | 100.00 | 0.03 | 95.97 | 89.90 | 37.29 | 21,540 | 19,780 | 24,537 | 0.3700 | 0.1221 | 0.1939 |
| 12 | **ST4** | 439,302,816 | 439,302,816 | 100.00 | 0.04 | 95.09 | 87.84 | 37.58 | 21,553 | 19,680 | 24,423 | 0.3951 | 0.1175 | 0.1942 |
| 13 | **ST5** | 499,235,040 | 499,235,040 | 100.00 | 0.03 | 96.43 | 91.09 | 37.48 | 21,633 | 19,920 | 24,716 | 0.3566 | 0.1247 | 0.1940 |
| 14 | **WN1** | 443,117,952 | 443,094,912 | 99.99 | 0.03 | 96.50 | 90.90 | 36.66 | 21,274 | 20,250 | 25,114 | 0.2109 | 0.1529 | 0.1937 |
| 15 | **WN2** | 404,161,632 | 404,143,776 | 100.00 | 0.03 | 96.26 | 90.21 | 37.41 | 21,112 | 19,730 | 24,469 | 0.2915 | 0.1372 | 0.1937 |
| 16 | **WN3** | 442,958,400 | 442,929,888 | 99.99 | 0.03 | 96.62 | 91.23 | 37.07 | 21,601 | 21,040 | 26,083 | 0.1118 | 0.1718 | 0.1933 |
| 17 | **WN4** | 417,295,584 | 417,272,832 | 99.99 | 0.03 | 96.67 | 91.31 | 37.05 | 21,354 | 20,480 | 25,390 | 0.1781 | 0.1590 | 0.1934 |
| 18 | **WN5** | 444,068,352 | 444,045,600 | 99.99 | 0.03 | 96.36 | 90.48 | 36.41 | 21,386 | 20,450 | 25,353 | 0.1912 | 0.1565 | 0.1934 |
| 19 | **XY1** | 452,873,664 | 452,844,000 | 99.99 | 0.03 | 96.68 | 91.40 | 37.11 | 21,771 | 20,250 | 25,122 | 0.3118 | 0.1334 | 0.1939 |
| 20 | **XY2** | 414,791,136 | 414,771,264 | 100.00 | 0.03 | 95.59 | 88.70 | 36.96 | 21,622 | 19,900 | 24,693 | 0.3589 | 0.1244 | 0.1941 |
| 21 | **XY3** | 475,037,280 | 475,009,056 | 99.99 | 0.03 | 96.69 | 91.49 | 36.88 | 21,738 | 20,270 | 25,136 | 0.3018 | 0.1352 | 0.1936 |
| 22 | **HB1** | 387,628,704 | 387,602,784 | 99.99 | 0.03 | 96.31 | 90.63 | 37.67 | 21,129 | 18,940 | 23,498 | 0.4801 | 0.1008 | 0.1940 |
| 23 | **HB2** | 415,162,368 | 415,139,328 | 99.99 | 0.03 | 96.17 | 90.27 | 37.25 | 21,199 | 18,920 | 23,494 | 0.4981 | 0.0977 | 0.1947 |
| 24 | **YF1** | 605,297,376 | 605,297,376 | 100.00 | 0.03 | 95.62 | 89.25 | 36.22 | 21,211 | 19,810 | 24,544 | 0.2960 | 0.1358 | 0.1929 |
| 25 | **YF2** | 368,295,840 | 368,254,080 | 99.99 | 0.05 | 94.14 | 85.00 | 37.09 | 20,096 | 18,340 | 22,752 | 0.3983 | 0.1167 | 0.1939 |
| 26 | **YF3** | 542,827,008 | 542,827,008 | 100.00 | 0.03 | 96.02 | 90.05 | 36.96 | 21,484 | 19,950 | 24,724 | 0.3214 | 0.1310 | 0.1931 |
| 27 | **YF4** | 563,901,120 | 563,901,120 | 100.00 | 0.03 | 95.67 | 89.27 | 36.56 | 21,372 | 19,940 | 24,719 | 0.2991 | 0.1354 | 0.1933 |
| 28 | **YF5** | 556,832,160 | 556,832,160 | 100.00 | 0.03 | 96.02 | 90.09 | 36.98 | 21,482 | 20,050 | 24,836 | 0.2998 | 0.1350 | 0.1927 |
| 29 | **RH1** | 500,331,744 | 500,251,104 | 99.98 | 0.04 | 94.37 | 86.21 | 37.02 | 21,356 | 20,230 | 25,081 | 0.2326 | 0.1485 | 0.1934 |
| 30 | **RH2** | 482,711,040 | 482,643,936 | 99.99 | 0.05 | 93.87 | 85.00 | 36.48 | 21,276 | 20,010 | 24,822 | 0.2630 | 0.1429 | 0.1939 |
| 31 | **RH3** | 420,359,616 | 420,314,400 | 99.99 | 0.04 | 93.81 | 85.00 | 35.79 | 20,358 | 18,850 | 23,414 | 0.3302 | 0.1305 | 0.1949 |
| 32 | **RH4** | 453,933,216 | 453,874,464 | 99.99 | 0.05 | 94.03 | 85.00 | 37.08 | 21,154 | 19,910 | 24,717 | 0.2582 | 0.1442 | 0.1945 |
| 33 | **RH5** | 412,482,240 | 412,457,472 | 99.99 | 0.03 | 96.12 | 90.14 | 36.59 | 21,151 | 19,840 | 24,614 | 0.2753 | 0.1407 | 0.1940 |
| 34 | **ZT1** | 479,401,056 | 479,327,616 | 99.98 | 0.04 | 94.13 | 85.79 | 35.84 | 20,833 | 18,420 | 22,843 | 0.5457 | 0.0880 | 0.1936 |
| 35 | **ZT2** | 410,933,664 | 410,887,872 | 99.99 | 0.04 | 93.83 | 85.00 | 37.26 | 21,339 | 19,160 | 23,753 | 0.4745 | 0.1016 | 0.1934 |
| 36 | **ZT3** | 411,041,952 | 411,024,384 | 100.00 | 0.04 | 95.27 | 88.05 | 36.14 | 21,622 | 19,290 | 23,922 | 0.5036 | 0.0961 | 0.1936 |
| 37 | **ZT4** | 480,321,216 | 480,253,248 | 99.99 | 0.04 | 93.93 | 85.41 | 35.12 | 20,500 | 18,440 | 22,854 | 0.4673 | 0.1030 | 0.1931 |
| 38 | **ZT5** | 388,650,816 | 388,635,840 | 100.00 | 0.03 | 95.77 | 89.27 | 37.09 | 21,305 | 19,070 | 23,652 | 0.4873 | 0.0992 | 0.1937 |
| 39 | **JY1** | 474,524,352 | 474,494,400 | 99.99 | 0.03 | 96.14 | 90.14 | 35.38 | 21,798 | 21,280 | 26,395 | 0.1014 | 0.1742 | 0.1938 |
| 40 | **JY2** | 500,392,800 | 500,360,832 | 99.99 | 0.03 | 96.41 | 90.85 | 35.56 | 21,837 | 21,480 | 26,660 | 0.0681 | 0.1809 | 0.1943 |
| 41 | **JY3** | 493,011,936 | 492,981,984 | 99.99 | 0.03 | 96.39 | 90.72 | 34.86 | 21,876 | 21,170 | 26,265 | 0.1381 | 0.1671 | 0.1940 |
| 42 | **JS1** | 404,286,048 | 404,231,040 | 99.99 | 0.04 | 93.89 | 85.00 | 37.12 | 21,266 | 21,360 | 26,532 | -0.0186 | 0.1985 | 0.1949 |
| 43 | **JS2** | 277,566,912 | 277,528,032 | 99.99 | 0.05 | 94.16 | 85.00 | 36.77 | 20,328 | 19,190 | 23,861 | 0.2437 | 0.1481 | 0.1958 |
| 44 | **ES1** | 1,245,795,840 | 1,245,711,744 | 99.99 | 0.04 | 94.59 | 87.32 | 36.81 | 20,408 | 20,320 | 25,212 | 0.0189 | 0.1905 | 0.1940 |
| 45 | **ES2** | 399,387,168 | 399,369,600 | 100.00 | 0.03 | 96.34 | 90.67 | 36.66 | 20,481 | 20,800 | 25,804 | -0.0627 | 0.2063 | 0.1939 |
| 46 | **ES3** | 497,474,784 | 497,407,968 | 99.99 | 0.05 | 93.86 | 85.20 | 36.66 | 21,091 | 23,190 | 28,739 | -0.3780 | 0.2661 | 0.1931 |
| 47 | **HS1** | 422,323,488 | 422,258,112 | 99.98 | 0.04 | 94.75 | 87.07 | 37.25 | 20,102 | 20,990 | 26,034 | -0.1749 | 0.2279 | 0.1937 |
| 48 | **HS2** | 767,150,784 | 767,150,784 | 100.00 | 0.04 | 93.92 | 85.00 | 36.49 | 20,000 | 20,930 | 25,971 | -0.1836 | 0.2299 | 0.1941 |
| 49 | **HS3** | 430,056,000 | 430,030,656 | 99.99 | 0.03 | 96.36 | 90.62 | 36.84 | 19,890 | 19,870 | 24,664 | 0.0049 | 0.1936 | 0.1944 |
| 50 | **HS4** | 476,476,128 | 476,452,800 | 100.00 | 0.03 | 96.61 | 91.16 | 36.73 | 20,071 | 20,600 | 25,544 | -0.1070 | 0.2143 | 0.1935 |
| 51 | **HS5** | 425,984,832 | 425,957,760 | 99.99 | 0.03 | 96.54 | 91.19 | 35.22 | 19,560 | 19,830 | 24,624 | -0.0571 | 0.2057 | 0.1947 |
| 52 | **ZZ1** | 458,417,088 | 458,356,320 | 99.99 | 0.04 | 94.57 | 86.62 | 36.94 | 19,745 | 19,640 | 24,398 | 0.0213 | 0.1907 | 0.1950 |
| 53 | **SX1** | 272,745,504 | 272,701,728 | 99.98 | 0.04 | 94.06 | 85.88 | 35.49 | 17,747 | 17,350 | 21,568 | 0.0945 | 0.1772 | 0.1956 |
| 54 | **SX2** | 358,914,528 | 358,900,128 | 100.00 | 0.03 | 96.13 | 90.07 | 37.10 | 18,990 | 19,090 | 23,714 | -0.0207 | 0.1992 | 0.1950 |
| 55 | **SX3** | 356,875,200 | 356,860,512 | 100.00 | 0.04 | 95.28 | 88.10 | 36.97 | 18,851 | 18,950 | 23,537 | -0.0220 | 0.1991 | 0.1949 |
| 56 | **SX4** | 414,517,248 | 414,517,248 | 100.00 | 0.03 | 96.30 | 90.70 | 36.93 | 19,402 | 19,930 | 24,752 | -0.1104 | 0.2161 | 0.1948 |
| 57 | **DY1** | 353,394,432 | 353,376,576 | 99.99 | 0.03 | 95.72 | 89.00 | 37.03 | 19,044 | 19,180 | 23,804 | -0.0286 | 0.2000 | 0.1943 |
| 58 | **DY2** | 443,580,192 | 443,557,152 | 99.99 | 0.03 | 96.81 | 91.72 | 37.08 | 19,465 | 20,070 | 24,890 | -0.1257 | 0.2180 | 0.1937 |
| 59 | **DY3** | 409,733,856 | 409,714,272 | 100.00 | 0.03 | 96.73 | 91.44 | 36.91 | 19,478 | 19,890 | 24,681 | -0.0856 | 0.2108 | 0.1941 |
| 60 | **LZ1** | 385,335,072 | 385,314,912 | 99.99 | 0.03 | 95.72 | 89.09 | 37.27 | 19,259 | 19,270 | 23,927 | -0.0021 | 0.1951 | 0.1946 |
| 61 | **LZ2** | 506,088,288 | 506,008,800 | 99.98 | 0.04 | 94.78 | 87.28 | 36.85 | 19,510 | 20,230 | 25,113 | -0.1468 | 0.2231 | 0.1944 |
| 62 | **LZ3** | 340,211,232 | 340,198,560 | 100.00 | 0.03 | 95.28 | 88.09 | 36.99 | 18,762 | 18,690 | 23,216 | 0.0161 | 0.1919 | 0.1950 |
| 63 | **LZ4** | 443,556,000 | 443,535,840 | 100.00 | 0.03 | 96.12 | 90.06 | 37.10 | 19,469 | 19,520 | 24,258 | -0.0115 | 0.1974 | 0.1953 |
| 64 | **GD1** | 442,268,064 | 442,268,064 | 100.00 | 0.03 | 96.08 | 90.22 | 36.46 | 19,307 | 17,510 | 21,726 | 0.4268 | 0.1113 | 0.1941 |
| 65 | **FJ1** | 991,639,872 | 991,579,680 | 99.99 | 0.04 | 94.00 | 86.11 | 36.35 | 21,317 | 20,890 | 25,921 | 0.0850 | 0.1776 | 0.1941 |
| 66 | **BS1** | 515,854,944 | 515,774,592 | 99.98 | 0.04 | 94.53 | 86.59 | 36.84 | 21,003 | 21,410 | 26,553 | -0.0800 | 0.2090 | 0.1937 |
| 67 | **BS2** | 499,906,080 | 499,844,160 | 99.99 | 0.04 | 94.35 | 86.31 | 36.75 | 20,967 | 21,270 | 26,372 | -0.0591 | 0.2050 | 0.1935 |
| 68 | **YN1** | 428,009,760 | 427,995,360 | 100.00 | 0.03 | 95.94 | 89.60 | 36.39 | 20,742 | 20,470 | 25,416 | 0.0555 | 0.1839 | 0.1946 |
| 69 | **YN2** | 462,466,656 | 462,448,224 | 100.00 | 0.03 | 96.12 | 90.04 | 36.70 | 21,067 | 21,150 | 26,259 | -0.0172 | 0.1977 | 0.1946 |
| 70 | **AL1** | 473,188,320 | 473,166,720 | 100.00 | 0.03 | 96.47 | 90.99 | 37.02 | 19,488 | 17,850 | 22,199 | 0.3773 | 0.1221 | 0.1959 |
| 71 | **AL2** | 402,801,696 | 402,785,568 | 100.00 | 0.03 | 96.28 | 90.42 | 37.14 | 18,404 | 16,620 | 20,657 | 0.4418 | 0.1091 | 0.1954 |
| 72 | **AL3** | 437,963,616 | 437,944,896 | 100.00 | 0.03 | 96.24 | 90.38 | 37.10 | 19,062 | 17,340 | 21,567 | 0.4067 | 0.1161 | 0.1960 |
| 73 | **KM1** | 383,289,984 | 383,268,384 | 99.99 | 0.03 | 96.12 | 90.28 | 34.91 | 20,192 | 19,180 | 23,816 | 0.2189 | 0.1522 | 0.1947 |
| 74 | **KM2** | 368,255,232 | 368,233,632 | 99.99 | 0.03 | 95.82 | 89.41 | 36.48 | 20,497 | 19,620 | 24,372 | 0.1849 | 0.1590 | 0.1950 |
| 75 | **KM3** | 399,013,920 | 398,995,488 | 100.00 | 0.03 | 96.22 | 90.34 | 36.68 | 20,456 | 19,740 | 24,505 | 0.1505 | 0.1652 | 0.1945 |
| 76 | **DL1** | 1,348,118,208 | 1,348,032,960 | 99.99 | 0.04 | 93.95 | 86.26 | 35.61 | 21,236 | 21,720 | 26,919 | -0.0935 | 0.2111 | 0.1931 |
| 77 | **DL2** | 410,437,440 | 410,380,704 | 99.99 | 0.04 | 93.96 | 85.00 | 35.61 | 20,096 | 19,230 | 23,882 | 0.1863 | 0.1585 | 0.1948 |
| 78 | **LJ1** | 308,598,912 | 308,598,912 | 100.00 | 0.03 | 95.89 | 90.01 | 35.55 | 18,187 | 15,970 | 19,852 | 0.5713 | 0.0839 | 0.1955 |
| 79 | **LJ2** | 436,055,616 | 436,032,288 | 99.99 | 0.03 | 96.22 | 90.37 | 36.80 | 19,599 | 17,480 | 21,720 | 0.5002 | 0.0977 | 0.1952 |
| 80 | **ZD1** | 453,366,432 | 453,366,432 | 100.00 | 0.03 | 95.75 | 89.53 | 36.28 | 18,433 | 16,570 | 20,581 | 0.4646 | 0.1044 | 0.1949 |
| 81 | **ZD2** | 415178208 | 413,660,448 | 99.63 | 0.04 | 93.94 | 85.39 | 38.68 | 15,743 | 13,890 | 17,254 | 0.5503 | 0.0876 | 0.1950 |
| 82 | **CZ1** | 447,071,616 | 447,071,616 | 100.00 | 0.03 | 95.69 | 89.35 | 36.35 | 19,039 | 17,680 | 21,962 | 0.3175 | 0.1331 | 0.1950 |
| 83 | **CZ2** | 464,919,552 | 464,919,552 | 100.00 | 0.03 | 96.26 | 90.72 | 37.03 | 19,406 | 17,850 | 22,133 | 0.3631 | 0.1232 | 0.1935 |
| 84 | **CZ3** | 449,678,016 | 449,678,016 | 100.00 | 0.03 | 96.06 | 90.16 | 37.00 | 19,029 | 17,580 | 21,818 | 0.3412 | 0.1278 | 0.1942 |
| 85 | **CZ4** | 374,404,896 | 374,404,896 | 100.00 | 0.03 | 95.56 | 88.85 | 37.45 | 18,595 | 17,100 | 21,230 | 0.3627 | 0.1241 | 0.1945 |
| 86 | **CZ5** | 404,020,512 | 404,020,512 | 100.00 | 0.03 | 95.92 | 89.75 | 37.23 | 19,309 | 18,000 | 22,348 | 0.3018 | 0.1360 | 0.1946 |
| 87 | **TC1** | 384,769,728 | 384,749,280 | 99.99 | 0.03 | 96.33 | 90.44 | 37.32 | 17,916 | 16,170 | 20,090 | 0.4457 | 0.1082 | 0.1951 |
| 88 | **TC2** | 392,720,832 | 392,720,832 | 100.00 | 0.03 | 96.20 | 90.47 | 37.11 | 18,068 | 16,280 | 20,218 | 0.4542 | 0.1063 | 0.1948 |
| 89 | **TC3** | 269,707,680 | 269,707,680 | 100.00 | 0.03 | 95.22 | 88.25 | 35.70 | 16,607 | 14,750 | 18,341 | 0.5168 | 0.0945 | 0.1958 |
| 90 | **TC4** | 410,397,408 | 410,375,232 | 99.99 | 0.03 | 96.23 | 90.35 | 36.09 | 17,722 | 15,920 | 19,764 | 0.4691 | 0.1033 | 0.1945 |
| 91 | **TC5** | 455,369,760 | 455,341,248 | 99.99 | 0.03 | 96.56 | 91.22 | 36.70 | 18,311 | 16,450 | 20,413 | 0.4701 | 0.1030 | 0.1941 |
| 92 | **YJ1** | 262,158,336 | 262,158,336 | 100.00 | 0.03 | 96.28 | 90.99 | 35.83 | 15,900 | 14,100 | 17,542 | 0.5232 | 0.0936 | 0.1962 |
| 93 | **YJ2** | 422,387,136 | 422,368,704 | 100.00 | 0.03 | 96.47 | 90.89 | 36.52 | 17,628 | 15,920 | 19,798 | 0.4402 | 0.1096 | 0.1959 |
| 94 | **YJ3** | 241,341,696 | 241,341,696 | 100.00 | 0.03 | 95.81 | 89.88 | 35.59 | 15,431 | 13,670 | 17,001 | 0.5291 | 0.0923 | 0.1959 |
| 95 | **YJ4** | 416,043,360 | 416,016,288 | 99.99 | 0.03 | 96.19 | 90.38 | 36.03 | 17,212 | 15,500 | 19,263 | 0.4543 | 0.1065 | 0.1953 |
| 96 | **YJ5** | 386,351,136 | 386,351,136 | 100.00 | 0.03 | 96.05 | 90.30 | 35.55 | 17,040 | 15,330 | 19,035 | 0.4614 | 0.1048 | 0.1946 |
| 97 | **ML1** | 453,914,496 | 453,842,496 | 99.98 | 0.04 | 94.31 | 86.11 | 37.06 | 16,553 | 15,040 | 18,675 | 0.4156 | 0.1136 | 0.1946 |
| 98 | **ML2** | 272,138,112 | 272,109,888 | 99.99 | 0.05 | 94.54 | 85.00 | 36.70 | 14,965 | 13,250 | 16,463 | 0.5332 | 0.0910 | 0.1952 |
| 99 | **ML3** | 417,143,520 | 417,117,024 | 99.99 | 0.03 | 96.22 | 90.34 | 36.83 | 16,625 | 14,960 | 18,580 | 0.4597 | 0.1052 | 0.1948 |
| 100 | **ML4** | 412,412,832 | 412,389,792 | 99.99 | 0.03 | 96.09 | 90.04 | 37.08 | 16,362 | 14,790 | 18,365 | 0.4390 | 0.1091 | 0.1947 |
| 101 | **ML5** | 433,741,248 | 433,680,768 | 99.99 | 0.04 | 94.47 | 86.44 | 36.95 | 16,586 | 15,070 | 18,680 | 0.4207 | 0.1121 | 0.1933 |
| 102 | **NN1** | 409,080,096 | 409,054,176 | 99.99 | 0.03 | 96.28 | 90.34 | 36.84 | 17,386 | 15,460 | 19,242 | 0.5092 | 0.0965 | 0.1965 |
| 103 | **NN2** | 455,437,440 | 455,408,928 | 99.99 | 0.03 | 96.34 | 90.57 | 35.97 | 19,663 | 18,860 | 23,435 | 0.1748 | 0.1610 | 0.1952 |
| 104 | **NN3** | 500,868,576 | 500,785,056 | 99.98 | 0.04 | 94.86 | 87.48 | 36.75 | 20,205 | 19,500 | 24,218 | 0.1493 | 0.1657 | 0.1948 |
| 105 | **HN1** | 442,704,384 | 442,704,384 | 100.00 | 0.03 | 96.09 | 90.24 | 36.56 | 17,853 | 15,990 | 19,867 | 0.4800 | 0.1014 | 0.1951 |
| 106 | **HN2** | 1,677,309,696 | 1,677,206,304 | 99.99 | 0.04 | 94.48 | 87.04 | 36.42 | 19,911 | 18,510 | 22,954 | 0.3154 | 0.1326 | 0.1936 |
| 107 | **HN3** | 465,772,608 | 465,772,608 | 100.00 | 0.03 | 96.21 | 90.43 | 36.56 | 17,881 | 16,020 | 19,925 | 0.4762 | 0.1026 | 0.1960 |
| **Average** |  | 464,825,930 | 464,783,950 | 99.99 | 0.03 | 95.59 | 88.96 | 36.66 | 19,821 | 18,755 | 23,279 | 0.2519 | 0.1454 | 0.1944 |

**Table S17. GBS sample information and genetic diversity of G. pentaphyllum populations**

| **No.** | **ID** | **Location** | **latitude** | **longitude** | **Ploidy** | **Sample size** | **Ho** | **He** | **F** |
| --- | --- | --- | --- | --- | --- | --- | --- | --- | --- |
| 1 | WS | Hangzhou, Zhejiang | 30.24° N | 120.16° E | 2 | 3 | 0.1159 | 0.1945 | 0.4039 |
| 2 | XC | Xuancheng, Anhui | 30.947° N | 118.76° E | 2 | 5 | 0.1479 | 0.1938 | 0.2364 |
| 3 | ST | Shitai, Anhui | 30.196° N | 117.53° E | 2 | 5 | 0.1238 | 0.1940 | 0.3622 |
| 4 | WN | Wuning, Jiangxi | 29.318° N | 115.09° E | 2 | 5 | 0.1555 | 0.1935 | 0.1967 |
| 5 | XY | Xinyang, Henan | 32.141° N | 114.09° E | 2 | 3 | 0.1310 | 0.1939 | 0.3242 |
| 6 | HB | Zhushan, Hubei | 32.228° N | 110.22° E | 2 | 2 | 0.0993 | 0.1943 | 0.4891 |
| 7 | YF | Pingli, Shaanxi | 32.21° N | 109.17° E | 4 | 5 | 0.1308 | 0.1932 | 0.3229 |
| 8 | RH | Renhuai, Guizhou | 27.837° N | 106.4° E | 4 | 5 | 0.1413 | 0.1941 | 0.2719 |
| 9 | ZT | Zhaotong, Yunnan | 27.352° N | 103.72° E | 2 | 5 | 0.0976 | 0.1935 | 0.4957 |
| 10 | JY | Beibei, Chongqing | 29.835° N | 106.39° E | 2 | 3 | 0.1741 | 0.1940 | 0.1025 |
| 11 | JS | Jishou, Hunan | 28.286° N | 109.70° E | 6 | 2 | 0.1733 | 0.1953 | 0.1126 |
| 12 | ES | Enshi, Hubei | 30.277° N | 109.49° E | 2 | 3 | 0.2210 | 0.1937 | -0.1406 |
| 13 | HS | Zhangjiajie, Hunan | 29.218° N | 110.46° E | 2 | 5 | 0.2142 | 0.1941 | -0.1035 |
| 14 | ZZ | Zhuzhou, Hunan | 27.842° N | 113.13° E | 2 | 1 | 0.1907 | 0.1950 | 0.0213 |
| 15 | SX | Shaxian, Fujian | 26.401° N | 117.79° E | 2 | 4 | 0.1979 | 0.1951 | -0.0146 |
| 16 | DY | Dayu, Jiangxi | 25.389° N | 114.09° E | 2 | 3 | 0.2096 | 0.1940 | -0.0800 |
| 17 | LZ | Liuzhou, Guangxi | 24.285° N | 109.64° E | 2 | 4 | 0.2019 | 0.1948 | -0.0361 |
| 18 | GD | Guangdong, Guangzhou | 23.178° N | 113.28° E | 2 | 1 | 0.1113 | 0.1941 | 0.4268 |
| 19 | FJ | Fanjingshan, Guizhou | 27.92° N | 108.70° E | 2 | 1 | 0.1776 | 0.1941 | 0.0850 |
| 20 | BS | Baise, Guangxi | 23.917° N | 106.62° E | 4 | 2 | 0.2070 | 0.1936 | -0.0695 |
| 21 | YN | HaGiang, Vietnam | 22.766° N | 104.94° E | 2 | 2 | 0.1908 | 0.1946 | 0.0192 |
| 22 | AL | Jiayi, Taiwan | 23.511° N | 120.8° E | 2 | 3 | 0.1158 | 0.1958 | 0.4086 |
| 23 | KZ | Kunming, Yunnan | 25.162° N | 102.74° E | 4 | 3 | 0.1588 | 0.1947 | 0.1848 |
| 24 | DL | Dali, Yunnan | 25.634° N | 100.27° E | 8 | 2 | 0.1848 | 0.1940 | 0.0464 |
| 25 | LJ | Xichang, Sichuan | 27.853° N | 102.3° E | 2 | 2 | 0.0908 | 0.1954 | 0.5358 |
| 26 | ZD | Zhongdian, Yunnan | 27.182° N | 100.05° E | 2 | 2 | 0.0960 | 0.1949 | 0.5075 |
| 27 | CZ | Cizhong, Yunnan | 28.027° N | 98.91° E | 2 | 5 | 0.1288 | 0.1944 | 0.3373 |
| 28 | TC | Tengchong, Yunnan | 25.106° N | 98.51° E | 2 | 5 | 0.1031 | 0.1949 | 0.4712 |
| 29 | YJ | Yingjiang, Yunnan | 24.613° N | 97.66° E | 6 | 5 | 0.1014 | 0.1956 | 0.4816 |
| 30 | ML | Mengla, Yunnan | 21.566° N | 101.57° E | 2 | 5 | 0.1062 | 0.1945 | 0.4536 |
| 31 | NN | Jinghong, Yunnan | 21.937° N | 100.61° E | 2 | 3 | 0.1410 | 0.1955 | 0.2778 |
| 32 | HN | Wuzhishan, Hainan | 18.776° N | 109.52° E | 2 | 3 | 0.1122 | 0.1949 | 0.4239 |

**Table S18. Metrics of niche dynamics comparing pairs of G. pentaphyllum ranges (a→b).**

| Distribution ranges |  | Niche Overlap (D) | Equivalency test (*p*-value) |  | Similarity test (p-value) |  | Niche unflling | Niche stability | Niche expansion |
| --- | --- | --- | --- | --- | --- | --- | --- | --- | --- |
| a | b |  | equivalent | non equivalent | similar | different |  |  |  |
| Group N | Group SE | Na | 1 | 0.091 | 0.455 | 0.818 | 0.456 | 0.615 | 0.385 |
|  | Group SW | Na | 1 | 0.091 | 0.364 | 0.364 | 1 | 0 | 1 |
| Group SE | Group N | 0.083 | 0.909 | 0.182 | 0.091 | 0.818 | 0.385 | 0.544 | 0.456 |
|  | Group SW | Na | 1 | 0.091 | 1 | 0.455 | 1 | 0 | 1 |
| Group SW | Group N | 0 | 1 | 0.091 | 0.455 | 1 | 1 | 0 | 1 |
|  | Group SE | 0 | 1 | 0.091 | 1 | 1 | 1 | 0 | 1 |
