# Supplementary figures and images for: Diploid chromosome-level reference genome and population genomic analyses provide insights into Gypenoside biosynthesis and demographic evolution of Gynostemma pentaphyllum (Cucurbitaceae)
Source: Hortic Res. 2022 Oct 19;10(1):uhac231. doi: 10.1093/hr/uhac231 (PMC9832869; doi:10.1093/hr/uhac231)

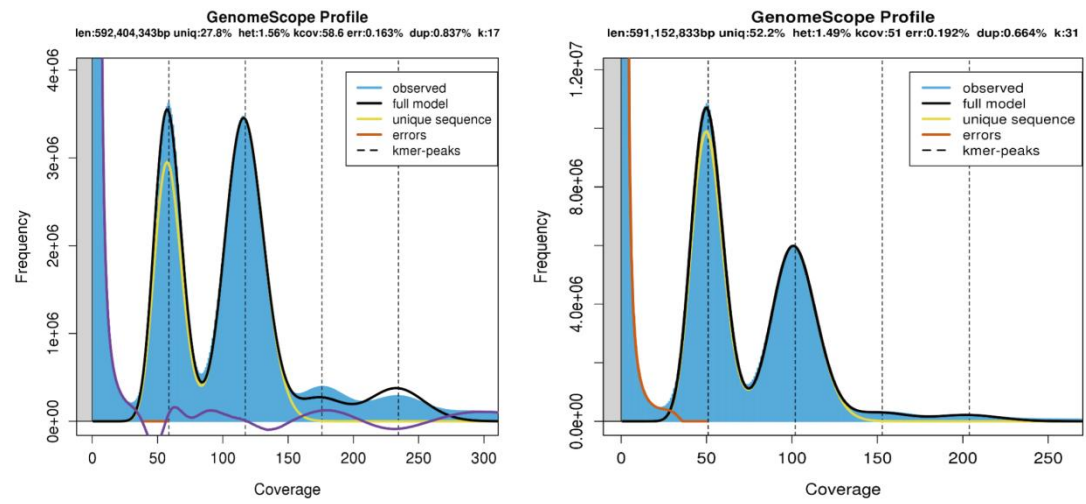

**Figure S1**

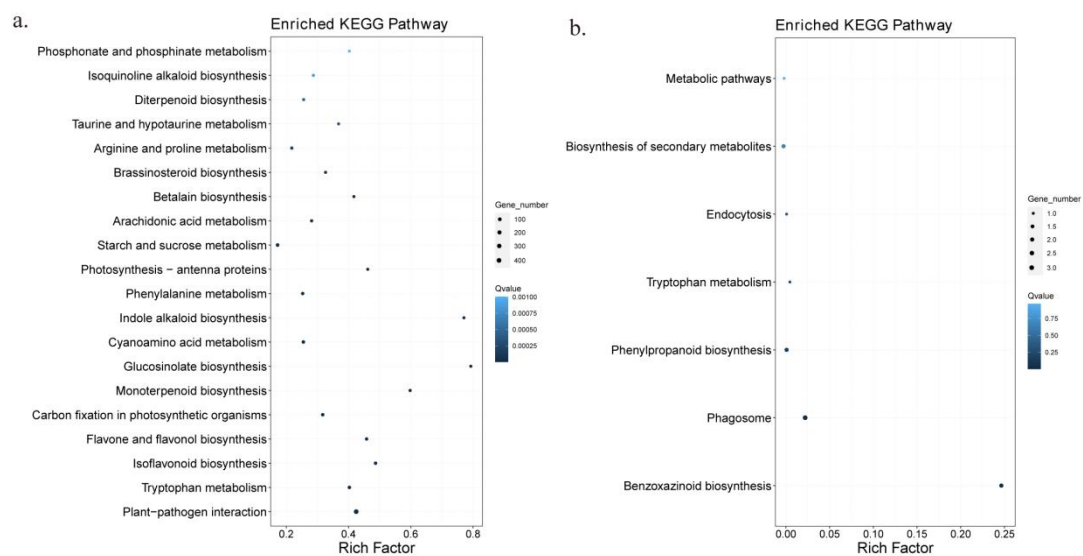

**Figure S2**

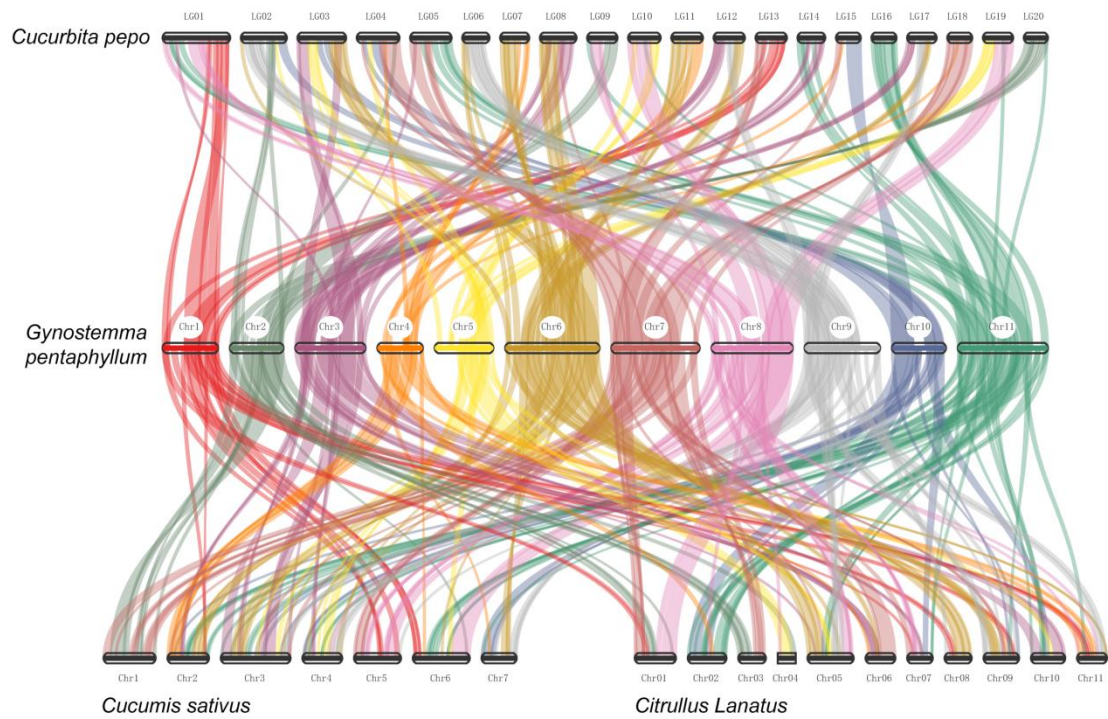

**Figure S3**

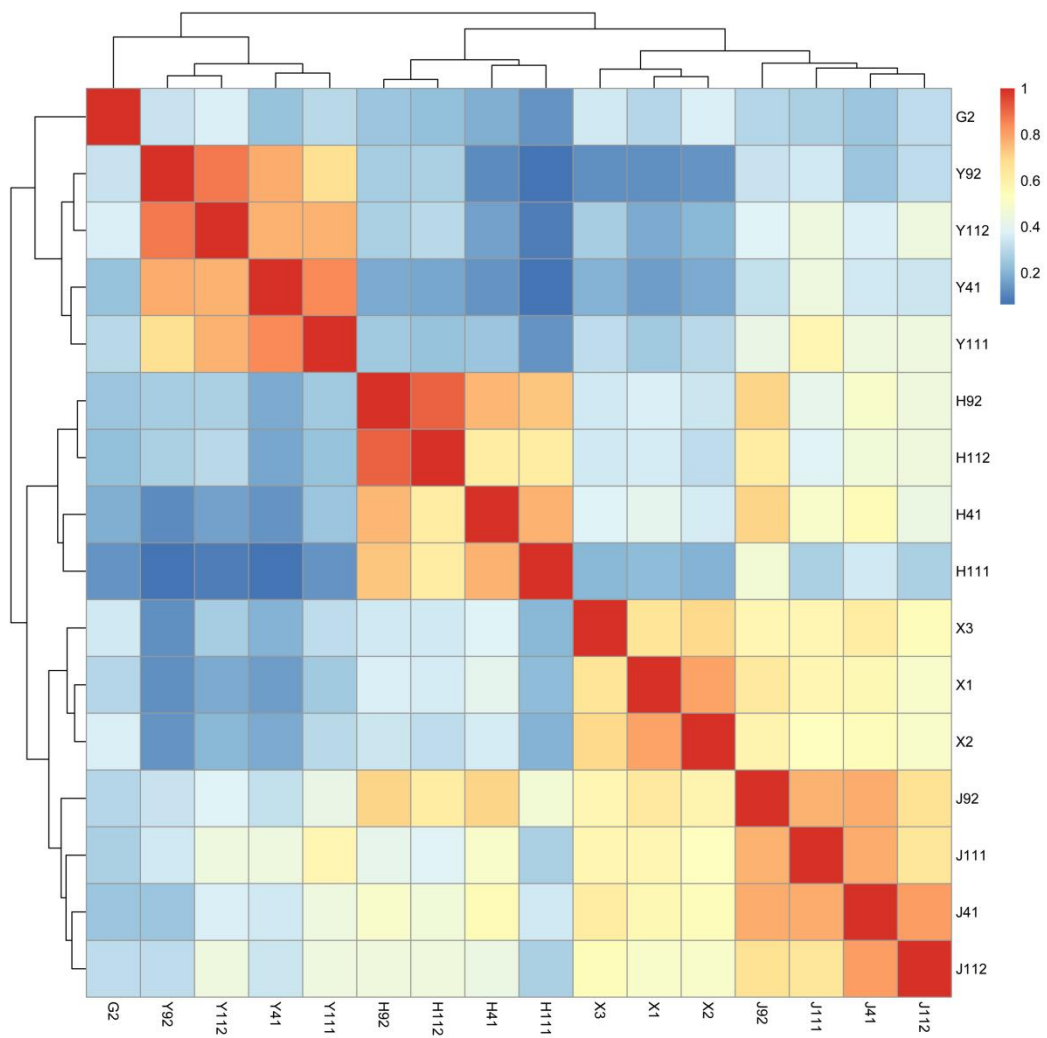

**Figure S4**

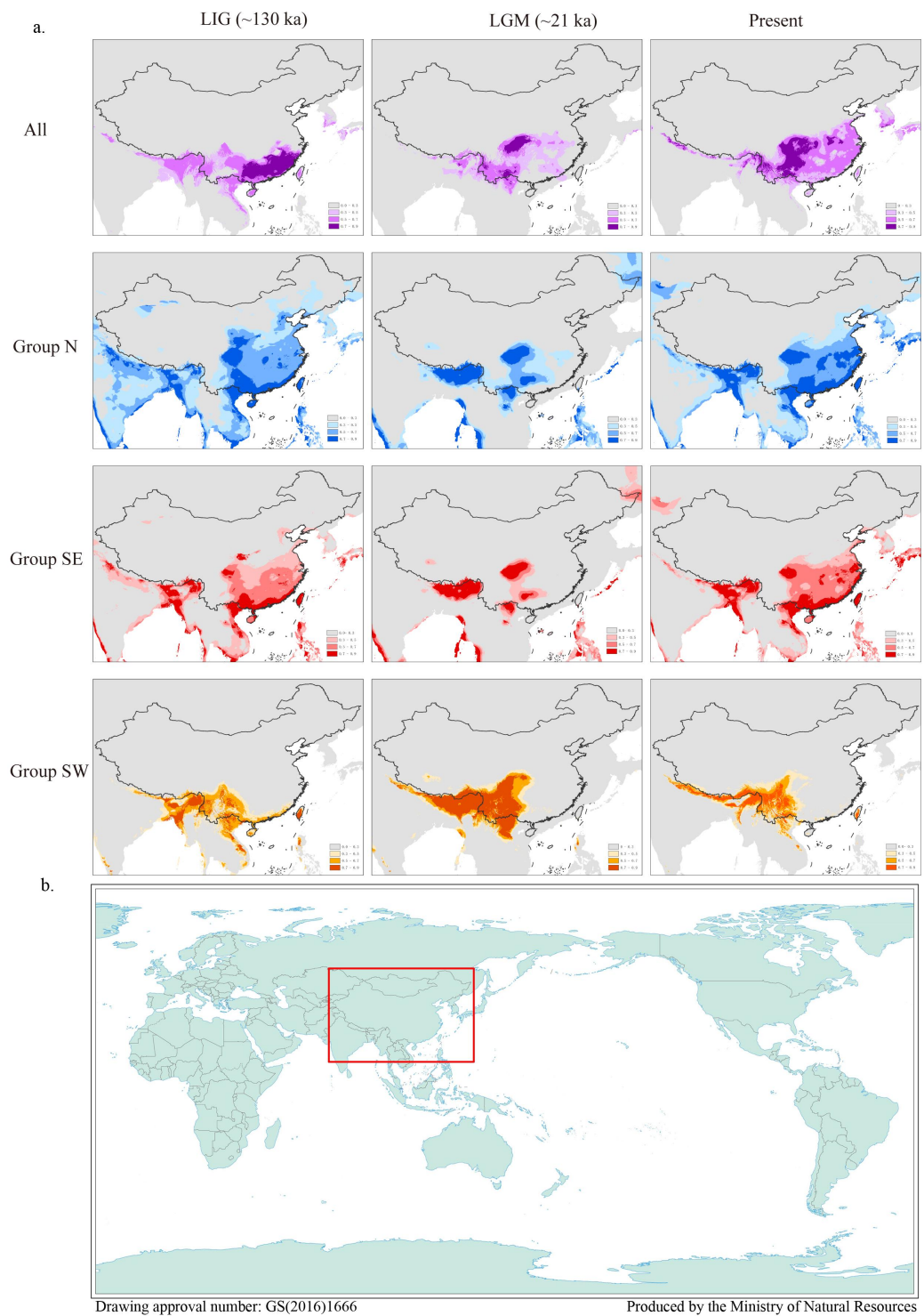

**Figure S5**

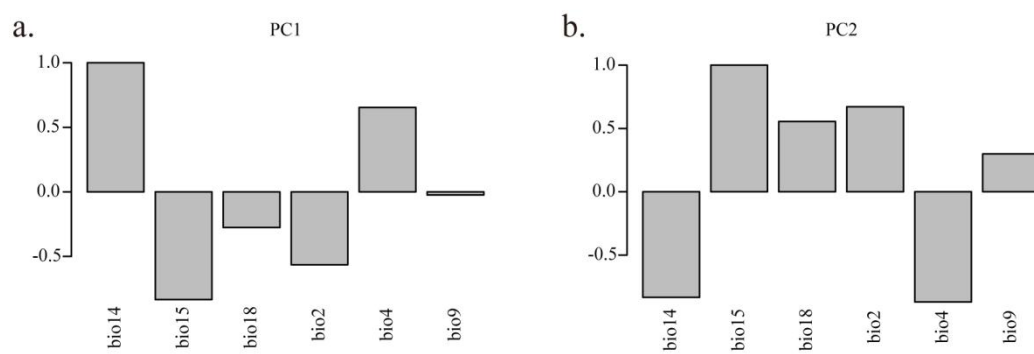

**Figure S6**

Supplement: Web_Material_uhac231 [file web_material_uhac231.zip › FigureS.R1.pdf]
